# National pathways of land-use CO₂ emissions in the 21^st^ century

**DOI:** 10.1038/s41467-026-74836-w

**Published:** 2026-06-24

**Authors:** Danni Zhang, Bo Zheng, Yue He, Tianyi Wang, Gaurav P. Shrivastav, Philippe Ciais, Thomas Gasser

**Affiliations:** 1 Shenzhen Key Laboratory of Ecological Remediation and Carbon Sequestration, Institute of Environment and Ecology, Tsinghua Shenzhen International Graduate School, Tsinghua University, Shenzhen, China; 2 International Institute for Applied Systems Analysis, Laxenburg, Austria; 3 State Environmental Protection Key Laboratory of Sources and Control of Air Pollution Complex, Beijing, China; 4 Institute of Carbon Neutrality, Peking University, Beijing, China; 5 Laboratoire des Sciences du Climat et de l’Environnement, CEA–CNRS–UVSQ, IPSL, Université Paris-Saclay, Gif-sur-Yvette, France

**Keywords:** Climate sciences, Environmental sciences

## Abstract

Land-use and land-cover change (LULCC) is a major source of anthropogenic CO₂ emissions, yet projections remain scarce. Here, we use the reduced-complexity Earth system model OSCAR to generate national LULCC carbon emission trajectories through 2100, across 150 socioeconomic and policy-relevant scenarios. Deforestation and forest regrowth dominate variability in LULCC carbon emission, with policy timing and ambition exerting strong control. Ending gross deforestation by 2030 yields large, persistent removals (about −30 Pg C by 2100), whereas net forest area balance still emits 4–9 Pg C. The strongest sinks are projected to emerge in China and Indonesia, while Brazil and the Democratic Republic of the Congo dominate global sources. The accompanying open dataset enables country-level scenario assembly and policy evaluation. Our findings underscore that early and ambitious land governance, particularly in tropical regions, is essential for transforming the land sector into a durable carbon sink aligned with global temperature goals.

## Abstract (web summary)

The authors use a compact Earth system model OSCAR across SSP and idealized scenarios to show that policy timing and ambition are the primary determinants of whether the land sector becomes a durable carbon sink or remains a persistent source.

## Introduction

Land use and land-cover change (LULCC) have been a major driver of global anthropogenic carbon emissions^1–3^ and play a crucial role in shaping climate change^4^. LULCC accounts for 35% of cumulative anthropogenic CO₂ emissions since preindustrial times, second only to fossil fuel combustion^4,5^. Achieving net-zero or net-negative emissions in the LULCC sector is key to the feasibility of the Paris Agreement targets^6,7^.

Projecting future LULCC-related CO₂ fluxes is challenging, notably owing to the ambiguities in how the scientific community, modeling platforms, and experimental protocols define and quantify the anthropogenic (LULCC emissions, ${E}_{{{\rm{LUC}}}}$) versus natural (land carbon sink, ${S}_{{{\rm{land}}}}$) components of the land carbon cycle^2,8,9^. The fundamental reason behind this definitional inconsistency is that, while it is conceptually very clear that ${E}_{{{\rm{LUC}}}}$ and ${S}_{{{\rm{land}}}}$ should refer to the direct and indirect anthropogenic perturbations, respectively, these two interact synergistically due to the system’s nonlinearity^2^. It is physically impossible to observe the impact of individual perturbations separately, and only carefully designed numerical models and experimental protocols can estimate each term. These difficulties have resulted in a lack of estimates of ${E}_{{{\rm{LUC}}}}$ components over the future by Working Group 1 of the IPCC in their 6^th^ assessment report^10^ and a lack of estimates of the CO₂ removal from afforestation and reforestation methods by Working Group 3^11^.

National greenhouse gas inventories consider carbon fluxes occurring on managed land as anthropogenic, thereby combining ${E}_{{{\rm{LUC}}}}$ and ${S}_{{{\rm{land}}}}$ components^12,13^. In contrast, model-based frameworks distinguish between direct anthropogenic fluxes (${E}_{{{\rm{LUC}}}}$) and the natural land sink (${S}_{{{\rm{land}}}}$)^9,14,15^. This definitional mismatch leads to substantial differences between inventory-based accounting and model-based estimates. The Global Carbon Budget (GCB) addresses this discrepancy by combining bookkeeping model estimates of ${E}_{{{\rm{LUC}}}}$^16–18^ with ${S}_{{{\rm{land}}}}$ simulated by Dynamic Global Vegetation Models (DGVMs)^19–21^. Within this framework,${E}_{{{\rm{LUC}}}}$ is interpreted as the component of land carbon flux directly attributable to anthropogenic land-use activities, while ${S}_{{{\rm{land}}}}$ is treated as an indirect response driven by atmospheric CO₂ increase and climate change. This approach facilitates a dialog between the scientific and policy communities^4,13^, but is possible in the future only with a model that consistently represents both terms of the equation^22^.

Here, we use the reduced-complexity Earth system model OSCAR and its proven capacity to bridge across definitional differences^2,15,22,23^ to generate a comprehensive set of national trajectories of land-use and land-cover change (LULCC) carbon fluxes to 2100 across 150 scenarios spanning socioeconomic pathways, climate futures, and policy interventions. We focus on ${E}_{{LUC}}$, defined consistently with the bookkeeping framework of the GCB^4^, while also reporting estimates of the land sink under pre-industrial land cover (${S}_{{{{\rm{land}}}},{{{\rm{PI}}}}}$) and under actual land cover (${S}_{{{{\rm{land}}}},{{{\rm{actual}}}}}$) to facilitate comparison with national inventories. These estimates extend the historical estimates generated with OSCAR for the latest GCB2024, up to the year 2100 and at the national level. The 150 scenarios integrate uncertainties in socioeconomic pathways, climate projections, and policy-relevant LULCC interventions, including eight Shared Socioeconomic Pathways (SSPs) land-use scenarios and seven idealized land-use scenarios designed to isolate the effects of specific interventions. These scenarios are implemented in OSCAR, harmonized with the historical GCB, and driven by two climate pathways using bias-corrected projections from five Earth system models. LULCC forcing is provided by the Land-Use Harmonization 2 (LUH2) dataset for CMIP6, updated for GCB2024^4,24^ and a complementary historical dataset derived from Food and Agriculture Organization (FAO) Forest Resource Assessments, while climate inputs are taken from Climatic Research Unit Time Series (CRU-TS) for the historical period and Inter-Sectoral Impact Model Intercomparison Project 3b (ISIMIP 3b) for the future. By systematically projecting possible futures, our analysis reveals how different national land-use pathways could contribute to the global mitigation challenge, offering a foundation for designing more equitable and forward-looking land-based strategies within a limited remaining global carbon budget.

## Results

### Overview of the main flux behavior

The low-warming scenario is based on the climate and atmospheric CO₂ conditions of SSP1-2.6, whereas the high-warming scenario follows SSP3-7.0. Each warming scenario is combined with alternative LULCC pathways to disentangle the relative contributions of land-use decisions and warming forcing to terrestrial carbon fluxes. The comparison between SSP1-2.6 and SSP3-7.0 LULCC under the fixed low-warming isolates the influence of land-use trajectories. This comparison minimizes warming-driven feedbacks and allows differences in carbon fluxes to be attributed primarily to contrasting land-use strategies. We then quantify the effect of climate change by holding the LULCC trajectory constant (SSP3-7.0) and comparing results under low- and high-warming conditions. Here, negative values indicate carbon removals from the atmosphere, while positive values indicate emissions.

**Fig. 1:**
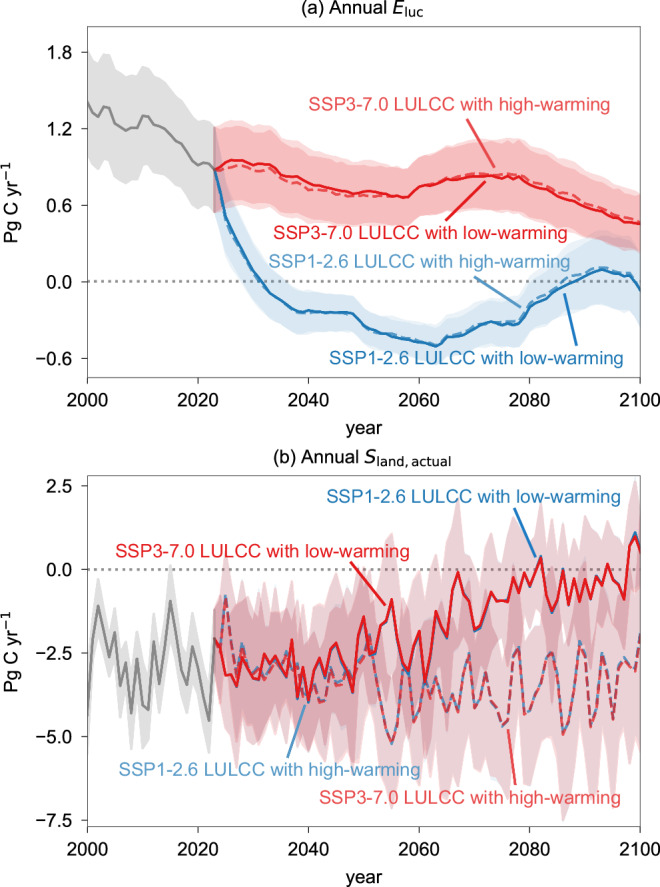
Main carbon fluxes under SSP1-2.6 and SSP3-7.0 land-use and land-cover change (LULCC) scenarios. **a** LULCC emissions (${E}_{{{\rm{LUC}}}}$) changes from 2000 to 2100 and **b** the natural land sink under evolving land cover (${S}_{{{{\rm{land}}}},{{{\rm{actual}}}}}$) changes from 2000–2100. The gray line with shading shows OSCAR estimates in 2000–2023 for the Global Carbon Budget. Blue lines indicate projected values under the SSP1-2.6 LULCC scenario, and red lines indicate projected values under the SSP3-7.0 LULCC scenario. Solid lines correspond to the low-warming pathway and dashed lines to the high-warming pathway. Shaded envelopes represent the mean ±1 standard deviation (SD), shown as hatched shading for the SSP1-2.6 LULCC scenario and solid shading for the SSP3-7.0 LULCC scenario.

Land-use choices over climate forcing shape ${E}_{{LUC}}$ throughout the 21st century, with forest-area dynamics remaining the dominant driver of emissions. From 2024 to 2100, the SSP1-2.6 LULCC scenario under low-warming results in the global forest expansion of approximately 53 Mha and a cumulative ${E}_{{{{\rm{LUC}}}}}$ of –14 Pg C. The SSP3-7.0 LULCC scenario produces a forest loss of 321 Mha, resulting in a cumulative ${E}_{{{{\rm{LUC}}}}}$ of 57 Pg C under low-warming. Differences in atmospheric CO₂ concentrations and temperature exert a secondary influence, as cumulative ${E}_{{{\rm{LUC}}}}$ values remain nearly identical between low- and high-warming conditions for a given LULCC pathway (Fig. 1a).

In contrast, ${S}_{{{{\rm{land}}}},{{{\rm{actual}}}}}$ is primarily governed by atmospheric CO₂ and climate rather than by LULCC trajectories (Fig. 1b). Under the low-warming scenario, ${S}_{{{{\rm{land}}}},{{{\rm{actual}}}}}$ in both the SSP1-2.6 and SSP3-7.0 LULCC scenarios accumulate to approximately –129 Pg C. Under high warming, the sink intensifies markedly to –256 Pg C and –260 Pg C, respectively. Meanwhile, the loss of additional sink capacity (${F}_{{{{\rm{LASC}}}}}$) differs between the SSP1-2.6 (16 Pg C under low warming and 23 Pg C under high warming) and SSP3-7.0 (18 Pg C and 35 Pg C) LULCC scenarios, demonstrating an interaction between LULCC and climate in shaping ${S}_{{{{\rm{land}}}},{{{\rm{actual}}}}}$.

As a result of these combined processes, cumulative land carbon flux (${F}_{{{{\rm{net}}}}}$) shows large scenario-dependent contrasts. Under SSP1-2.6 LULCC scenarios, the cumulative ${F}_{{{{\rm{net}}}}}$ ranges from –142 ± 58 Pg C (low-warming) to –269 ± 112 Pg C (high-warming). Under SSP3-7.0 LULCC scenarios, the cumulative uptake ranges from –70 ± 58 Pg C (low-warming) to –203 ± 116 Pg C (high-warming). In OSCAR, the global carbon balance of these scenarios is dominated by the land sink and specifically the CO₂-fertilization effect, which is consistent with the CMIP6-reported ranges (–167 ± 66 Pg C in SSP1-2.6 and –217 ± 126 Pg C in SSP3-7.0)^25^.

Variations in ${S}_{{{\rm{land}}}}$ represent an externally forced response rather than a policy-controlled flux, and we therefore focus on ${E}_{{{\rm{LUC}}}}$ as the appropriate metric to quantify the impact of land-use policy. Given its limited sensitivity to climate feedbacks, we focus in the following sections on ${E}_{{{\rm{LUC}}}}$. All subsequent analyses of shifted SSP and idealized LULCC scenarios are conducted under the low-warming climate and atmospheric CO₂ pathway. Results for all other combinations of climate and LULCC scenarios are provided in the accompanying dataset (10.5281/zenodo.16729725).

### Shifted SSP scenarios under low warming

The shifted SSP scenarios under low warming represent internally consistent LULCC trajectories derived from Integrated Assessment Models (IAMs), in which the original SSP land-use time series (starting in 2015) are temporally realigned with the observation-constrained historical period ending in 2023. These scenarios provide a benchmark for comparing OSCAR-based land-use carbon fluxes with IAM-based expectations under contrasting socio-economic pathways. The resulting forest-area trajectories are summarized in Supplementary Table 4.

**Fig. 2:**
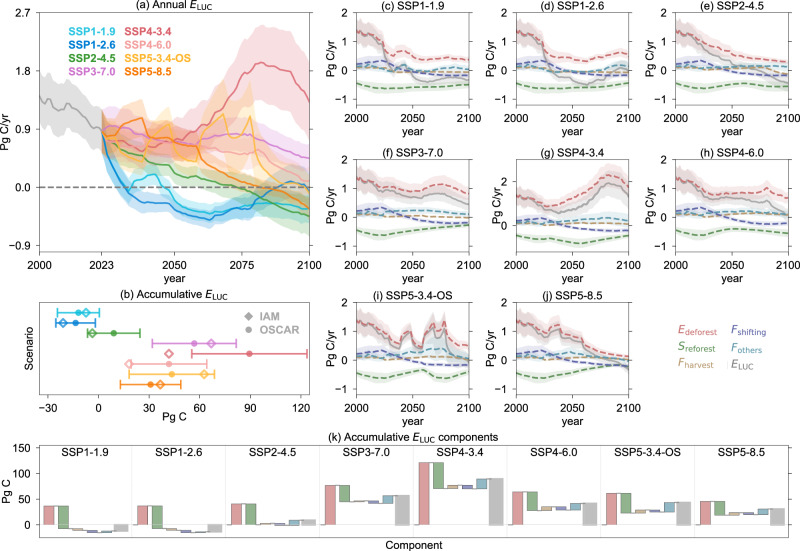
Global land-use and land-cover change emissions (*E*_LUC_) under shifted SSP land-use and land-cover change (LULCC) scenarios driven by the low-warming climate and atmospheric CO₂ forcing. **a** Annual global ${E}_{{{\rm{LUC}}}}$ under shifted SSP scenarios in 2000–2100. The gray line with shading shows OSCAR estimates in 2000–2023 for the Global Carbon Budget. **b** Accumulative global ${E}_{{{\rm{LUC}}}}$ under shifted SSP scenarios in 2024–2100. Circles mark values in this study, and vertical error bars indicate ±1 standard deviation (SD). The diamond markers indicate the accumulative global ${E}_{{{\rm{LUC}}}}$ simulated by the IAMs (Integrated Assessment Models) over the native scenario period (2015–2091), used here for reference. **c**–**j** Annual global ${E}_{{{\rm{LUC}}}}$ and its components from 2000 to 2100, by scenario: **c** SSP1-1.9, **d** SSP1-2.6, **e** SSP2-4.5, **f** SSP3-7.0, **g** SSP4-3.4, **h** SSP4-6.0, **i** SSP5-3.4-OS, **j** SSP5-8.5. In each panel, the gray solid line is total ${E}_{{{\rm{LUC}}}}$ and colored dashed lines are component fluxes, including emissions from deforestation (${E}_{{{\rm{deforest}}}}$), removals from afforestation/reforestation (${S}_{{{\rm{reforest}}}}$), net flux from wood harvest (${F}_{{{\rm{harvest}}}}$), net flux from shifting cultivation (${F}_{{{\rm{shifting}}}}$), and flux from all other land use and land-cover change (${F}_{{{\rm{others}}}}$). **k** Accumulative global ${E}_{{{\rm{LUC}}}}$ component under shifted SSP scenarios in 2024–2100. Stacked colored bars show component contributions and gray bars show total accumulative ${E}_{{{\rm{LUC}}}}$. Shaded envelopes in (**a**) and in (**c**–**j**) represent the mean ± one SD. Exact cumulative values for each ${E}_{{{\rm{LUC}}}}$ component and scenario are reported in Supplementary Table 2.

${E}_{{{\rm{LUC}}}}$ diverges strongly across SSP scenarios (Fig. 2a), reflecting a complex interplay between the climate target, the socio-economic narrative, and the IAMs underlying each SSP^26^. Cumulative ${E}_{{{\rm{LUC}}}}$ from IAMs are taken over their native scenario period (2015–2091) and used as a benchmark to contextualize OSCAR-based estimates under the corresponding shifted SSP land-use pathways. Our cumulative ${E}_{{{\rm{LUC}}}}$ estimates broadly align with IAM results, though OSCAR produces higher values under SSP4 than reported by the IAM (Fig. 2b). There are several sources of discrepancy, such as assumptions on carbon densities, land-use processes representation, alteration of the IAM data through the harmonization process^27^, a shift in the starting year (“Methods”) and influence of historical land-use. Therefore, an exact quantitative match between our simulations and the estimates originally reported by the IAM should not be expected and is not the goal of this work.

Under SSP1-1.9 and SSP1-2.6, cumulative ${E}_{{{\rm{LUC}}}}$ values between 2024 and 2100 are –12 Pg C and –14 Pg C, respectively (Fig. 2a). Intermediate- and high-emission SSPs prescribe weaker land-use mitigation and exhibit more intensive land-use emissions. Under SSP2-4.5, global ${E}_{{{\rm{LUC}}}}$ yields a modest cumulative emission of 9 Pg C by 2100. In the overshoot and fossil-fuel development scenarios (SSP5-3.4-OS and SSP5-8.5), the cumulative emission totals are 43 Pg C and 31 Pg C, respectively. The strongest and most persistent land-use emissions occur in SSP3-7.0 and SSP4, which combine limited international cooperation with regionally fragmented land management. Cumulative emissions reach 57 Pg C under SSP3-7.0, 42 Pg C under SSP4-6.0, and a striking 89 Pg C under SSP4-3.4.

Across all scenarios, deforestation remains the dominant carbon source, while forest regrowth constitutes the primary removal (Fig. 2c–j, Supplementary Table 2). Contributions from wood harvest, shifting cultivation, and other land-use transitions are secondary, varying with regional management practices and policy stringency. Under SSP1 pathways, emissions from deforestation are largely compensated by substantial carbon uptake from forest regrowth, with additional smaller sinks arising from reduced wood harvest and shifting cultivation. As a result, LULCC emissions are close to balance or slightly negative over 2024–2100. Under SSP2-4.5, the narrowing balance between forest loss and regrowth, combined with positive contributions from harvest and other transitions, leads to a small carbon source. Under SSP3-7.0, cumulative deforestation emissions increase sharply, while forest regrowth weakens, reinforcing a strong source. Similar but more extreme dynamics emerge under SSP4, reflecting divergent land-use governance across regions. In SSP4-3.4, extensive deforestation is only partly offset by regrowth, resulting in the largest cumulative source among all scenarios, whereas SSP4-6.0 yields smaller but still substantial emissions. Fossil-fuel-driven SSP5 pathways exhibit comparable balances between forest loss and regrowth, but sustain land-use emissions due to delayed recovery and continued harvest activities.

**Fig. 3:**
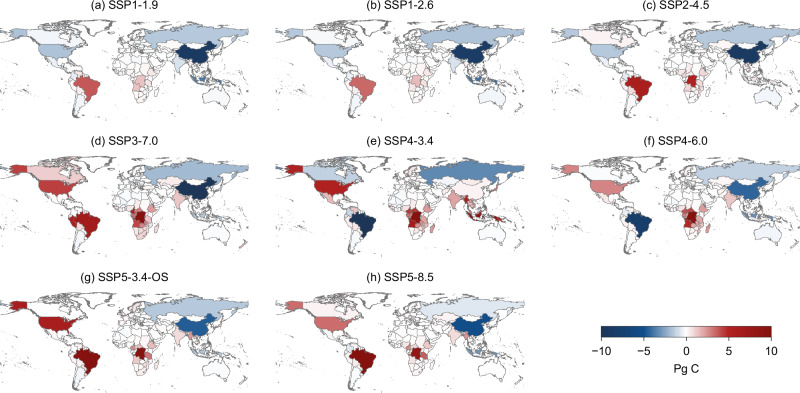
Regional cumulative land-use and land-cover change emissions (*E*_LUC_) from 2024–2100 under shifted SSP land-use and land-cover change (LULCC) scenarios driven by low-warming climate and atmospheric CO₂ forcing. Maps show regional cumulative emissions at 2100 for: **a** SSP1-1.9, **b** SSP1-2.6, **c** SSP2-4.5, **d** SSP3-7.0, **e** SSP4-3.4, **f** SSP4-6.0, **g** SSP5-3.4-OS, **h** SSP5-8.5. The shared color bar indicates net cumulative emissions. Blue tones denote net regional carbon removal (negative emissions) and red tones denote net regional carbon release (positive emissions), with white near zero. Map boundaries are sourced from the Resource and Environmental Science Data Platform (https://www.resdc.cn).

At the regional scale, sustainable SSP1 scenarios show China and Indonesia emerging as dominant land-use carbon sinks, with the additional smaller sinks in the United States, Russia, and India (Fig. 3a, b, Supplementary Table 3). Tropical regions such as Brazil, the Democratic Republic of the Congo (COD), and Angola remain persistent sources, indicating continued deforestation pressure even under sustainable development narratives. Under SSP2 and SSP3 (Fig. 3c, d), weakening land-use regulation leads to increasingly uneven spatial patterns, with Brazil and the COD becoming dominant global sources, while China maintains substantial sink capacity and Southeast Asia provides only partial compensation. Divergent outcomes are most pronounced under SSP4 (Fig. 3f, g), where the contrasting regional policy regimes produce simultaneous large sinks and sources. In SSP5 pathways (Fig. 3h), Brazil and the COD remain leading emitters, while China and Indonesia retain weakened but still positive sink roles.

### Idealized scenarios under low warming

In addition to the shifted SSP pathways, we designed seven idealized scenarios to explore alternative LULCC pathways (Supplementary Fig. 2). The Commit scenario ceases all LULCC to isolate legacy carbon dynamics, while BAU2030 extends current trends to 2030 and phases out land-cover change by 2050. Forest2100 intensifies afforestation on pasture to reach 935 Mha of forest by 2100. The Gross2030 and Gross2050 scenarios halt gross deforestation by 2030 or 2050, and Net2030 and Net2050 balance deforestation with afforestation by those years, all relative to BAU2030. These scenarios isolate the effects of policy stringency and implementation timing on land-use carbon fluxes by imposing simplified, physically consistent constraints on forest loss and gain.

**Fig. 4:**
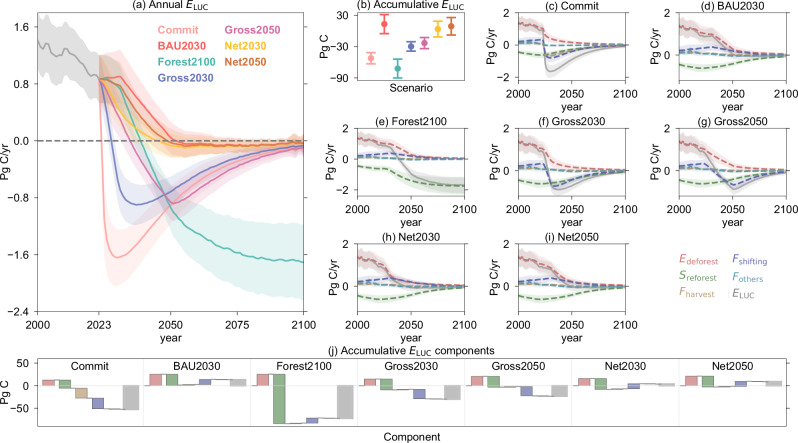
Global land-use and land-cover change emissions (*E*_LUC_) under idealized land-use and land-cover change (LULCC) scenarios driven by low-warming climate and atmospheric CO₂ forcing. **a** Annual global ${E}_{{{\rm{LUC}}}}$ from 2000 to 2100 for seven idealized scenarios (Commit: immediate cessation of all LULCC from 2023; BAU2030: Business As Usual baseline; Forest2100: intense afforestation to a constrained global maximum forest area by 2100; Gross2030/2050: all gross deforestation halted by 2030/2050; Net2030/2050: net deforestation halted by 2030/2050). The gray line with shading shows OSCAR estimates in 2000–2023 for the Global Carbon Budget. **b** Accumulative global ${E}_{{{\rm{LUC}}}}$ in 2024–2100 for each idealized scenario (left to right: Commit, BAU2030, Forest2100, Gross2030, Gross2050, Net2030, Net2050). Circles mark values at 2100, and vertical error bars indicate ±1 standard deviation (SD). **c**–**i** Annual global ${E}_{{{\rm{LUC}}}}$ and its components from 2000 to 2100, by scenario: **c** Commit, **d** BAU2030, **e** Forest2100, **f** Gross2030, **g** Gross2050, **h** Net2030, **i** Net2050. In each panel, the gray solid line is total ${E}_{{{\rm{LUC}}}}$ and colored dashed lines are component fluxes, including emissions from deforestation (${E}_{{{\rm{deforest}}}}$), removals from afforestation/reforestation (${S}_{{{\rm{reforest}}}}$), net flux from wood harvest (${F}_{{{\rm{harvest}}}}$), net flux from shifting cultivation (${F}_{{{\rm{shifting}}}}$), and flux from all other land use and land-cover change (${F}_{{{\rm{others}}}}$). **j** Accumulative global ${E}_{{{\rm{LUC}}}}$ component in 2024–2100 for each idealized scenario. Stacked colored bars show component contributions and gray bars show total accumulative ${E}_{{{\rm{LUC}}}}$. Shaded envelopes in (**a**) and (**c**–**i**) represent the mean ±1 SD. Exact cumulative values for each ${E}_{{{\rm{LUC}}}}$ component and scenario are reported in Supplementary Table 2.

When all LULCC cease immediately in Commit, global carbon removal peaks near −2 Pg C yr⁻^1^ by 2030 (Fig. 4a, b), reflecting fast forest regrowth and soil carbon recovery, then gradually stabilizes toward a near-zero balance by 2100 (Fig. 4c). Over 2024–2100, this scenario achieves a cumulative removal of −52 Pg C (Supplementary Table 4). BAU2030 delays land-use stabilization until 2030 (Fig. 4d), so it sustains emissions through mid-century before entering weak removals after 2050. The cumulative flux of 13 Pg C indicates that even modest delays in LULCC mitigation significantly erode the century-scale carbon benefit, as early emissions outweigh later recoveries. The Forest2100 scenario delivers the strongest mitigation (Fig. 4e), reaching −72 Pg C of cumulative removals by 2100. The total forest carbon store (${F}_{{{\rm{net}}}}$ = 204 Pg C) falls between 175 Pg C (referenced) and 278 Pg C (simulated) reported by Moustakis, Nützel^28^.

Both Gross2030 and Gross2050 scenarios become strong removals, following trajectories similar to Commit but with delayed peaks (Fig. 4f, g). Their cumulative removals reach −30 Pg C and −23 Pg C (Supplementary Table 2). This reflects that delaying the cessation of gross deforestation allows additional emissions to occur in the first decades. By contrast, the Net2030 and Net2050 scenarios permit continued gross deforestation balanced by afforestation, while following the same BAU2030 LULCC trajectory as the Gross scenarios for all non-targeted activities (Fig. 4h, i). The resulting cumulative fluxes are limited to 4 Pg C and 9 Pg C, respectively. These results indicate that policy stringency is the primary determinant of long-term land-use carbon benefits, while earlier implementation further enhances cumulative removals by shortening the period of avoidable emissions. Net-zero deforestation targets cannot replicate the mitigation potential of gross-zero strategies, even when implemented over shorter timescales.

In the Commit scenario (Fig. 4j), legacy deforestation emissions persist due to delayed carbon losses from soils and dead organic matter pools disturbed by historical deforestation, but they are more than offset by forest regrowth and substantial reductions in emissions from wood harvesting and shifting cultivation, which yield a strong net carbon removal by 2100 (Supplementary Table 2). Under BAU2030, continued land conversion until 2030 leads to a nearly neutral land-cover-change balance, while additional emissions from shifting cultivation and wood harvesting result in a modest cumulative carbon source. The persistence of agricultural expansion and logging prevents large-scale recovery even after LULCC has stabilized. In Forest2100, large-scale forest expansion dominates the global carbon balance, producing the strongest cumulative removals despite continued deforestation and small emissions from harvest and shifting cultivation. The Gross2030 scenario substantially reduces deforestation-related emissions and enhances net removals, while delaying gross-zero deforestation to 2050 weakens the mitigation effect. In contrast, the Net2030 and Net2050 scenarios deliver only limited net removals, as continued shifting cultivation and wood harvesting constrain the overall mitigation potential (Supplementary Table 2).

**Fig. 5:**
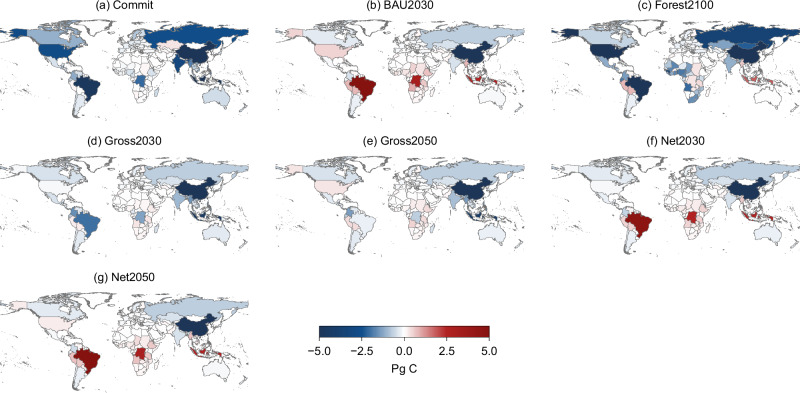
Regional cumulative land-use and land-cover change emissions (*E*_LUC_) emissions from 2024–2100 under idealized land-use and land-cover change (LULCC) scenarios driven by low-warming climate and atmospheric CO₂ forcing. Maps show regional cumulative emissions at 2100 for: **a** Commit (immediate cessation of all LULCC from 2023), **b** BAU2030 (Business As Usual: baseline continuation of current land-use trends to 2030), **c** Forest2100 (intense afforestation to a constrained global maximum forest area by 2100), **d** Gross2030 (all gross deforestation halted by 2030), **e** Gross2050 (all gross deforestation halted by 2050), f Net2030 (net deforestation halted by 2030), **g** Net2050 (net deforestation halted by 2050). The shared color bar indicates net cumulative emissions. Blue tones denote net regional carbon uptake (negative emissions) and red tones denote net regional carbon release (positive emissions), with white near zero. Map boundaries are sourced from the Resource and Environmental Science Data Platform (https://www.resdc.cn).

At the regional scale, the Commit scenario results in widespread carbon removals, with particularly strong cumulative removals in the major forested and regrowing regions (Fig. 5a, Supplementary Table 3). Under BAU2030, pronounced contrasts persist between tropical source regions dominated by deforestation and temperate or boreal sink regions, broadly reflecting historical land-use patterns (Fig. 5b). The Forest2100 scenario produces the strongest regional removals overall, although some tropical regions remain weak carbon sources despite extensive afforestation (Fig. 5c). Gross2030 leads to marked reversals across many tropical regions, transforming major historical carbon sources into sinks, while Gross2050 exhibits similar but weaker patterns (Fig. 5d, e). In the Net2030 and Net2050 scenarios, several temperate and boreal regions continue to act as sinks, but persistent tropical deforestation causes many low-latitude regions to remain net carbon sources, dominating the global spatial pattern of ${E}_{{{\rm{LUC}}}}$ (Fig. 5f, g, Supplementary Table 3).

## Discussion

This study quantifies future LULCC carbon fluxes with a suite of shifted SSP and idealized scenarios. The results discussed below primarily focus on ${E}_{{{\rm{LUC}}}}$ under a fixed low-warming climate pathway (see “Methods” and Supplementary Tables 2–4).

Under shifted SSP scenarios, low-emission pathways such as SSP1-1.9 and SSP1-2.6 achieve global carbon removals, driven by early cooperation and strict deforestation control. In contrast, high- and intermediate-emission pathways (SSP3–7.0, SSP4–6.0, SSP5–8.5) remain carbon sources through 2100, with cumulative emissions of 30–90 Pg C, reflecting weak governance and continued tropical forest losses^26,27^. The cumulative magnitudes of deforestation emissions and forest regrowth removals substantially exceed those of other land-use components (Figs. 2, 4), indicating that these two processes dominate the global land-use carbon balance, which is consistent with previous assessments^29^. Secondary differences arise from wood harvest and cultivation shifts. Regional contrasts reveal the asymmetry in land carbon fluxes. China and Indonesia act as persistent carbon sinks across all SSPs, reflecting the effectiveness of sustained afforestation, reforestation, and land restoration programs^30,31^. In contrast, Brazil and COD remain dominant emission sources under all but the most ambitious sustainability pathways, confirming that tropical forest protection remains central to achieving global net-zero land carbon fluxes^29,32^.

Idealized scenarios provide further insight into the mechanisms and temporal sensitivities underlying these patterns. Scenarios halting forest change early, such as Commit and Gross2030, induce cumulative sequestration, reaching −52 Pg C and −29 Pg C by 2100. The Forest2100 scenario produces the strongest removals (−72 Pg C), confirming that large-scale and continuous forest expansion can drive durable carbon sinks^29^. However, continued deforestation, wood harvest, and restricted land availability constrain additional afforestation potential in the tropics. Given the slow carbon sequestration rates of young forests, the carbon sinks from afforestation are unlikely to offset the carbon emissions produced by tropical deforestation and wood harvest in the short to medium term^33,34^. In contrast, temperate and boreal regions, such as China, Russia, and North America, will display consistent carbon removals throughout the century, driven by abundant forest resources and lower land-use turnover^35^. While tropical regions offer the largest opportunity for reducing emissions, temperate regions contribute most to sustained carbon sequestration^36^. Meanwhile, delaying the cessation of deforestation from 2030–2050 (Gross2030 and Gross2050) reduces achievable removals by nearly 40%, demonstrating the importance of policy timing. By contrast, Net2030 and Net2050 achieve only weak removals, as ongoing deforestation continues to emit carbon faster than newly planted forests can sequester it^36^. The timing and ambition of land governance determine whether the land system functions as a carbon source or a sink^26,37,38^. Although regrowth following wood harvest and cultivation shifts can partially compensate legacy losses^39^, persistent deforestation remains the largest obstacle to achieving removals. Patterns in each scenario highlight early, coordinated, and ambitious land-use governance, particularly in tropical regions, as the most immediate levers for climate mitigation. Conversely, fragmented or delayed policy implementation risks decades of irreversible carbon losses.

Uncertainties remain in quantifying the magnitude and persistence of land-based carbon. This version of OSCAR shows a stronger-than-average CO₂-fertilization effect when compared to CMIP6 models^40^ and, consequently, a lower-than-average sensitivity to climate change, although this only marginally affects ${E}_{{{\rm{LUC}}}}$. Similarly, carbon density parameters and forestry management assumptions introduce regional uncertainties, particularly regarding soil carbon and harvested wood products^41,42^. The optimistic afforestation potential from Moustakis, Nützel^28^ used here should be interpreted as the globally ambitious afforestation effort inferred from the Sixth Assessment Report (AR6) Scenarios Database; realistic outcomes will depend on biophysical, socio-economic, and ecological constraints that may reduce available land and sequestration efficiency^7,43^. Total land carbon fluxes (${F}_{{{\rm{net}}}}$) reflect the combined effects of LULCC on ${E}_{{{\rm{LUC}}}}$ and of atmospheric CO₂ and climate change on the ${S}_{{{\rm{land}}}}$. While the latter dominates the magnitude of total flux changes, it is largely externally driven and therefore does not reflect mitigation from land-use policy. ${E}_{{{\rm{LUC}}}}$ remains the appropriate metric to assess the impact of land-use interventions. This study assumes the impacts of LULCC on land carbon fluxes are independent of the biogeophysical effects of LULCC on climate. In reality, interactions between LULCC and climate influence the natural land sink, as LULCC modifies surface radiation, energy and water balances, and regional meteorology, thereby altering temperature, moisture availability, fire activity, and atmospheric CO₂^1^. The comparison between low-warming and high-warming scenarios indicates that this does not affect the main conclusions of this study, as LULCC-driven carbon fluxes are dominated by LULCC rather than by climate.

SSP LULCC trajectories are temporally shifted to ensure continuity with the observation-constrained historical period, which preserves their internal dynamics but breaks full consistency with other SSP dimensions (e.g., population or non-CO₂ emissions). These scenarios should therefore be interpreted as stylized post-historical land-use pathways rather than calendar-year-specific policy projections. A key constraint in designing idealized scenarios is that indefinitely extrapolating recent land-use trends or holding present-day land-use change rates constant over multiple decades leads to non-physical outcomes in many regions, including negative land areas, land-use transitions exceeding national land availability, or highly unrealistic land-use compositions. The shifted SSP scenarios, therefore, combine near-term continuation of recent trends with long-term constraints to ensure physical plausibility.

This study is accompanied by an open dataset of future carbon emissions from land-use and land-cover change (10.5281/zenodo.16729725), extending OSCAR-based Global Carbon Budget estimates to 2100. The dataset provides national-level projections under two climate pathways and five climate models, including eight SSP-based and seven idealized scenarios. These 150 realizations allow users to mix and match scenarios by country or component, enabling the construction of customized global or regional land-use futures. This flexible structure offers a unique opportunity to assess how alternative national land-use policies will shape global carbon outcomes.

## Methods

### The OSCAR model

OSCAR is a reduced-complexity Earth system model designed to emulate the behavior of state-of-the-art process-based models while remaining computationally efficient^44^. OSCAR simulates transient responses of net primary productivity, wildfires, heterotrophic respiration, and other processes to changes in atmospheric CO₂ and climate, and incorporates a bookkeeping module to track carbon fluxes following LULCC^44,45^. The bookkeeping framework distinguishes LCC and LU (wood harvest and shifting cultivation), enabling attribution of emissions to specific land-use activities and regions^23^. Here, we use OSCAR v3.3 and run offline simulations with its land carbon cycle module only, following the setup used for the GCB^23^ (Supplementary Fig. 1).

### Flux definitions

The OSCAR model outputs for the terrestrial ecosystem carbon fluxes are provided annually at regional scales. A list of all variables and their subcomponents’ information is given in Supplementary Table 1. The three main fluxes are ${E}_{{{{\rm{LUC}}}}}$, ${S}_{{{{\rm{land}}}}}$ and ${F}_{{{{\rm{LASC}}}}}$, the sum of which represents the net land-to-atmosphere CO₂ flux (${F}_{{{{\rm{ne}}}}t}$):

1 $${F}_{{{{\rm{net}}}}}={E}_{{{{\rm{LUC}}}}}+{S}_{{{{\rm{land}}}}}+{F}_{{{{\rm{LASC}}}}}$$

Note that the definition of ${S}_{{{{\rm{land}}}}}$ in the GCB^4^, that is, the natural land sink under preindustrial land cover, differs from the usual definition used with OSCAR^23^. To obtain the natural land sink under evolving land cover (${S}_{{{{\rm{land}}}},{{{\rm{actual}}}}}$), one must sum ${S}_{{{{\rm{land}}}},{{{\rm{PI}}}}}$ and ${F}_{{{{\rm{LASC}}}}}$:

2 $${{{{\rm{S}}}}}_{{{{\rm{land}}}},{{{\rm{actual}}}}}={S}_{{{{\rm{land}}}},{{{\rm{PI}}}}}+{F}_{{{{\rm{LASC}}}}}$$

where ${S}_{{{{\rm{land}}}},{{{\rm{PI}}}}}$ represents the natural land sink under preindustrial land cover and ${F}_{{{{\rm{LASC}}}}}$ refers to the loss of additional sink capacity (LASC) due to land use changes. OSCAR characterizes ${{{{\rm{F}}}}}_{{{{\rm{LASC}}}}}$ as the difference between the carbon uptake capacity of natural vegetation under preindustrial land cover and that under actual evolving land cover^2^. This formulation of LASC corresponds to what has recently been termed Replaced Sources and Sinks (RSS) in the GCB-related paper^46^. By contrast, Dorgeist, Schwingshackl^5^ define LASC as a residual (i.e., synergistic) term within a factorial experimental design, which aggregates the loss of sink capacity with an interaction component that is treated separately as part of ${{{{\rm{E}}}}}_{{{{\rm{L}}}}{{{\rm{U}}}}{{{\rm{C}}}}}$ in our framework.

The flux ${E}_{{{{\rm{LUC}}}}}$ is broken down into component fluxes. Two high-level components are the fluxes from LCC, ${E}_{{{{\rm{LCC}}}}}$, and from LU, ${E}_{{{{\rm{LU}}}}}$:

3 $${E}_{{{{\rm{LUC}}}}}={E}_{{{{\rm{LCC}}}}}+{{{{\rm{E}}}}}_{{{{\rm{LU}}}}}$$

where negative values of ${E}_{{{{\rm{LUC}}}}}$ correspond to carbon removals from the atmosphere (${E}_{{{{\rm{LUC}}}}}$ < 0), whereas positive values indicate carbon emissions associated with LULCC (${E}_{{{{\rm{LUC}}}}}$ > 0).

${E}_{{{{\rm{LU}}}}}$ is further broken down into four fluxes:

4 $${E}_{{{{\rm{LU}}}}}={{{{\rm{E}}}}}_{{{{\rm{harvest}}}}}+{S}_{{{{\rm{harvest}}}}}+{E}_{{{{\rm{shifting}}}}}+{S}_{{{{\rm{shifting}}}}}$$

Here, ${E}_{{{{\rm{harvest}}}}}$ and ${S}_{{{{\rm{harvest}}}}}$ are related to wood harvest (the emission from harvested wood products (HWP), and the corresponding biospheric regrowth) and the other two to shifting cultivation (rotation cycle split between loss and gain of natural land).

We offer two possible (but incompatible) breakdowns of ${{{{\rm{E}}}}}_{{{{\rm{LCC}}}}}$ that could be of interest to users. Both decompositions sum to the same total ${E}_{{{{\rm{LCC}}}}}$ and differ only in the attribution of subcomponents. One focused on forest, whereby we isolate the flux from deforestation (${E}_{{{{\rm{deforest}}}}}$) and the one from afforestation/reforestation (${S}_{{{{\rm{reforest}}}}}$), grouping all other LCC transitions as the remainder (${F}_{{{{\rm{others}}}}}$):

5 $${E}_{{{{\rm{LCC}}}}}={E}_{{{{\rm{deforest}}}}}+{S}_{{{{\rm{reforest}}}}}+{F}_{{{{\rm{others}}}}}$$

Another one focused on anthropogenic biomes, which is not used in the results presented here but is documented for completeness and for users interested in agricultural land-use dynamics. We isolate the fluxes induced by the establishment or abandonment of cropland or pasture, as well as by other LCC between natural land types or anthropogenic land types, and a different remainder:

6 $${E}_{{{{\rm{LCC}}}}}={E}_{{{{\rm{cropland}}}}}+{E}_{{{{\rm{pasture}}}}}+{S}_{{{{\rm{cropland}}}}}+{S}_{{{{\rm{pasture}}}}}+{F}_{{{{\rm{natLCC}}}}}+{F}_{{{{\rm{antLCC}}}}}+{F}_{{{{\rm{rest}}}}}$$

Finally, we also isolate two subcomponent fluxes related to the forest biome that cannot be reconstructed using the other provided fluxes. The first one (${F}_{{{{\rm{forest}}}}}={E}_{{{{\rm{LUC}}}}}^{{{{\rm{biome}}}}={{{\rm{forest}}}}}$) is the bookkeeping flux occurring in forest but excluding shifting cultivation; it is a part of ${E}_{{{{\rm{LUC}}}}}$ related to forests. The second one (${S}_{{{{\rm{forest}}}}}={S}_{{{{\rm{land}}}},{{{\rm{PI}}}}}^{{{{\rm{biome}}}}={{{\rm{forest}}}}}$) is the natural land sink occurring in forest; it is a part of ${S}_{{{{\rm{land}}}},{{{\rm{PI}}}}}$ related to forests. The sum of both gives the carbon flux over forested land, excluding shifting cultivation. Importantly, the first of these two fluxes is used to define conventional carbon dioxide removal (CDR) on forest land by Gidden, Gasser^22^ and in the State of CDR reports^47,48^, while the second one can be used to approximate the LULCC emissions as defined by national inventories (${E}_{{{{\rm{NGHGI}}}}}$), following the adjustment suggested by Grassi, Stehfest^14^ and subsequent work with OSCAR^22^:

7 $${E}_{{{{\rm{NGHGI}}}}}={E}_{{{{\rm{LUC}}}}}+{f}_{{{{\rm{managed}}}}}\cdot {S}_{{{{\rm{forest}}}}}$$

where ${f}_{{{{\rm{managed}}}}}$ is the fraction of forested area considered managed in a given region. Note that we do not provide this final variable, as it is neither an input nor an output of the OSCAR model.

### Historical forcings

In offline land-only mode, OSCAR requires inputs of atmospheric CO₂, regional climate, and regional LULCC. Atmospheric CO₂ concentration comes from the GCB2024, provided to TRENDY models as a best-guess annual and global time series covering 1701 to 2023. The climate inputs of temperature and precipitation from 1901 to 2023 are from the latest (currently, v4.08) CRU-TS dataset^49^, consistent with the TRENDY protocol. Prior to 1901, the 1901–1920 average is used as a preindustrial baseline. LULCC inputs are provided by two alternative datasets: the latest iteration of the LUH2 dataset was made specifically for each GCB^4,24^, and the Houghton and Castanho^50^ dataset derived from FAO Forest Resource Assessments. It is notably derived from the FRA, which is released every five years. The latest version is based on FRA2020^51^ and therefore stops in 2020. The regional trends in the LULCC forcings over the last five years of data are used to linearly extrapolate this dataset to the year 2023.

### Ensemble simulations and uncertainty assessment

The OSCAR model integrates uncertainty through a Monte Carlo ensemble approach^15,23,44^. We generate 2000 parameter sets describing biogeochemical processes, which result in 4000 ensemble members once combined with the two LULCC input datasets. The unconstrained ensemble has been shown to display unreasonably large uncertainty in ${S}_{{{{\rm{land}}}}}$^23^, which is further constrained using an ensemble-weighting approach. Based on the distance between the simulated and the constraining (i.e., assessed) values of a variable *x*, we assign a weight (*w*) to each ensemble member. This weight is calculated as the following equation assuming a Gaussian distribution of the posterior uncertainty:

8 $$w\left(x\right)=\frac{1}{\sigma \sqrt{2\pi }}exp \left(-\frac{{\left(x-\mu \right)}^{2}}{2{\sigma }^{2}}\right)$$

where μ is the average of the constraint, σ is the corresponding standard deviation, and x is the value of each simulation of the ensemble. The constraint is the estimate of ${S}_{{{{\rm{land}}}},{{{\rm{actual}}}}}$ over the instrumental observation period (from 1960 onwards), provided by the GCB2023, which is currently 150 ± 35 Pg C over 1960–2022^16^. The ensemble is evaluated over the same period to ensure consistency between the simulated and observationally constrained quantities. Here, we note that while we have demonstrated the strength of using regional constraints to better match national inventories^15^, this is not yet implemented in this standard GCB setup.

The weights are then used to calculate the weighted average and weighted standard deviation of the simulation results. Note that the values for OSCAR reported in the GCB are the weighted average only. However, since our goal is to extend these historical simulations to future periods, we run the same 4000 ensemble members for every single scenario (a total of 150) in this work and provide weighted outputs, following the standard OSCAR configuration used in the GCB.

### Historical initialization

A key feature of the dataset we propose is that it extends the historical simulation made with OSCAR to contribute to the latest GCB^4^. GCB2024 provides estimates of global carbon-cycle fluxes up to 2023, implying that our scenarios start in 2024. Since ${E}_{{{{\rm{LUC}}}}}$ of a given year depends on the LULCC of previous years (the so-called legacy effect), we initialize OSCAR with prescribed historical land-use transitions and corresponding carbon pool states, consistent with the standard OSCAR configuration used for the GCB2024^23^.

### CO₂ and climate scenarios

Future atmospheric CO₂ and climate forcings are taken from the ISIMIP 3b^52^. The ISIMIP project provides a consistent and bias-corrected^53^ projection of global climate patterns under part of the SSP scenarios^54^. We choose the SSP1-2.6 and SSP3-7.0 scenarios to represent low- and high-warming futures, respectively, given that SSP5-8.5 can be deemed unrealistic^55^. We derive surface air temperature and precipitation projections from five climate models (GFDL-ESM4, IPSL-CM6A-LR, MPI-ESM1-2-HR, MRI-ESM2-0, and UKESM1-0-LL), as well as the atmospheric CO₂ concentration of these scenarios^56^.

To facilitate a smooth transition between projection data and the latest GCB historical data, we apply an additive regional adjustment factor (i.e., an offset) to the ISIMIP data:

9 $$X\left(t\right)={X}_{{{{\rm{ISIMIP}}}}}\left(t\right)+{X}_{{{{\rm{GCB}}}}}^{*}-{X}_{{{{\rm{ISIMIP}}}}}^{*}$$

where *X* is the input variable for OSCAR, the subscript denotes the data source, and the star superscript denotes the average value taken over a reference period. For climate variables, this correction is region-specific and done with the average of the last 51 years (1973-2023); for atmospheric CO₂, it is global and based on the 2023 value.

### LULCC scenarios

The LULCC scenarios consist of eight shifted SSP scenarios and seven idealized ones based on historical trends, all run orthogonally to the five climate models under high- and low-warming scenarios, leading to a total of 150 possible futures. LULCC is decomposed into three components: land cover change (LCC), shifting cultivation, and wood harvest, which are illustrated in Supplementary Fig. 2. Here, the effects of LU and LCC on land carbon fluxes are simulated independently from the biogeophysical impacts of land-use change on climate, as this study focuses exclusively on the biogeochemical effects of LULCC. Climate forcing is prescribed from CO₂ and climate scenarios, allowing us to isolate the role of land-use policies and timing in shaping national LULCC emission pathways.

### Shifted SSP scenarios

The LULCC data for the eight SSP scenarios are taken from the LUH2 dataset for CMIP6^27^, which provides internally consistent socio-economic pathways derived from integrated assessment models (IAMs). The LUH2 data under SSP scenarios are provided from 2015 onwards, whereas our simulations must start in 2024, immediately after the end of the observation-constrained historical period used in the GCB2024. Advancing the historical endpoint to 2023 creates an inconsistency between the observed LULCC state and the initial years of the SSP projections, which are generated from an earlier historical baseline.

To address this mismatch, we apply a time shift to the LULCC data, such that the scenario-specific trajectories are initiated from the 2023 observational baseline. The first 77 years of LULCC data under each original SSP scenario (2015–2091) are reassigned to 2024–2100. Historical LCC transition data is simulated up to 2023, and the shifted SSP LCC transition data are then appended from 2024 onward. For LU components (wood harvest and shifting cultivation), an offset is applied using the mean of the last 11 years (2013–2023) as a reference, consistent with the treatment of CO₂ and climate forcings.

We acknowledge that this shifting breaks consistency with other aspects of the SSP scenarios (e.g., climate, CO₂ concentrations), but it is difficult to retain consistency of all aspects while changing the starting year of a scenario. The early phase of SSP scenarios typically exhibits the strongest divergence in LULCC trajectories, reflecting differences in socio-economic pathways, whereas later periods tend to show more gradual and convergent changes. Our objective is therefore to apply the short-term dynamics and narrative divergence embedded in SSP LULCC pathways from an updated historical endpoint, although relaxing their association with specific calendar years. This approach provides a pragmatic compromise that maintains scenario-specific land-use narratives while ensuring continuity with observation-constrained historical conditions.

### Idealized scenarios

Using a relatively simple design, we define seven idealized alternative LULCC scenarios relevant for scientific and policy implications (Supplementary Fig. 2). The last three sets of scenarios (with names starting with Forest, Gross, and Net) are designed to explore the potential influences of different forest-related policies on the terrestrial carbon cycle. These scenarios are intended as counterfactual experiments rather than predictions of the most likely future. They are constructed to remain physically consistent with land availability and land-use competition over the 21st century. Total forest areas in all scenarios are displayed in Supplementary Table 4.

#### Committed emissions (Commit)

The Commit scenario assumes an immediate cessation of all LULCC activities, resulting in all LCC and LU input data dropping to zero as of year 2023 inclusive. While unrealistic, this scenario is designed as a diagnostic experiment to isolate the legacy carbon dynamics arising from historical LULCC perturbations, under the two assumed climate scenarios (low- and high-warming).

#### Business As usual (BAU2030)

The Business As Usual (BAU2030) scenario represents a constrained continuation of current land-use dynamics in the absence of additional policy intervention, serving as the baseline for all idealized scenarios. All LULCC in each region from 2023 to 2030 are linearly extrapolated from the trends of the past 11 years (currently, 2013-2023). After that, LCC is assumed to linearly reach zero by 2050, and LU is held constant at 2030 levels. This transition is introduced to prevent non-physical outcomes over long time horizons, such as land-use changes exceeding available land area or unrealistic dominance of a single land-use type by 2100. The choice of 2030 as the transition year reflects its central role in the COP26 commitment to halt and reverse forest loss by 2030.

#### Intense afforestation (Forest2100)

The Forest2100 scenario assesses the potential carbon sinks that could result from ambitious afforestation policies. In this scenario, intensified afforestation on pasture land aims to reach a regionally constrained global maximum forest area (935 Mha) in 2100, following Moustakis, Nützel^28^. All other LULCC activities follow the BAU2030 scenario. In each region, we calculated the forest area change that occurred between 2015 and 2023, as well as the projected forest area change associated with other land-use change activities under the BAU2030 scenario over the period 2024–2100. These quantities were subtracted from the regional potential forest area; the remaining area represents the forest area increase required in each region over 2023–2100. This additional forest area is then allocated over time by assuming a linear increase in annual afforestation rates from 2024 to 2030, followed by a constant annual afforestation rate thereafter.

#### Stopping gross deforestation (Gross2030, Gross2050)

This set of scenarios is motivated by the Glasgow declaration on forests, which aimed to halt the reduction of global forest area by 2030 but did not specify a clear pathway for achieving this target, which allows two very distinct possible interpretations^57^. One approach to ensuring forest area stability is to halt all forest loss-inducing activities, while another approach involves balancing the rate of forest loss with the rate of forest gain. In the Gross2030 scenario, all forest-related LCCs reach zero by 2030. Shifting cultivation is phased out together with deforestation, while wood harvest follows the BAU2030 trajectory, since shifting cultivation involves burning young forests. All other LULCC activities follow the BAU2030 scenario. Recognizing the challenge of achieving this target by 2030, we also consider that the goal may be delayed to be reached by 2050, thereby defining the Gross2050 scenario.

#### Stopping net deforestation (Net2030, Net2050)

The Net2030 scenario is the other interpretation of the Glasgow declaration on forest^57^, whereby we assume that the deforestation rate will decrease to match the afforestation rate of the BAU2030 by 2030, leading to forest area stability after that, while other LULCC activities, including shifting cultivation and wood harvest, follow the BAU2030 scenario. Afforestation in the Net scenarios follows the BAU2030 trajectory, which is extrapolated from recent trends. As previously, the Net2050 scenario adopts a similar trajectory, but the target year is set to 2050 instead.

## Supplementary information

Supplementary Information

Transparent Peer Review file

## Data Availability

The land carbon flux data generated in this study have been deposited in Zenodo (10.5281/zenodo.16729725)^58^ and serve as source data for all main and Supplementary Figs. in this paper. The atmospheric CO₂ concentration data used in this study are available from GCB2024 (10.18160/GCP-2024)^4^. The climate data used in this study are available from CRU-TS (http://catalogue.ceda.ac.uk/uuid/3f8944800cc48e1cbc29a5ee12d8542d). The LULCC data used in this study are available from LUH2 (https://zenodo.org/records/6570919) and FAO FRA (https://www.fao.org/faostat/en/#data). The projected atmospheric CO₂ concentration data are available from CMIP6 (https://wcrp-cmip.org/cmip-data-access/), projected climate data from ISIMIP, and projected LULCC data from LUH2 (https://zenodo.org/records/6570919).

## Code availability

The source code of OSCAR v3.3 is available at https://github.com/tgasser/OSCAR^59^. Scripts required to reproduce the figures and tables in this paper are available at 10.5281/zenodo.16729725^58^.
