# Supplementary material for: National pathways of land-use CO₂ emissions in the 21st century
Source: Nat Commun. 2026 Jun 24;17:7902. doi: 10.1038/s41467-026-74836-w (PMC13443099; doi:10.1038/s41467-026-74836-w)
Supplement: Supplementary file 1 [file 41467_2026_74836_MOESM1_ESM.pdf]

National pathways of land-use CO_2 emissions in the 21st century

Danni Zhang[1,2,3], Bo Zheng[1,3], Yue He[4], Tianyi Wang[1,3], Gaurav P. Shrivastav[2], Philippe Ciais[5], Thomas Gasser[2,5]

[1] Shenzhen Key Laboratory of Ecological Remediation and Carbon Sequestration, Institute of Environment and Ecology, Tsinghua Shenzhen International Graduate School, Tsinghua University, Shenzhen, 518055, China.
[2] International Institute for Applied Systems Analysis, 2361 Laxenburg, Austria.
[3] State Environmental Protection Key Laboratory of Sources and Control of Air Pollution Complex, Beijing, 100084, China.
[4] Institute of Carbon Neutrality, Peking University, Beijing, 100871, China
[5] Laboratoire des Sciences du Climat et de l'Environnement, CEA–CNRS–UVSQ, IPSL, Université Paris-Saclay, 91191 Gif-sur-Yvette, France.

Correspondence to: Bo Zheng (bozheng@sz.tsinghua.edu.cn); Thomas Gasser (gasser@iiasa.ac.at)

Supplementary Figures

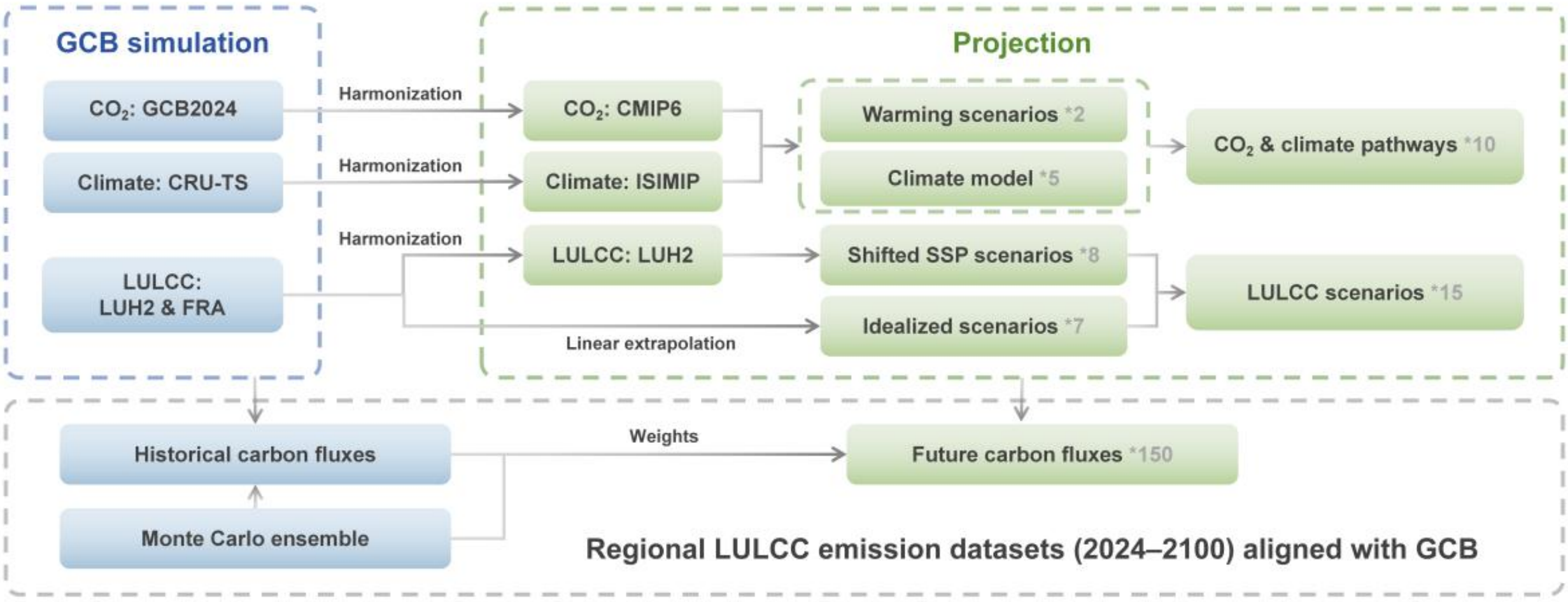

Supplementary Fig. 1. Methodological flowchart outlining the projection of LULCC emissions. The historical forcing data include atmospheric CO_2 concentrations from the Global Carbon Budget 2024 (GCB2024) assessment, climate data from the Climatic Research Unit Time Series (CRU-TS), and LULCC data from the Land-Use Harmonization 2 (LUH2) project and the Food and Agriculture Organization's Forest Resource Assessment (FRA). The driving data used for projections include atmospheric CO_2 concentrations from CMIP6, climate change data from the Inter-Sectoral Impact Model Intercomparison Project (ISIMIP), and projected LULCC data provided by LUH2.

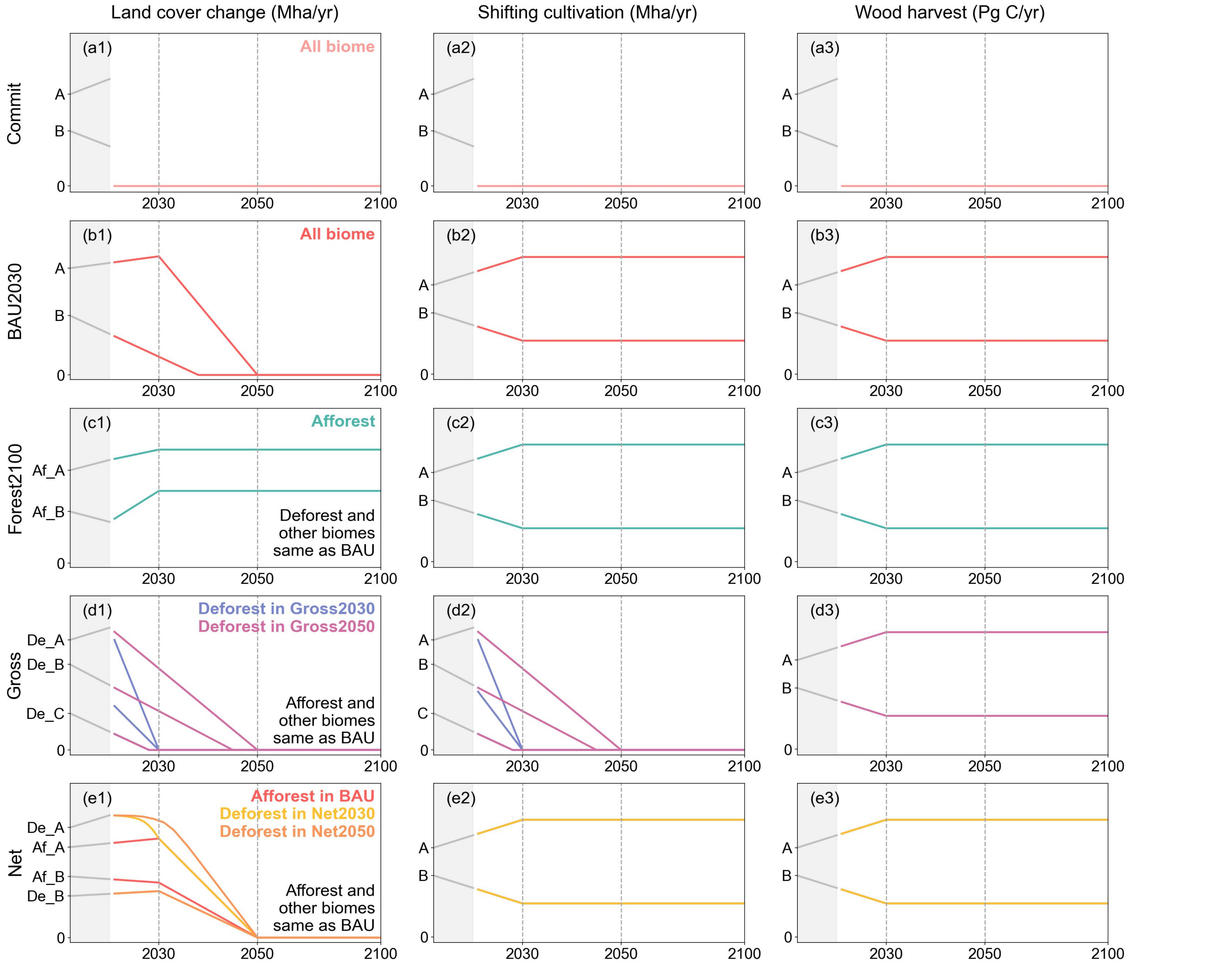

Supplementary Fig. 2. Design of the idealized LULCC scenarios. LULCC scenarios include the Committed Emissions scenario (a), Business-as-Usual scenario (b), Intense Afforestation scenario (c), Stopping Gross Deforestation scenario (d), and Stopping Net Deforestation scenario (e). The first column presents land cover change (LCC), the second column displays shifting cultivation, and the third column shows wood harvest. In each panel, the gray line represents historical data from 2000 to 2023. Regions A, B, and C correspond to areas with different historical trends. In panels (a), (b), (c2), (c3), (d2), (d3), (e2), and (e3), data for all biomes are extrapolated to 2100 following their respective scenario trajectories. Under the Intense Afforestation scenario, only the afforestation area is extrapolated based on the trajectory shown in panel (c1), while other LCC data follow the Business-as-Usual scenario. Af_A represents regions where the afforestation area showed an increasing trend during the historical period, and Af_B represents regions with a decreasing trend. The area enclosed by the green line and the x-axis indicates the target afforestation area in those regions. In the Stopping Gross Deforestation and Stopping Net Deforestation scenarios, deforestation data are extrapolated based on the trajectories in panels (d1) and (e1), respectively. Other LCC data remains consistent with the Business-as-Usual scenario. In panel (d1), De_A represents regions with historically increasing deforestation area, De_B shows a decreasing trend, and De_C indicates areas where deforestation decreased and dropped to zero before 2030. In panel (e1), De_A and Af_A represent deforestation and afforestation areas in Region A, respectively, and De_B and Af_B represent the same for Region B. Before 2030, deforestation area exceeds afforestation area in Region A, while in Region B, afforestation area is greater than deforestation.

Supplementary Tables

Supplementary Table 1. Carbon fluxes definitions.

Fluxes	Definitions
Main fluxes	
E_{LUC}	Net bookkeeping LULCC emissions
$S_{\text{land, actual}}$	The natural land sink under evolving land cover
$S_{\text{land, PI}}$	Natural land sink under preindustrial LC
F_{LASC}	Loss of natural land sink caused by LCC (add to land sink under preindustrial LC to get actual land sink)
F_{net}	Net carbon exchange from land to atmosphere ($E_{\text{LUC}} + S_{\text{land, actual}}$)
Components of E_{LUC}	
E_{deforest}	Emissions from deforestation (all LCC from forest but not to forest)
S_{reforest}	Removals from afforestation/reforestation (all LCC to forest but not from forest)
E_{harvest}	Emissions from wood harvest (HWP oxidation caused by harvesting LU, or by LCC from one type of natural land to the same type)
S_{harvest}	Removals from wood harvest (biospheric regrowth from the same causes)
E_{shifting}	Emissions from shifting cultivation (LU; part of the cycle involving loss of natural land)
S_{shifting}	Removals from shifting cultivation (LU; part of the cycle involving gain of natural land)
F_{others}	Flux from all other LU and LCC
F_{harvest}	$E_{\text{harvest}}+S_{\text{harvest}}$
F_{shifting}	$E_{\text{shifting}} + S_{\text{shifting}}$
E_{LUC}	$E_{\text{deforest}} + S_{\text{reforest}} + E_{\text{harvest}}+S_{\text{harvest}}+E_{\text{shifting}} + S_{\text{shifting}} + F_{\text{others}}$
Alternative breakdown	
E_{cropland}	Emissions from cropland establishment (LCC from natural land to cropland)
E_{pasture}	Emissions from pasture establishment (LCC from natural land to pasture)
S_{cropland}	Removals from abandonment of cropland (LCC from cropland to natural land)
S_{pasture}	Removals from abandonment of pasture (LCC from pasture to natural land)
F_{natlcc}	Flux from LCC between natural land types (LCC from one type of natural land to a different type)
F_{antlcc}	Flux from LCC between anthropogenic land types (LCC from one type of anthropogenic land to another type)
F_{rest}	Remainder flux in the alternative breakdown
E_{LUC}	$E_{\text{cropland}} + E_{\text{pasture}} + S_{\text{cropland}} + S_{\text{pasture}}+E_{\text{harvest}} + S_{\text{harvest}} + E_{\text{shifting}} + S_{\text{shifting}} + F_{\text{natLCC}} + F_{\text{antLCC}} + F_{\text{rest}}$
Forest-specific fluxes	
F_{forest}	Net bookkeeping flux occurring in forest excluding shifting cultivation
S_{forest}	Actual natural land sink occurring in forest

Supplementary Table 2. Cumulative contributions of E_{LUC} components across LULCC scenarios under low warming in 2024-2100 (Unit: Pg C).

Scenario	E_{deforest}	S_{reforest}	F_{harvest}	F_{shifting}	F_{others}	E_{LUC}
SSP1-1.9	36.7	-44.1	-3.4	-4.4	3.4	-12.0
SSP1-2.6	36.9	-44.2	-3.5	-4.6	1.7	-13.6
SSP2-4.5	40.9	-40.8	2.8	-3.9	10.0	9.0
SSP3-7.0	77.0	-32.1	1.9	-5.0	15.0	56.7
SSP4-3.4	121.1	-50.4	6.6	-7.2	19.4	89.4
SSP4-6.0	64.3	-36.6	7.4	-6.4	13.0	41.7
SSP5-3.4-OS	61.5	-38.6	6.1	-4.0	18.5	43.4
SSP5-8.5	45.7	-26.9	4.8	-3.7	11.0	30.8
Commit	12.7	-18.2	-22.0	-23.6	-1.2	-52.3
BAU2030	25.5	-24.1	1.3	11.4	-0.8	13.2
Forest2100	25.5	-109.6	1.3	11.4	-0.7	-72.2
Gross2030	14.9	-24.1	1.2	-20.9	-0.8	-29.7
Gross2050	20.8	-24.1	1.2	-20.4	-0.8	-23.2
Net2030	15.9	-24.1	1.3	11.4	-0.8	3.7
Net2050	21.2	-24.1	1.3	11.4	-0.8	9.0

Supplementary Table 3. Cumulative regional contributions of E_{LUC} across LULCC scenarios under low warming in 2024-2100 (Unit: Pg C). Regions include China (CHN), Indonesia (IDN), Brazil (BRA), Democratic Republic of the Congo (COD), United States (USA), Russia (RUS), India (IND), Angola (AGO), Peru (PER), Tanzania (TZA), and Colombia (COL).

Scenario	CHN	IDN	BRA	COD	USA	RUS	IND	AGO	PER	TZA	COL
SSP1-1.9	-8.9	-3.8	3.8	1.4	-1.7	-1.5	-0.8	1.4	0.2	0.6	-0.5
SSP1-2.6	-8.6	-4.0	3.4	1.5	-1.6	-1.7	-0.8	0.5	0.2	0.7	-0.4
SSP2-4.5	-9.0	0.5	6.0	5.2	-1.5	-1.5	-0.2	0.9	0.4	0.8	-0.6
SSP3-7.0	-12.7	-1.5	6.8	19.6	4.3	-1.9	1.3	4.2	5.5	1.3	0.0
SSP4-3.4	0.1	12.7	-18.4	22.8	5.4	-3.3	2.1	6.5	0.4	2.1	-1.1
SSP4-6.0	-4.3	-3.1	-8.2	19.5	2.8	-1.3	1.3	4.9	0.3	1.9	-0.5
SSP5-3.4-OS	-4.7	-1.6	11.6	9.7	6.1	-1.5	0.8	1.2	0.3	3.1	0.0
SSP5-8.5	-5.3	-3.0	10.8	8.6	3.3	-0.7	0.8	0.7	0.3	3.6	-0.6
Commit	-7.2	-6.1	-4.8	-2.1	-2.7	-2.6	-3.2	0.0	0.1	0.0	-1.2
BAU2030	-6.3	2.6	8.1	3.1	0.5	-0.7	-0.4	0.9	0.8	0.6	-0.4
Forest2100	-11.1	1.8	-5.9	0.3	-7.2	-3.6	-1.1	-2.1	0.8	0.4	-1.4
Gross2030	-7.1	-4.4	-2.0	-1.3	-0.1	-0.7	-1.0	0.3	0.2	0.3	-1.6
Gross2050	-6.7	-4.2	-0.2	-0.7	0.3	-0.7	-0.9	0.5	0.4	0.4	-1.4
Net2030	-6.8	2.5	4.6	2.5	-0.1	-0.7	-0.4	0.5	0.5	0.5	-0.6
Net2050	-6.5	2.6	6.6	2.9	0.2	-0.7	-0.4	0.7	0.7	0.6	-0.5

Supplementary Table 4. Forest area (Mha) in 2023 and 2100 under different scenarios. Regions include global, Brazil (BRA), Canada (CAN), China (CHN), Democratic Republic of the Congo (COD), European Union (EU), Indonesia (IDN), Russia (RUA), and the United States of America (USA).

Scenario (year)	Global	BRA	CAN	CHN	COD	EU	IDN	RUS	USA
Historical (2023)	3914.2	471.0	445.6	227.6	135.1	149.3	102.1	833.2	303.9
SSP1-1.9 (2100)	3990.6	473.2	448.3	266.9	131.4	153.7	107.5	834.1	318.4
SSP1-2.6 (2100)	3967.1	468.9	447.8	261.2	131.4	151.5	107.2	834.5	311.0
SSP2-4.5 (2100)	3968.8	488.7	439.9	262.9	127.6	152.0	90.7	840.9	331.9
SSP3-7.0 (2100)	3593.1	445.0	430.9	287.2	13.9	145.4	96.4	840.3	266.0
SSP4-3.4 (2100)	3449.8	564.0	450.1	214.8	16.6	153.9	25.7	846.3	261.5
SSP4-6.0 (2100)	3765.4	534.1	451.4	248.6	32.6	153.0	106.9	852.1	295.1
SSP5-3.4-OS (2100)	3779.0	453.2	443.6	255.1	95.6	147.2	99.9	829.5	274.0
SSP5-8.5 (2100)	3789.6	451.3	444.5	241.9	84.0	146.4	114.3	834.5	282.7
BAU2030 (2100)	3842.7	435.6	446.5	242.8	125.6	152.9	100.2	833.6	298.0
Commit (2100)	3910.5	469.4	445.6	228.9	134.5	149.4	101.8	833.1	303.5
Forest2100 (2100)	4849.1	585.2	450.2	287.0	154.8	173.1	106.6	878.1	390.8
Gross2030 (2100)	3941.9	465.3	446.8	247.1	132.8	153.8	101.5	833.9	302.8
Gross2050 (2100)	3885.7	451.0	446.6	244.4	126.6	153.5	101.0	833.7	299.3
Net2030 (2100)	3933.8	464.0	446.5	245.6	132.8	153.2	101.6	833.8	302.6
Net2050 (2100)	3883.2	448.3	446.5	244.2	128.7	153.0	100.7	833.7	300.1
