# Supplementary material for: National pathways of land-use CO₂ emissions in the 21st century
Source: Nat Commun. 2026 Jun 24;17:7902. doi: 10.1038/s41467-026-74836-w (PMC13443099; doi:10.1038/s41467-026-74836-w)
Supplement: Supplementary file 2 [file 41467_2026_74836_MOESM2_ESM.pdf]

nature portfolio

Peer Review File

National pathways of land-use CO_2 emissions in the 21st century

Corresponding Author: Dr Bo Zheng

This file contains all reviewer reports in order by version, followed by all author rebuttals in order by version.

Version 0:

Reviewer comments:

Reviewer #1

(Remarks to the Author)
The authors leveraged the OSCAR model and a portfolio of LULCC and Climate scenarios to provide a set of national LULCC emission trajectories. The paper is well-written and the methodology is sound to me. I only have several questions:

- The author assumes the effects of land use change and climate change/variability on land carbon fluxes are independent, thereby isolating the relative contributions of land use-policies and climate forcing in driving emissions. I understand that this is a common approach in the carbon cycle research. However, as the well-know biogeophyscial effects of land use change in modulating climate (and thus carbon flux), it would be good to acknowledge this limitation and discuss this point.

- More detailed description of methods. For instance, what is S(land,PI) in Equation 2? How to derive F(LASC)?

- Validation against observations in history?

Minor comments:

- The model and data are publicly available, so do your Codes.

- Some references missing, such as Line 271, 275, etc.

- Add some tables for national-level statistics

Reviewer #2

(Remarks to the Author)
In the manuscript National pathways of land-use CO2 emissions in the 21st century, the authors use a set of SSP and idealized land use scenarios to quantify land use emissions and better understand their drivers. They find that there is a lot of variation in net carbon emissions, driven largely by the fate of forests. They also demonstrate that temperature change is a very small portion of the total variability, and land use decisions drive most variation. The authors also share the datasets they produce, which could be a helpful resource for future studies.

Overall I found the paper compelling but I have a number of comments about methodological clarity and methodological decisions that I think should be addressed before recommending the paper for publication.

Major comments

There were multiple elements of the methods that I found quite confusing, which need significant clarification.

1) The section about the shifted SSPs was not clear. L358 says something about “preserving land-use policy information that is typically more intense at the beginning of the scenarios” which was not well described. If the 2015-2024 period had a lot of the LU policy, wouldn’t we already be on one of the trajectories? Why would we assume that 2015 policies apply in

2025? I appreciate that the authors are clear that they are using shifted SSPs, but it's not clear to me from the text that this decision is preferable to a different harmonization.

2) I think the idealized scenarios need more justification. For example, although the BAU scenario is described in the methods, no description is provided for why these choices were made. A more obvious choice to me would have been to extrapolate out current trends indefinitely or keep existing levels constant. I don't understand why current trends continuing until 2030 and then changing is "business as usual". My comment on L254 is similar but for the Forest scenario.

3) The use of N and N-1 (and even N-2100) is pervasive throughout the methods and I found it very difficult to follow. Unless there is an explicit reason not to, please just state the years instead.

Line comments

57: Unclear what "second" is referring to

65: I found this description confusing. Why compare low warming? I thought the two scenarios are compared with high and low warming.

Fig 1b: The blue lines are too hard to see - especially the solid one. Also are both shadings shown or just the blue line? Can't tell as-is

98: After Fig 1 it's unclear what temperature is being used to drive results. I realize it doesn't make a big difference given the Fig 1 results, but it should be specified. As far as I can tell the Figure 5 caption is the only indication.

107: "Intermediate and high-emission scenarios…" is written as though it's a result, but I think it's an input

Fig 2: The colors look nice, but they are so similar that it's very tempting to read them all as SSPs. Especially for panel (b) the it's not obvious which legend the colors correspond to.

Fig 2 description: "To ensure temporal consistency, we adopted the IAMs-derived accumulative values for the period 2015-2092" This is a very confusing sentence. I think I might understand after a close read of the methods, but it makes little sense here.

Fig 3: Labels start at (c) and corresponding text references are off

156: Reference methods somewhere

164-165: This sentence feels out of context. It should either be removed or more thoroughly explained.
168: "showing the importance of early, stringent land-use regulation" Why does it show that? These numbers are only ~25% different, despite taking 3x longer to reach the goal

170-171: I like the point the authors are trying to make about offset-based policies, but I think the wording could use adjustment. Theoretically, offset policy could have afforestation increase past 2030. If I understand extended data figure 2e1 correctly, then afforestation is assumed to decline much more steeply after 2030 than current trajectories would suggest.

Fig 4: I generally like this figure and find it helpful, but all of the numbers in the text reference cumulative C. If the authors think cumulative carbon is the most relevant metric that should be compared between scenarios, it might make sense to make a figure that removes the time dimension - for example a stacked bar chart with experiment on the x and cumulative PgC on the y.

219-220: This reads like a result from this paper but I don't see this analysis and it cites a different paper

254: "upper bound" relates to my major comment. There are multiple countries (CAN, IDN) in Extended Figure 3 that have larger forest area than the afforestation scenario. Also Moustakis is referenced as the methodology, if I understood the methods correctly, alternations have been made to those forest areas.

277: F_LASC is not defined

307: "provided to TRENDY DGVMs as a best-guess annual…" there is no context for this statement. The acronym isn't defined and the purpose of this is not stated

324: sigma is the standard deviation of what?

326: Is the ensemble calculated over the same period? Is 30 the standard deviation?

335: Here N is 2024 and in the previous section N-1 was 2022.

338: "which follows a summary of the setup used with OSCAR for the GCB of year N" I don't know what this means

371: Broken links

Extended data Figure 3: The x axis is unclear. If the bands correspond to a year, why are they different widths? And why are the markers for the different lines in different locations on the x axis? Some of the colors are very difficult to tell apart. The use of markers helps, but the markers are not in the legend.

Reviewer #3

(Remarks to the Author)
Review of NCOMMS-25-90983-T:
National pathways of land-use CO2 emissions the 21st century

Summary

The authors estimate land use change emissions with a land model under several future scenarios to assess the potential impacts of different land change strategies on emissions. Two CMIP6 scenarios put these estimates in the context of other global assessments and several idealized scenarios provide insights to the potential effects of particular approaches. They show that halting deforestation is the most effective approach for reducing emissions, with additional afforestation providing further benefits in the form of carbon sequestration.
They also provide these emission data across all their scenarios at the country level for others to use.

Overall response

This is an interesting and useful paper both in terms of its results and its demonstration of how a land model can track these land fluxes in a detailed manner. I have only one major reservation with the method, and some suggestions for improving clarity.

1) The application of SSP LULCC forcings appears to create a major discontinuity at the beginning of 2024 (due to 'shifting' the scenarios). This could be because it isn't explained adequately, but conceptually it does not seem to align withe the extrapolation that was used to extend the historical simulation through 2023.

2) Some things need more explanation in the results/discussion if the methods section will be at the end of the paper. Otherwise the paper is difficult to comprehend if the reader has not read the methods section first. For example, referring to the extended data table and figures when introducing the corresponding info would be helpful.

3) There are some grammatical errors scattered throughout the paper that should be addressed.

Specific comments and suggestions

Abstract

Lines 16-17
It is a little confusing to refer to the same carbon flux process as 'removals' and 'gains, and also have one number negative and the other positive. Please make these two consistent.

Lines 119-147
The listing and parenthetical numbers become cumbersome here. Please put these numbers in a table. They will be easier to digest and compare.

Introduction

Lines 56-58
Please provide definitions of Eluc and Sland. The reader has not read the methods yet, but understanding these is central to understanding the paper. What processed are included/excluded from each?
Also, you present Sland only in the 'Overview' section, yet you call the Eluc fluxes 'net' several times in the following sections, which can be confusing. Be clear on where you discuss Sland and where you discuss only Eluc.
You should refer the ED table here.

Also, how do you segregate shifting cultivation to/from forest from deforestation/afforestation?

Overview of flux behavior

Shifted SSP scenarios

Please explain here what 'shifted' means. The reader has not yet read the methods, so they don't know what these are, and

yet they are repeatedly referred to.
Are these different than the scenarios presented in the ‘Overview’ section?

Idealized scenarios

How is wood harvest adjusted in these scenarios? It seems to be different for different scenarios, and although you state that LULCC ceases, logging continues (even though it is Land Use).

Line 154
Where is the afforestation in the NET scenarios? Is it also on pasture?

Line 175
You should explain what these legacy emissions are. What are these emissions coming from deforested land? I assume they are due continuing to soil C emissions, but then when do you transfer this process to the forest regrowth category? I mean, when does the deforested land become categorized as regrowing forest?

Lines 175-211
Please put these numbers in a table or probably two tables; one for the global values and one for the regional values. They are awkward because there are so many.

Implications

Line 225
This type of comparison is problematic because different countries/regions have different areas. To better compare between countries/regions these values should be normalized by area.

Methods

Line 271
I think Table S1 is actually extended data table 1.

Line 274
Also ED table 1?

Lines 279-280
So what is your definition of Flasc here, and how does it differ from Dorgeist?

Lines 285-292
So which of these do you use in this paper (presumably the first one?), and is Elcc the same in both cases? And how is the other definition relevant to this paper?

Line 293
Eluc/forest cannot be defined by fluxes shown in eq 3-5?

Lines 294-295
Does S* mean that you separate this forest flux for both PI and actual?

Lines 355-362
This shifting is unclear. In the previous section on historical initialization to extrapolate 2016-2020 trends to 2023. But here it sounds like you then shift 2015 ssp data to 2024, which does not align the data at all at the beginning of your simulation. This would actually revert your forcings back 8 years and create a different discontinuity. So you either exacerbate the discontinuity, or your explanation is insufficient.
It also depends on whether you are applying the annual state data, or the transition data as your lulcc forcing. There are simple methods either way to align your forcing across the boundary.

Version 1:

Reviewer comments:

Reviewer #1

(Remarks to the Author)

I appreciate the authors' efforts in resolving my issues. I recommend publication. Laibao

Reviewer #2

(Remarks to the Author)
I thank the authors for their explanations and their numerous edits to both text and figures. I think the manuscript is much clearer as a result. I have one broader question about the implications of the study and a couple small suggestions about the revised text, which are listed below.

Broad question
I understand the rationale for focusing on E_LUC for this paper, but according to Fig 1b, temperature and co2 concentrations matter quite a lot for the total land emissions. So how should I as a reader interpret the total change to emissions? One uncharitable interpretation would be that there is a negative feedback via S_land,actual (that is unaccounted for when using an offline land use model) that could offset the policy changes discussed in the rest of the paper. Another slightly more optimistic interpretation might be that we can make a dent in land use through policy, but that non-LU emissions will actually drive a lot of the LU carbon change. I think it would be really helpful to add some text - likely in the discussion but potentially elsewhere - that describes how the authors are thinking about the total impact.

Smaller comments
L384: This sentence is too vague given that not everything about the SSP is reassigned: “The first 77 years of each original SSP scenario (2015–2091) are reassigned 2024–2100.” I think it should specify what has been shifted and what has not.

L388-389 (and also L258): The parenthetical states population and non-co2 emissions, which is true. But the study is not really impacted by either of those. I think it would make more sense for it to list the relevant variables like temperature or CO2 concentrations (which are driven largely by non-land-use emissions and presumably still follow the non-shifted SSP).

Reviewer #3

(Remarks to the Author)
Review of NCOMMS-25-90983A:
National pathways of land-use CO2 emissions in the 21st century

This is a review of a revised version of this manuscript.

Overall response

The authors have done a good job addressing the reviewers’ comments. I recommend publication after addressing the minor issues below.

Specific comments/suggestions

Abstract

line 15:
be explicit here: LULCC emissions

Introduction

line 23:
land use and land-cover change

Results

lines 75-76:
Thank you for making the flux notation more consistent. I suggest in this first reporting you explicitly state the meaning of the sign: negative is land uptake and positive is emissions.

line 90:
“further contribute to variations” is vague
you can state that these values demonstrate some interaction between land cover change and climate with respect to Sland,

actual

Discussion

lines 247-253:
you should also note here the interactions between lulcc and climate that influence Sland, actual.

Methods

line 369:
i appreciate supplementary figure 2

lines 374-390:
I understand now how you are shifting the lulcc scenarios, but I am not following the reasoning when I read it. E.g., If the shift makes the lulcc inconsistent with some of the drivers of the IAMS, and also with the climate forcing, how is this preserving the “rates of change, temporal evolution, and spatial patterns of land-use change embedded in each SSP narrative under IAM-based projections”?
I think what you are trying to say is that when considering just lulcc, you want to preserve the scenario specifics as they start from a historical ending point.
The challenge is that the projections are not independent of the starting point or the RF-specific trajectory (which do tie the lulcc rates to specific years). This is just a fact of both how models work and the lulcc needs for meeting the RF targets.
Also, the shift appears to not matter much anyway, based on the figure you provided in the response.
So I think it is easier to justify aligning the lulcc rates by year, rather than shifting them, because you align your other inputs by year.
You can however, make a case for shifting, but it needs to be more clear.
Consider expanding on your justification to explain the fact that the scenarios generate projections from an historical starting point, and because you advanced your historical ending point (which you have justified) the overlapping lulcc transitions are not consistent with each other. Given the historical dependence of lulcc you want to maintain the change trajectory of the scenario, but apply it starting at your updated historical ending point.

Version 2:

Reviewer comments:

Reviewer #2

(Remarks to the Author)
The authors have addressed all my comments and I think the work is read for publication.

Reviewer #3

(Remarks to the Author)
I appreciate your clarifying revisions. Looking forward to seeing this published.

Open Access This Peer Review File is licensed under a Creative Commons Attribution 4.0 International License, which permits use, sharing, adaptation, distribution and reproduction in any medium or format, as long as you give appropriate credit to the original author(s) and the source, provide a link to the Creative Commons license, and indicate if changes were made.
In cases where reviewers are anonymous, credit should be given to 'Anonymous Referee' and the source.
The images or other third party material in this Peer Review File are included in the article's Creative Commons license, unless indicated otherwise in a credit line to the material. If material is not included in the article's Creative Commons license and your intended use is not permitted by statutory regulation or exceeds the permitted use, you will need to obtain permission directly from the copyright holder.
To view a copy of this license, visit https://creativecommons.org/licenses/by/4.0/

Reviewer #1

The authors leveraged the OSCAR model and a portfolio of LULCC and Climate scenarios to provide a set of national LULCC emission trajectories. The paper is well-written and the methodology is sound to me. I only have several questions:

Response:

Thanks for your encouraging comments. The pages that follow provide point-by-point explanations of the changes we have made in response to your suggestions.

- The author assumes the effects of land use change and climate change/variability on land carbon fluxes are independent, thereby isolating the relative contributions of land use-policies and climate forcing in driving emissions. I understand that this is a common approach in the carbon cycle research. However, as the well-know biogeophyscial effects of land use change in modulating climate (and thus carbon flux), it would be good to acknowledge this limitation and discuss this point.

Response:

We thank the reviewer for highlighting the potential role of biogeophysical land-climate feedbacks. Our simulations estimate the biogeochemical effects of land-use and land-cover change on carbon fluxes, and assume independence from the concurrent biogeophysical impacts of land-use change on climate. We acknowledge this limitation in the revised manuscript and also discuss that such a simplification of the modeling approach does not undermine this study's major conclusions.

The comparisons between low- and high-warming scenarios for a given LULCC pathway indicate that differences in atmospheric CO_2 and temperature exert a secondary influence on cumulative LULCC emissions. For example, under SSP1-2.6 and SSP3-7.0 LULCC pathways, the cumulative E_{LUC} values remain nearly identical between low- and high-warming conditions, despite large differences in climate forcing. This limited sensitivity of E_{LUC} to climate forcing is not imposed as a prior assumption, but emerges as a result of the model simulations. It reflects the fact that, within OSCAR, LULCC-driven carbon fluxes are dominated by land-use transitions themselves, particularly deforestation and regrowth, rather than by climate feedbacks.

Biogeophysical feedbacks of land-use change can locally modulate climate; however, the dominant role of deforestation dynamics, especially the sensitivity of national outcomes to policy timing and ambition, and the outsized benefits of early deforestation cessation, are robust and not affected by the simplification and neglect of biogeophysical effects in the OSCAR model simulations. We have revised the manuscript to clearly clarify this assumption, discuss the limitations, and highlight the supporting evidence from our scenario analysis based on OSCAR model simulations.

The following is the associated revision in the Methods section (see lines 370-374).

LULCC is decomposed into three components: land cover change (LCC), shifting cultivation, and wood harvest, which are illustrated in Supplementary Fig. 2. Here, the effects of LU and LCC on land carbon fluxes are simulated independently from the biogeophysical impacts of land-use change on climate, as this study focuses exclusively on the biogeochemical effects of LULCC. Climate forcing is prescribed from CO_2 and climate scenarios, allowing us to isolate the role of land-use policies and timing in shaping national LULCC emission pathways.

The following is the associated revision in the Discussion section (see lines 251-256).

Furthermore, this study assumes the impacts of LULCC on land carbon fluxes are independent of the biogeophysical effects of LULCC on climate. In reality, LULCC influences climate by modifying surface radiation, energy and water balances, and regional meteorology, thereby indirectly altering ecosystem carbon fluxes through changes in temperature, moisture availability, fire activity, and atmospheric composition (Foley et al., 2005). The comparison between low-warming and high-warming scenarios indicates that it does not affect the main conclusions of this study, as LULCC-driven carbon fluxes are dominated by LULCC rather than by climate.

- More detailed description of methods. For instance, what is S(land,PI) in Equation 2? How to derive F(LASC)?

Response:

We clarify that $S_{land, PI}$ represents the net biome productivity that would occur under preindustrial land cover, i.e., in the absence of historical land-use and land-cover change since preindustrial times. This term serves as the reference natural sink against which land-use-induced changes in sink capacity are evaluated. F_{LASC} is computed as the difference in net biome productivity between actual land cover and preindustrial land cover, weighted by the corresponding land area fractions across all land biomes. This formulation quantifies the reduction in potential carbon uptake capacity caused by land-use change since preindustrial times, following the conceptual definition of the loss of additional sink capacity (Gasser and Ciais, 2013).

The following is the associated revision in the Methods section (see lines 281-319).

The OSCAR model outputs for the terrestrial ecosystem carbon fluxes are provided annually at regional scales. A list of all variables and their subcomponents is provided in Supplementary Table 1. The three main fluxes are E_{LUC}, S_{land} and F_{LASC}, the sum of which represents the net land-to-atmosphere CO_2 flux (F_{net}):

$$F_{net} = E_{LUC} + S_{land} + F_{LASC} \tag{1}$$

Note that the definition of S_{land} in the GCB (Friedlingstein et al., 2025), that is, the natural land sink under preindustrial land cover, differs from the usual definition used with OSCAR (Gasser et al., 2020). To obtain the natural land sink under evolving land cover ($S_{land,actual}$), one must sum $S_{land,PI}$ and F_{LASC}:

$$S_{land,actual} = S_{land,PI} + F_{LASC} \tag{2}$$

where $S_{land,PI}$ represents the natural land sink under preindustrial land cover and F_{LASC} refers to the loss of additional sink capacity (LASC) due to land use changes. OSCAR characterizes F_{LASC} as the difference between the carbon uptake capacity of natural vegetation under preindustrial land cover and that under actual evolving land cover (Gasser and Ciais, 2013). This formulation of LASC corresponds to what has recently been termed Replaced Sources and Sinks (RSS) in the GCB-related paper (Friedlingstein et al., 2026). By contrast, Dorgeist et al. (2024) define LASC as a residual (i.e., synergistic) term within a factorial experimental design, which aggregates the loss of sink capacity with an interaction component that is treated separately as part of E_{LUC} in our framework.

The flux E_{LUC} is broken down into component fluxes. Two high-level components are the fluxes from LCC, E_{LCC}, and from LU, E_{LU}:

$$E_{LUC} = E_{LCC} + E_{LU} \tag{3}$$

where negative values of E_{LUC} correspond to carbon removals from the atmosphere ($E_{LUC} < 0$), whereas positive values indicate carbon emissions associated with LULCC ($E_{LUC} > 0$).

E_{LU} is further broken down into four fluxes:

$$E_{LU} = E_{harvest} + S_{harvest} + E_{shifting} + S_{shifting} \tag{4}$$

Here, $E_{harvest}$ and $S_{harvest}$ are related to wood harvest (the emission from harvested wood products (HWP), and the corresponding biospheric regrowth) and the other two to shifting cultivation (rotation cycle split between loss and gain of natural land).

We offer two possible (but incompatible) breakdowns of E_{LCC} that could be of interest to users. Both decompositions sum to the same total E_{LCC} and differ only in the attribution of subcomponents. One focused on forest, whereby we isolate the flux from deforestation ($E_{deforest}$) and the one from afforestation/reforestation ($S_{reforest}$), grouping all other LCC transitions as the remainder (F_{others}):

$$E_{LCC} = E_{deforest} + S_{reforest} + F_{others} \tag{5}$$

Another one focused on anthropogenic biomes, which is not used in the results presented here but is documented for completeness and for users interested in agricultural land-use dynamics. We isolate the fluxes induced by the establishment or abandonment of cropland or pasture, as well as by other LCC between natural land types or anthropogenic land types, and a different remainder:

$$E_{LCC} = E_{cropland} + E_{pasture} + S_{cropland} + S_{pasture} + F_{natLCC} + F_{antLCC} + F_{rest} \tag{6}$$

Finally, we also isolate two subcomponent fluxes related to the forest biome that cannot be reconstructed using other provided fluxes. The first one ($F_{forest} = E_{LUC}^{biome=forest}$) is the bookkeeping flux occurring in forest but excluding shifting cultivation; it is a part of E_{LUC} related to forests. The second one ($S_{forest} = S_{land,PI}^{biome=forest}$) is the natural land sink occurring in forest; it is a part of $S_{land,PI}$ related to forests. The sum of both gives the carbon flux over forested land, excluding shifting cultivation. Importantly, the first of these two fluxes is used to define conventional carbon dioxide removal (CDR) on forest land by Gidden et al. (2023) and in the State of CDR reports (Smith et al., 2023; Smith et al., 2024), while the second one can be used to approximate the LULCC emissions as defined by national inventories (E_{NGHGI}), following the adjustment suggested by Grassi et al. (2021) and subsequent work with OSCAR (Gidden et al., 2023):

$$E_{NGHGI} = E_{LUC} + f_{managed} \cdot S_{forest} \tag{7}$$

where $f_{managed}$ is the fraction of forested area considered managed in a given region. Note that we do not provide this final variable, as it is neither an input nor an output of the OSCAR model.

- Validation against observations in history?

Response:

Since no observations are available to directly validate our estimates of LULCC CO_2 emissions, the OSCAR model used in our study is evaluated using a GCB-based ensemble-weighting approach instead. The OSCAR Monte Carlo ensemble is constrained using the GCB2023 assessment of cumulative land–atmosphere CO_2 exchange between 1960 and 2022. Ensemble members are weighted according to their agreement with the assessed global land-use carbon flux (i.e., 150 ± 35 Pg C over 1960–2022), excluding the flux combinations inconsistent with such observations.

The following is the associated revision in the Methods section (see lines 340-348).

The constraint is the estimate of $S_{land,actual}$ over the instrumental observation period (from 1960 onwards), provided by the GCB2023, which is currently 150±35 Pg C over 1960-2022 (Friedlingstein et al., 2023). The ensemble is evaluated over the same period to ensure consistency between the simulated and observationally constrained quantities. Here, we note that while we have demonstrated the strength of using regional constraints to better match national inventories (He et al., 2024), this is not yet implemented in this standard GCB setup.

The weights are then used to calculate the weighted average and weighted standard deviation of the simulation results. Note that the values for OSCAR reported in the GCB are the weighted average only. However, since our goal is to extend these historical simulations to future periods, we run the same 4000 ensemble members for every single scenario (a total of 150) in this work and provide weighted outputs, following the standard OSCAR configuration used in the GCB.

Minor comments:

- The model and data are publicly available, so do your Codes.

Response:

We have made the scripts that perform the analysis and produce the figures in this study available at https://doi.org/10.5281/zenodo.16729724 (Zhang et al., 2026).

- Some references missing, such as Line 271, 275, etc.

Response:

All missing references have now been added, and the manuscript has been thoroughly checked to ensure that all citations are complete and correctly linked.

- Add some tables for national-level statistics

Response:

We have added national- and regional-level summary tables to the manuscript, including Supplementary Tables 2–4, which report cumulative E_{LUC} components, regional contributions, and forest area under different scenarios.

The following is the associated revision in the Supplementary Information.

Supplementary Table 2. Cumulative contributions of E_{LUC} components across LULCC scenarios under low warming in 2024-2100 (Unit: Pg C).

Scenario	$E_{deforest}$	$S_{reforest}$	$E_{harvest} + S_{harvest}$	$E_{shifting} + S_{shifting}$	F_{others}	E_{LUC}
SSP1-1.9	36.7	-44.1	-3.4	-4.4	3.4	-12.0
SSP1-2.6	36.9	-44.2	-3.5	-4.6	1.7	-13.6
SSP2-4.5	40.9	-40.8	2.8	-3.9	10.0	9.0
SSP3-7.0	77.0	-32.1	1.9	-5.0	15.0	56.7
SSP4-3.4	121.1	-50.4	6.6	-7.2	19.4	89.4
SSP4-6.0	64.3	-36.6	7.4	-6.4	13.0	41.7
SSP5-3.4-OS	61.5	-38.6	6.1	-4.0	18.5	43.4
SSP5-8.5	45.7	-26.9	4.8	-3.7	11.0	30.8
Commit	12.7	-18.2	-22.0	-23.6	-1.2	-52.3
BAU2030	25.5	-24.1	1.3	11.4	-0.8	13.2
Forest2100	25.5	-109.6	1.3	11.4	-0.7	-72.2
Gross2030	14.9	-24.1	1.2	-20.9	-0.8	-29.7
Gross2050	20.8	-24.1	1.2	-20.4	-0.8	-23.2
Net2030	15.9	-24.1	1.3	11.4	-0.8	3.7
Net2050	21.2	-24.1	1.3	11.4	-0.8	9.0

Supplementary Table 3. Cumulative regional contributions of E_{LUC} across LULCC scenarios under low warming in 2024-2100 (Unit: Pg C). Regions include China (CHN), Indonesia (IDN), Brazil (BRA), Democratic Republic of the Congo (COD), United States (USA), Russia (RUS), India (IND), Angola (AGO), Peru (PER), Tanzania (TZA), and Colombia (COL).

Scenario	CHN	IDN	BRA	COD	USA	RUS	IND	AGO	PER	TZA	COL
SSP1-1.9	-8.9	-3.8	3.8	1.4	-1.7	-1.5	-0.8	1.4	0.2	0.6	-0.5
SSP1-2.6	-8.6	-4.0	3.4	1.5	-1.6	-1.7	-0.8	0.5	0.2	0.7	-0.4
SSP2-4.5	-9.0	0.5	6.0	5.2	-1.5	-1.5	-0.2	0.9	0.4	0.8	-0.6
SSP3-7.0	-12.7	-1.5	6.8	19.6	4.3	-1.9	1.3	4.2	5.5	1.3	0.0
SSP4-3.4	0.1	12.7	-18.4	22.8	5.4	-3.3	2.1	6.5	0.4	2.1	-1.1
SSP4-6.0	-4.3	-3.1	-8.2	19.5	2.8	-1.3	1.3	4.9	0.3	1.9	-0.5
SSP5-3.4-OS	-4.7	-1.6	11.6	9.7	6.1	-1.5	0.8	1.2	0.3	3.1	0.0
SSP5-8.5	-5.3	-3.0	10.8	8.6	3.3	-0.7	0.8	0.7	0.3	3.6	-0.6
Commit	-7.2	-6.1	-4.8	-2.1	-2.7	-2.6	-3.2	0.0	0.1	0.0	-1.2
BAU2030	-6.3	2.6	8.1	3.1	0.5	-0.7	-0.4	0.9	0.8	0.6	-0.4
Forest2100	-11.1	1.8	-5.9	0.3	-7.2	-3.6	-1.1	-2.1	0.8	0.4	-1.4
Gross2030	-7.1	-4.4	-2.0	-1.3	-0.1	-0.7	-1.0	0.3	0.2	0.3	-1.6
Gross2050	-6.7	-4.2	-0.2	-0.7	0.3	-0.7	-0.9	0.5	0.4	0.4	-1.4
Net2030	-6.8	2.5	4.6	2.5	-0.1	-0.7	-0.4	0.5	0.5	0.5	-0.6
Net2050	-6.5	2.6	6.6	2.9	0.2	-0.7	-0.4	0.7	0.7	0.6	-0.5

Supplementary Table 4. Forest area (Mha) in 2023 and 2100 under different scenarios. Regions include

global, Brazil (BRA), Canada (CAN), China (CHN), Democratic Republic of the Congo (COD), European Union (EU), Indonesia (IDN), Russia (RUA), and the United States of America (USA).

Scenario (year)	Global	BRA	CAN	CHN	COD	EU	IDN	RUS	USA
Historical (2023)	3914.2	471.0	445.6	227.6	135.1	149.3	102.1	833.2	303.9
SSP1-1.9 (2100)	3990.6	473.2	448.3	266.9	131.4	153.7	107.5	834.1	318.4
SSP1-2.6 (2100)	3967.1	468.9	447.8	261.2	131.4	151.5	107.2	834.5	311.0
SSP2-4.5 (2100)	3968.8	488.7	439.9	262.9	127.6	152.0	90.7	840.9	331.9
SSP3-7.0 (2100)	3593.1	445.0	430.9	287.2	13.9	145.4	96.4	840.3	266.0
SSP4-3.4 (2100)	3449.8	564.0	450.1	214.8	16.6	153.9	25.7	846.3	261.5
SSP4-6.0 (2100)	3765.4	534.1	451.4	248.6	32.6	153.0	106.9	852.1	295.1
SSP5-3.4-OS (2100)	3779.0	453.2	443.6	255.1	95.6	147.2	99.9	829.5	274.0
SSP5-8.5 (2100)	3789.6	451.3	444.5	241.9	84.0	146.4	114.3	834.5	282.7
BAU2030 (2100)	3842.7	435.6	446.5	242.8	125.6	152.9	100.2	833.6	298.0
Commit (2100)	3910.5	469.4	445.6	228.9	134.5	149.4	101.8	833.1	303.5
Forest2100 (2100)	4849.1	585.2	450.2	287.0	154.8	173.1	106.6	878.1	390.8
Gross2030 (2100)	3941.9	465.3	446.8	247.1	132.8	153.8	101.5	833.9	302.8
Gross2050 (2100)	3885.7	451.0	446.6	244.4	126.6	153.5	101.0	833.7	299.3
Net2030 (2100)	3933.8	464.0	446.5	245.6	132.8	153.2	101.6	833.8	302.6
Net2050 (2100)	3883.2	448.3	446.5	244.2	128.7	153.0	100.7	833.7	300.1

Reference

Dorgeist, L., Schwingshackl, C., Bultan, S., and Pongratz, J.: A consistent budgeting of terrestrial carbon fluxes, Nature Communications, 15, 7426, http://doi.org/10.1038/s41467-024-51126-x, 2024.

Foley, J. A., DeFries, R., Asner, G. P., Barford, C., Bonan, G., Carpenter, S. R., Chapin, F. S., Coe, M. T., Daily, G. C., Gibbs, H. K., Helkowski, J. H., Holloway, T., Howard, E. A., Kucharik, C. J., Monfreda, C., Patz, J. A., Prentice, I. C., Ramankutty, N., and Snyder, P. K.: Global Consequences of Land Use, Science, 309, 570-574, http://doi.org/10.1126/science.1111772, 2005.

Friedlingstein, P., Le Quéré, C., O'Sullivan, M., Hauck, J., Landschützer, P., Luijkx, I. T., Li, H., van der Woude, A., Schwingshackl, C., Pongratz, J., Regnier, P., Andrew, R. M., Bakker, D. C. E., Canadell, J. G., Ciais, P., Gasser, T., Jones, M. W., Lan, X., Morgan, E., Olsen, A., Peters, G. P., Peters, W., Sitch, S., and Tian, H.: Emerging climate impact on carbon sinks in a consolidated carbon budget, Nature, 649, 98-103, 10.1038/s41586-025-09802-5, 2026.

Friedlingstein, P., O'Sullivan, M., Jones, M. W., Andrew, R. M., Hauck, J., Landschützer, P., Le Quéré, C., Li, H., Luijkx, I. T., Olsen, A., Peters, G. P., Peters, W., Pongratz, J., Schwingshackl, C., Sitch, S., Canadell, J. G., Ciais, P., Jackson, R. B., Alin, S. R., Arneth, A., Arora, V., Bates, N. R., Becker, M., Bellouin, N., Berghoff, C. F., Bittig, H. C., Bopp, L., Cadule, P., Campbell, K., Chamberlain, M. A., Chandra, N., Chevallier, F., Chini, L. P., Colligan, T., Decayeux, J., Djeutchouang, L. M., Dou, X., Duran Rojas, C., Enyo, K., Evans, W., Fay, A. R., Feely, R. A., Ford, D. J., Foster, A., Gasser, T., Gehlen, M., Gkritzalis, T., Grassi, G., Gregor, L., Gruber, N., Gürses, Ö., Harris, I., Hefner, M., Heinke, J., Hurtt, G. C., Iida, Y., Ilyina, T., Jacobson, A. R., Jain, A. K., Jarníková, T., Jersild, A., Jiang, F., Jin, Z., Kato, E., Keeling, R. F., Klein Goldewijk, K., Knauer, J., Korsbakken, J. I., Lan, X., Lauvset, S. K., Lefèvre, N., Liu, Z., Liu, J., Ma, L., Maksyutov, S., Marland, G., Mayot, N., McGuire, P. C., Metzl, N.,

Monacci, N. M., Morgan, E. J., Nakaoka, S. I., Neill, C., Niwa, Y., Nützel, T., Olivier, L., Ono, T., Palmer, P. I., Pierrot, D., Qin, Z., Resplandy, L., Roobaert, A., Rosan, T. M., Rödenbeck, C., Schwinger, J., Smallman, T. L., Smith, S. M., Sospedra-Alfonso, R., Steinhoff, T., Sun, Q., Sutton, A. J., Séférian, R., Takao, S., Tatebe, H., Tian, H., Tilbrook, B., Torres, O., Tourigny, E., Tsujino, H., Tubiello, F., van der Werf, G., Wanninkhof, R., Wang, X., Yang, D., Yang, X., Yu, Z., Yuan, W., Yue, X., Zaehle, S., Zeng, N., and Zeng, J.: Global Carbon Budget 2024, Earth Syst. Sci. Data, 17, 965-1039, http://doi.org/10.5194/essd-17-965-2025, 2025.

Friedlingstein, P., O'Sullivan, M., Jones, M. W., Andrew, R. M., Bakker, D. C. E., Hauck, J., Landschützer, P., Le Quéré, C., Luijkx, I. T., Peters, G. P., Peters, W., Pongratz, J., Schwingshackl, C., Sitch, S., Canadell, J. G., Ciais, P., Jackson, R. B., Alin, S. R., Anthoni, P., Barbero, L., Bates, N. R., Becker, M., Bellouin, N., Decharme, B., Bopp, L., Brasika, I. B. M., Cadule, P., Chamberlain, M. A., Chandra, N., Chau, T. T. T., Chevallier, F., Chini, L. P., Cronin, M., Dou, X., Enyo, K., Evans, W., Falk, S., Feely, R. A., Feng, L., Ford, D. J., Gasser, T., Ghattas, J., Gkritzalis, T., Grassi, G., Gregor, L., Gruber, N., Gürses, Ö., Harris, I., Hefner, M., Heinke, J., Houghton, R. A., Hurtt, G. C., Iida, Y., Ilyina, T., Jacobson, A. R., Jain, A., Jarníková, T., Jersild, A., Jiang, F., Jin, Z., Joos, F., Kato, E., Keeling, R. F., Kennedy, D., Klein Goldewijk, K., Knauer, J., Korsbakken, J. I., Körtzinger, A., Lan, X., Lefèvre, N., Li, H., Liu, J., Liu, Z., Ma, L., Marland, G., Mayot, N., McGuire, P. C., McKinley, G. A., Meyer, G., Morgan, E. J., Munro, D. R., Nakaoka, S. I., Niwa, Y., O'Brien, K. M., Olsen, A., Omar, A. M., Ono, T., Paulsen, M., Pierrot, D., Pocock, K., Poulter, B., Powis, C. M., Rehder, G., Resplandy, L., Robertson, E., Rödenbeck, C., Rosan, T. M., Schwinger, J., Séférian, R., Smallman, T. L., Smith, S. M., Sospedra-Alfonso, R., Sun, Q., Sutton, A. J., Sweeney, C., Takao, S., Tans, P. P., Tian, H., Tilbrook, B., Tsujino, H., Tubiello, F., van der Werf, G. R., van Ooijen, E., Wanninkhof, R., Watanabe, M., Wimart-Rousseau, C., Yang, D., Yang, X., Yuan, W., Yue, X., Zaehle, S., Zeng, J., and Zheng, B.: Global Carbon Budget 2023, Earth Syst. Sci. Data, 15, 5301-5369, http://doi.org/10.5194/essd-15-5301-2023, 2023.

Gasser, T. and Ciais, P.: A theoretical framework for the net land-to-atmosphere CO₂ flux and its implications in the definition of "emissions from land-use change", Earth Syst. Dynam., 4, 171-186, http://doi.org/10.5194/esd-4-171-2013, 2013.

Gasser, T., Crepin, L., Quilcaille, Y., Houghton, R. A., Ciais, P., and Obersteiner, M.: Historical CO2 emissions from land use and land cover change and their uncertainty, Biogeosciences, 17, 4075-4101, https://doi.org/10.5194/bg-17-4075-2020, 2020.

Gidden, M. J., Gasser, T., Grassi, G., Forsell, N., Janssens, I., Lamb, W. F., Minx, J., Nicholls, Z., Steinhauser, J., and Riahi, K.: Aligning climate scenarios to emissions inventories shifts global benchmarks, Nature, 624, 102-108, http://doi.org/10.1038/s41586-023-06724-y, 2023.

Grassi, G., Stehfest, E., Rogelj, J., van Vuuren, D., Cescatti, A., House, J., Nabuurs, G.-J., Rossi, S., Alkama, R., Viñas, R. A., Calvin, K., Ceccherini, G., Federici, S., Fujimori, S., Gusti, M., Hasegawa, T., Havlik, P., Humpenöder, F., Korosuo, A., Perugini, L., Tubiello, F. N., and Popp, A.: Critical adjustment of land mitigation pathways for assessing countries' climate progress, Nature Climate Change, 11, 425-434, http://doi.org/10.1038/s41558-021-01033-6, 2021.

He, Y., Piao, S., Ciais, P., Xu, H., and Gasser, T.: Future land carbon removals in China consistent with national inventory, Nature Communications, 15, 10426, http://doi.org/10.1038/s41467-024-54846-2, 2024.

Smith, S. M., Geden, O., Gidden, M. J., Lamb, W. F., Nemet, G. F., Minx, J. C., Buck, H., Burke, J., Cox, E., Edwards, M. R., Fuss, S., Johnstone, I., Müller-Hansen, F., Pongratz, J., Probst, B. S., Roe, S.,

Schenuit, F., Schulte, I., and Vaughan, N. E.: The State of Carbon Dioxide Removal - 2nd Edition, The State of Carbon Dioxide Removal, https://doi.org/10.17605/OSF.IO/F85QJ, 2024.

Smith, S. M., Geden, O., Nemet, G. F., Gidden, M. J., Lamb, W. F., Powis, C., Bellamy, R., Callaghan, M. W., Cowie, A., Cox, E., Fuss, S., Gasser, T., Grassi, G., Greene, J., Lück, S., Mohan, A., Müller-Hansen, F., Peters, G. P., Pratama, Y., Repke, T., Riahi, K., Schenuit, F., Steinhauser, J., Strefler, J., Valenzuela, J. M., and Minx, J. C.: The State of Carbon Dioxide Removal - 1st Edition, The State of Carbon Dioxide Removal, https://doi.org/10.17605/OSF.IO/W3B4Z, 2023.

Zhang, D., Zheng, B., and Gasser, T.: Land Carbon Fluxes Projected by OSCAR [dataset], https://doi.org/10.5281/zenodo.16729724, 2026.

Reviewer #2 (Remarks to the Author):

In the manuscript National pathways of land-use CO_2 emissions in the 21st century, the authors use a set of SSP and idealized land use scenarios to quantify land use emissions and better understand their drivers. They find that there is a lot of variation in net carbon emissions, driven largely by the fate of forests. They also demonstrate that temperature change is a very small portion of the total variability, and land use decisions drive most variation. The authors also share the datasets they produce, which could be a helpful resource for future studies.

Overall I found the paper compelling but I have a number of comments about methodological clarity and methodological decisions that I think should be addressed before recommending the paper for publication.

Response:

We thank the reviewer for the positive and constructive assessment of our manuscript. We have carefully addressed all comments regarding methodological clarity and methodological choices, and have revised the manuscript accordingly.

Major comments

There were multiple elements of the methods that I found quite confusing, which need significant clarification.

1) The section about the shifted SSPs was not clear. L358 says something about "preserving land-use policy information that is typically more intense at the beginning of the scenarios" which was not well described. If the 2015-2024 period had a lot of the LU policy, wouldn't we already be on one of the trajectories? Why would we assume that 2015 policies apply in 2025? I appreciate that the authors are clear that they are using shifted SSPs, but it's not clear to me from the text that this decision is preferable to a different harmonization.

Response:

SSP land-use scenarios are generated by IAMs as internally consistent socio-economic pathways, in which land-use change emerges from the coupled evolution of population, economic development, energy systems, agricultural demand, and land-use policies. As such, SSP trajectories should not be interpreted as literal representations of policies enacted since specific calendar years, but rather as stylized narratives that are characterized by distinct rates of change, transition timing, and long-term land-use dynamics. There is therefore no reason that scenarios created before 2015 would represent the reality of what happened since then.

In IAM-based SSP projections, the early phase of each scenario typically contains the

strongest adjustments in land-use trajectories, reflecting the initial divergence of socio-economic pathways rather than the implementation of real-world policies tied to the year 2015. Under each SSP scenario, the total area of land use change between 2015 and 2023 is larger than that between 2091 and 2100 (Table R1), indicating the 2015-2023 data contains more narrative information.

Table R1. The total area of land use change under each SSP scenario from 2015 to 2023 and from 2091 to 2100 (Unit: Mha).

	SSP119	SSP126	SSP245	SSP370	SSP434	SSP460	SSP5-3.4-OS	SSP585
2015-2023 sum	177	176	239	338	268	216	146	191
2091-2100 sum	111	155	168	148	230	175	16	66

Because our simulations must start after the end of the observation-constrained historical period used in the Global Carbon Budget (currently 2023), we apply a time shift to the SSP land-use change data to preserve these internally consistent dynamics and rates of change while ensuring continuity from historical land-use states. This approach assumes that the structural land-use dynamics described by each SSP narrative unfold from the end of the historical period onward.

Alternative harmonization strategies, such as truncating the initial SSP years or interpolating land-use variables to match the historical endpoint, would disrupt the dynamic consistency of the underlying IAM solutions and could introduce non-physical discontinuities in land-use change rates. In this specific case, because a significant fraction of the LU policy happens before 2023, it would significantly flatten the ensemble of scenarios, so that there would be much less difference between them. We therefore adopt the shifted SSP approach as a pragmatic and reproducible harmonization strategy, consistent with the philosophy of LUH2 (Hurtt et al., 2020), in the absence of officially updated SSP land-use datasets extending beyond 2015.

<u>The following is the associated revision in the Methods section (see lines 376-392).</u>

The LULCC data under eight SSP scenarios are taken from the LUH2 dataset for CMIP6 (Hurtt et al., 2020), representing internally consistent socio-economic pathways generated by IAMs rather than predictions tied to specific calendar years.

The LUH2 transition data under SSP scenarios are provided from 2015 onwards, whereas our simulations must start in 2024, immediately after the end of the observation-constrained historical period used in the GCB2024. To ensure continuity at the transition from historical land-use conditions, while preserving the internally consistent dynamics produced by IAMs, we apply a time shift to the SSP LULCC transition data. These methods leverage the SSP-derived annual LULCC rates starting from the 2023 observational baseline.

The first 77 years of each original SSP scenario (2015–2091) are reassigned 2024–2100. Historical LCC is simulated up to 2023, and the shifted SSP LCC data are then appended from 2024 onward. For LU components (wood harvest and shifting cultivation), an offset is applied using the mean of the last 11 years (2013–2023) as a reference, consistent with the treatment of CO_2 and climate forcings.

We recognize that this means the new LULCC data is inconsistent with other aspects of these scenarios (e.g., non-CO_2 emissions, population). Still, it is difficult to maintain consistency across all aspects while changing the starting year of a scenario. This approach preserves the rates of change, temporal

evolution, and spatial patterns of land-use change embedded in each SSP narrative under IAM-based projections. Cumulative LULCC emissions from IAMs are taken over their native scenario period (2015–2091) and used as a benchmark to contextualize OSCAR-based estimates under the corresponding shifted SSP land-use pathways, without serving as model inputs or constraints.

2) I think the idealized scenarios need more justification. For example, although the BAU scenario is described in the methods, no description is provided for why these choices were made. A more obvious choice to me would have been to extrapolate out current trends indefinitely or keep existing levels constant. I don't understand why current trends continuing until 2030 and then changing is "business as usual". My comment on L254 is similar but for the Forest scenario.

Response:

The BAU2030 scenario is explicitly designed as the baseline against which all other idealized scenarios are defined and compared. It represents a constrained continuation of recent land-use dynamics, in which regional LULCC trends over 2013–2023 are linearly extrapolated to 2030 to capture near-term momentum, followed by a gradual phase-out of land-cover change and stabilization of land-use areas thereafter. This structure ensures internal consistency across all counterfactual experiments.

The choice of 2030 as the transition year is policy-motivated. At COP26 in Glasgow, 141 countries committed to halting and reversing forest loss by 2030. The Gross and Net deforestation scenarios are constructed based on this milestone. To ensure compatibility across scenarios, the BAU2030 scenario adopts the same reference year.

In our previous peer-reviewed work (Gasser et al., 2022) that focused on projections to 2050, the BAU scenario extrapolated recent land-use trends to 2030 and held them constant thereafter. Extending this formulation to 2100 may lead to non-physical outcomes in all regions (e.g., negative land areas or unrealistically dominant land-use types). For a long time horizon considered here, it is thus necessary to progressively decrease land-cover change rates after 2030 to respect land availability constraints.

<u>The following is the associated revision in the Methods section (see lines 404-410).</u>

Business As Usual (BAU2030). The Business As Usual (BAU2030) scenario represents a constrained continuation of current land-use dynamics in the absence of additional policy intervention, serving as the baseline for all idealized scenarios. All LULCC in each region from 2023 to 2030 are linearly extrapolated from the trends of the past 11 years (currently, 2013-2023). After that, LCC is assumed to linearly reach zero by 2050 and LU is held constant at 2030 levels. This transition is introduced to prevent non-physical outcomes over long time horizons, such as land-use changes exceeding available land area or unrealistic dominance of a single land-use type by 2100. The choice of 2030 as the transition year reflects its central role in the COP26 commitment to halt and reverse forest loss by 2030.

The Forest2100 scenario is not intended as an alternative "business-as-usual" trajectory, nor as an extrapolation of recent afforestation trends. It is designed as an

idealized, policy-relevant counterfactual scenario, constructed as a perturbation on top of the BAU2030 baseline. This design allows the carbon-cycle impacts of intensified afforestation to be isolated while keeping all other land-use drivers consistent.

The choice of 2030 as the transition year mirrors the BAU2030 baseline and reflects the same policy-relevant time horizon, i.e., the COP26 commitment to halt and reverse forest loss by 2030. Afforestation rates are increased progressively between 2024 and 2030, after which a constant rate is applied to reach a regionally constrained maximum forest area by 2100. This formulation avoids non-physical outcomes over long time horizons and ensures consistency with land availability constraints.

The following is the associated revision in the Methods section (see lines 411-419).

Intense Afforestation (Forest2100). The Forest2100 scenario assesses the potential carbon sinks that could result from ambitious afforestation policies. In this scenario, intensified afforestation on pasture land aims to reach a regionally constrained global maximum forest area (935Mha) in 2100, following Moustakis et al. (2024). All other LULCC activities follow the BAU2030 scenario. In each region, we calculated the forest area change that occurred between 2015 and 2023, as well as the projected forest area change associated with other land-use change activities under the BAU2030 scenario over the period 2024–2100. These quantities were subtracted from the regional potential forest area; the remaining area represents the forest area increase required in each region over 2023–2100. This additional forest area is then allocated over time by assuming a linear increase in annual afforestation rates from 2024 to 2030, followed by a constant annual afforestation rate thereafter.

3) The use of N and N-1 (and even N-2100) is pervasive throughout the methods and I found it very difficult to follow. Unless there is an explicit reason not to, please just state the years instead.

Response:

The notation using N was introduced to denote the publication year of the Global Carbon Budget, as the dataset is designed for regularly annual updates. For this manuscript, N corresponds to GCB 2024, with historical simulations up to 2023 and projections starting in 2024. To improve readability, we have replaced N, N−1, and related notation with explicit years in the Methods section of the revised manuscript.

Line comments

57: Unclear what “second” is referring to

Response:

The second term referrs to the land carbon sink under actual land use. In the revised manuscript, we explicitly name these terms rather than referring to them ordinally.

The following is the associated revision in the Introduction section (see lines 60-61).

We nevertheless also report estimates of the land sink under pre-industrial land cover ($S_{land,PI}$) and the land sink under actual land cover ($S_{land,actual}$) to facilitate comparison with national inventories.

65: I found this description confusing. Why compare low warming? I thought the two scenarios are compared with high and low warming.

Response:

Our analysis is based on a controlled-factor experimental design in which land-use and climate influences are isolated sequentially. To quantify the effect of land-use trajectories, we compare SSP1-2.6 and SSP3-7.0 LULCC scenarios under a fixed low-warming climate and CO_2 pathway. This choice minimizes climate-driven influences and allows differences in carbon fluxes to be only attributed to land-use decisions. We then quantify the effect of climate change by holding the LULCC trajectory fixed (SSP3-7.0) and comparing low- and high-warming condition.

We have revised the text to explicitly describe this two-step factorial approach.

The following is the associated revision in the Results section (see lines 67-73).

The low-warming scenario is based on the climate and atmospheric CO_2 conditions of SSP1-2.6, whereas the high-warming scenario follows SSP3-7.0. Each warming scenario is combined with alternative LULCC pathways to disentangle the relative contributions of land-use decisions and warming forcing to terrestrial carbon fluxes. Comparison between SSP1-2.6 and SSP3-7.0 LULCC under the fixed low-warming isolates the influence of land-use trajectories. This comparison minimizes warming-driven feedbacks and allows differences in carbon fluxes to be attributed primarily to contrasting land-use strategies. We then quantify the effect of climate change by holding the LULCC trajectory constant (SSP3-7.0) and comparing results under low- and high-warming conditions.

Fig 1b: The blue lines are too hard to see - especially the solid one. Also are both shadings shown or just the blue line? Can't tell as-is

Response:

For $S_{land,\,actual}$, the SSP1-2.6 and SSP3-7.0 LULCC scenarios produce nearly identical trajectories under same warming. This overlap reflects a physical result rather than a plotting artifact: $S_{land,\,actual}$ is primarily controlled by atmospheric CO_2 concentrations and climate forcing, with a weak sensitivity to land-use trajectories.

To improve readability and explicitly convey this result, we have revised Fig. 1b in two ways. First, we reduced line widths to limit visual masking where trajectories overlap. Second, we added a distinct hatching pattern to the uncertainty shading of the SSP1-2.6 LULCC scenario, allowing the uncertainty envelopes of the two LULCC scenarios to be clearly distinguished even where their central estimates coincide.

The following is the associated revision in the Results section (see page 3).

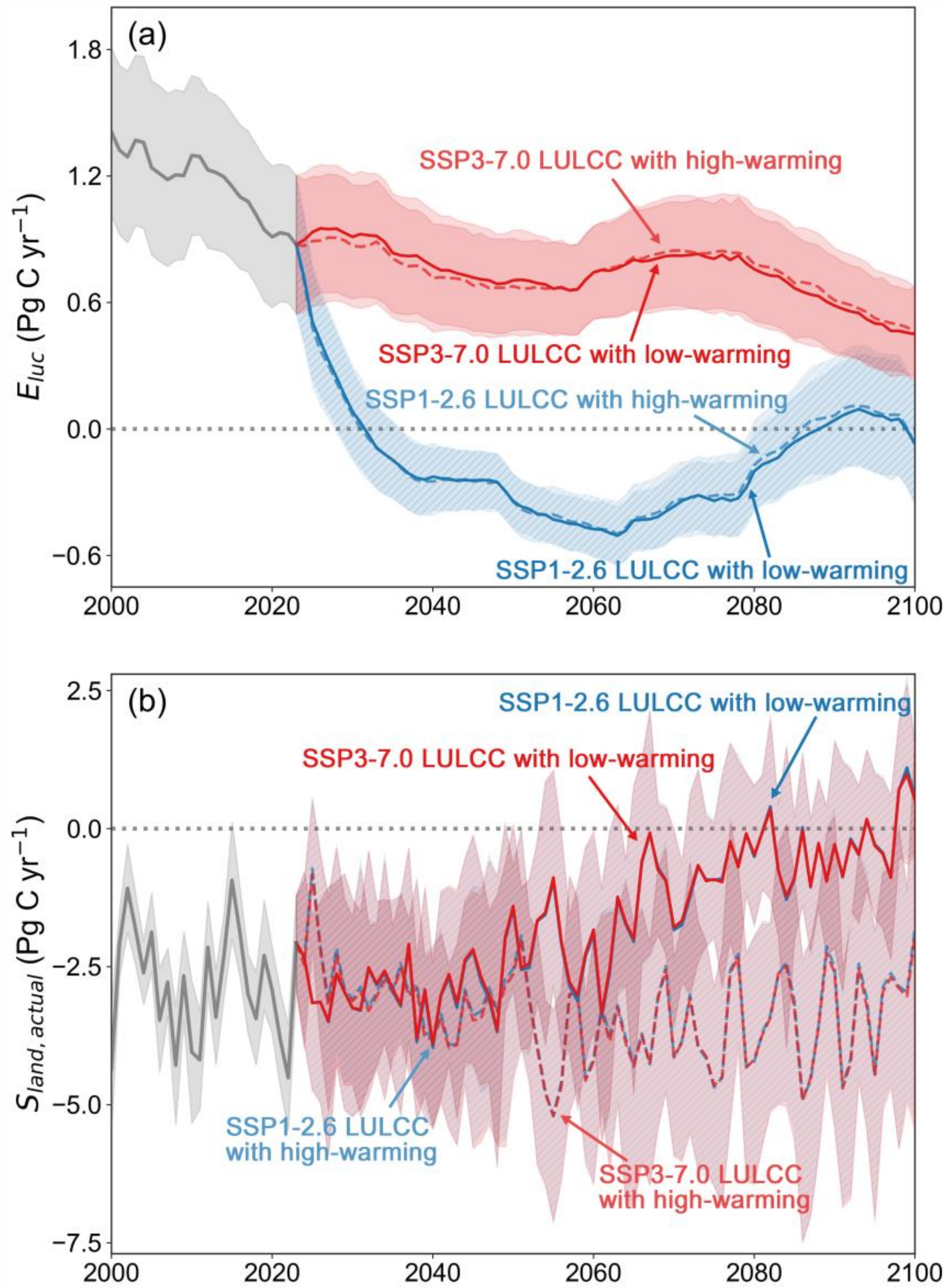

Fig. 1. Main carbon fluxes under SSP1-2.6 and SSP3-7.0 LULCC scenarios. (a) E_{LUC} changes from 2000 to 2100 and (b) $S_{land,actual}$ changes from 2000 to 2100. Gray lines represent historical values from 2000 to 2023. Blue lines indicate projected values under the SSP1-2.6 LULCC scenario, and red lines indicate projected values under the SSP3-7.0 LULCC scenario. Solid lines correspond to the low-warming pathway and dashed lines to the high-warming pathway. Shaded areas denote uncertainty ranges, shown as hatched shading for the SSP1-2.6 LULCC scenario and solid shading for the SSP3-7.0 LULCC scenario.

98: After Fig 1 it's unclear what temperature is being used to drive results. I realize it doesn't make a big difference given the Fig 1 results, but it should be specified. As far as I can tell the Figure 5 caption is the only indication.

Response:

We have now clarified this point that all results presented in Figs. 2–5 are driven by the low-warming climate and CO_2 forcing corresponding to SSP1-2.6.

The following is the associated revision in the Results section (see lines 99-102).

Given its direct link to land-use policy and its limited sensitivity to climate feedbacks, we focus in the following sections on E_{LUC}. All subsequent analyses of shifted SSP and idealized LULCC scenarios are conducted under the low-warming climate and atmospheric CO_2 pathway. Results for all other combinations of climate and LULCC scenarios are provided in the accompanying dataset

(https://doi.org/10.5281/zenodo.16729724).

107: “Intermediate and high-emission scenarios…” is written as though it’s a result, but I think it’s an input

Response:

We have revised the text to explicitly distinguish between (i) the prescribed land-use and socio-economic characteristics of the SSP scenarios, which constitute model inputs, and (ii) the simulated E_{LUC} responses based on the OSCAR model.

The following is the associated revision in the Results section (see lines 117-118).

Intermediate- and high-emission SSPs prescribe weaker land-use mitigation and exhibit more intensive land-use emissions.

Fig 2: The colors look nice, but they are so similar that it’s very tempting to read them all as SSPs. Especially for panel (b) the it’s not obvious which legend the colors correspond to.

Response:

To avoid confusion between SSP scenarios and LULCC emission flux components, we revised the visualization of the emission components. Specifically, we adopted a desaturated color scheme for the flux components and changed their line style to dashed lines. These adjustments distinguish the component-specific fluxes from the SSP scenarios while maintaining consistency with the official SSP color convention.

The following is the associated revision in the Results section (see page 5).

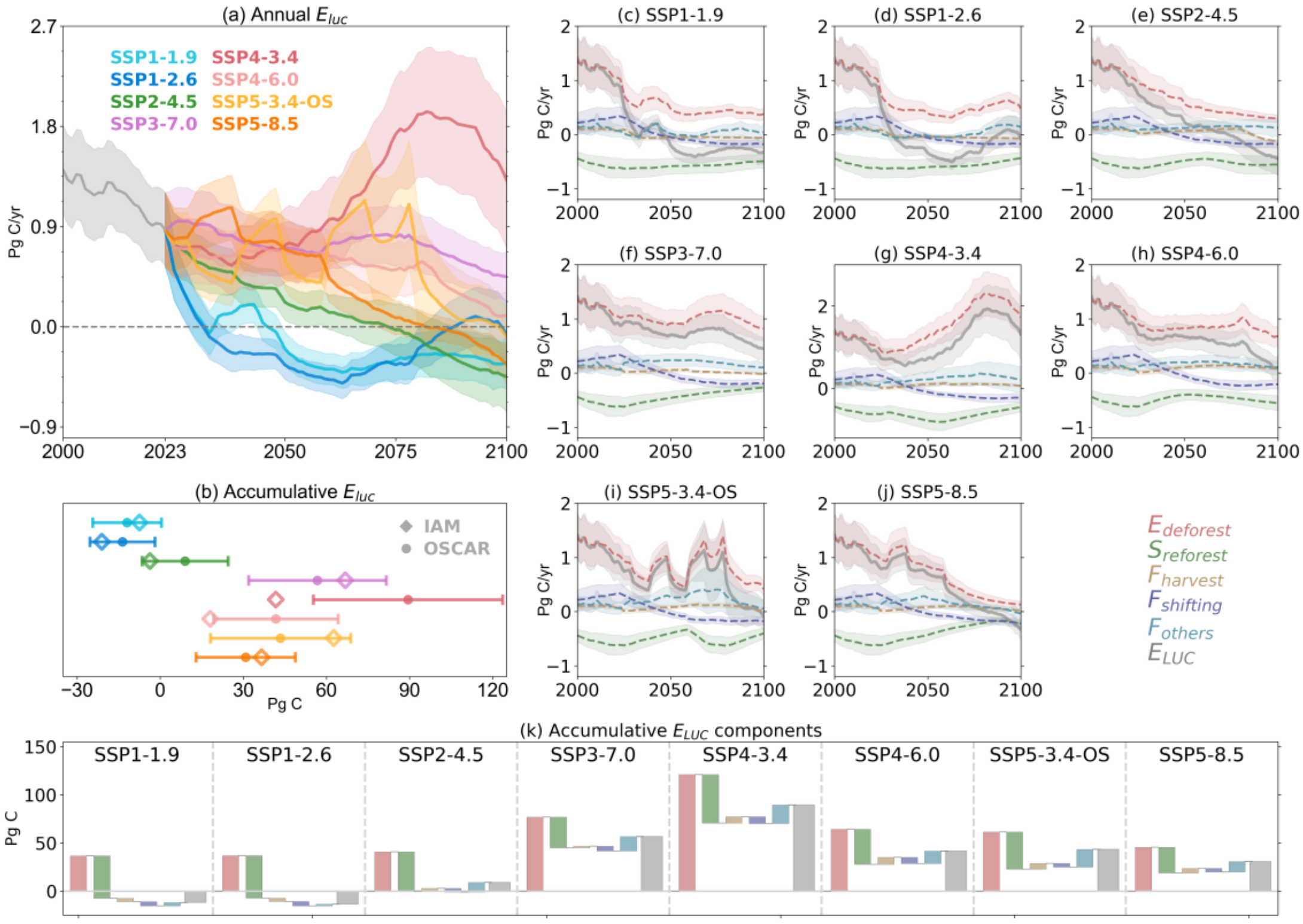

Fig. 2. Global E_{LUC} under shifted SSP scenarios driven by the low-warming climate and atmospheric CO_2 forcing. (a) Annually global E_{LUC} under shifted SSP scenarios in 2000-2100. (b) Accumulative global E_{LUC} under shifted SSP scenarios in 2024-2100. The error bars represent the accumulative global E_{LUC} under shifted SSP scenarios during the period 2024-2100 in this study. The diamond markers indicate the accumulative global E_{LUC} simulated by the IAMs over the native scenario period (2015–2091), used here for reference. (c-j) Global E_{LUC} components from 2024 to 2100 under different SSP scenarios. (k) Globally accumulative E_{LUC} components in 2024-2100 under each SSP scenario. Exact cumulative values for each ELUC component and scenario are reported in Supplementary Table 2.

Fig 2 description: "To ensure temporal consistency, we adopted the IAMs-derived accumulative values for the period 2015-2092" This is a very confusing sentence. I think I might understand after a close read of the methods, but it makes little sense here.

Response:

We have clarified in the Methods that the IAM-derived cumulative E_{LUC} values are used as a reference to contextualize OSCAR estimates under the shifted SSP scenarios. The Fig. 2 caption has been revised to retain this information in a concise form.

The following is the associated revision in the Results section (see lines 125-127).

The error bars represent the accumulative global E_{LUC} under shifted SSP scenarios during the period 2024-2100 in this study. The diamond markers indicate the accumulative global E_{LUC} simulated by the IAMs over the native scenario period (2015–2091), used here for reference.

The following is the associated revision in the Methods section (see lines 389-392).

This approach preserves the rates of change, temporal evolution, and spatial patterns of land-use change embedded in each SSP narrative under IAM-based projections. Cumulative LULCC emissions

from IAMs are taken over their native scenario period (2015–2091) and used as a benchmark to contextualize OSCAR-based estimates under the corresponding shifted SSP land-use pathways, without serving as model inputs or constraints.

Fig 3: Labels start at (c) and corresponding text references are off

Response:

We have corrected Fig. 3 so that the panel labels now start at (a) and all corresponding text references have been updated accordingly.

The following is the associated revision in the Results section (see page 6).

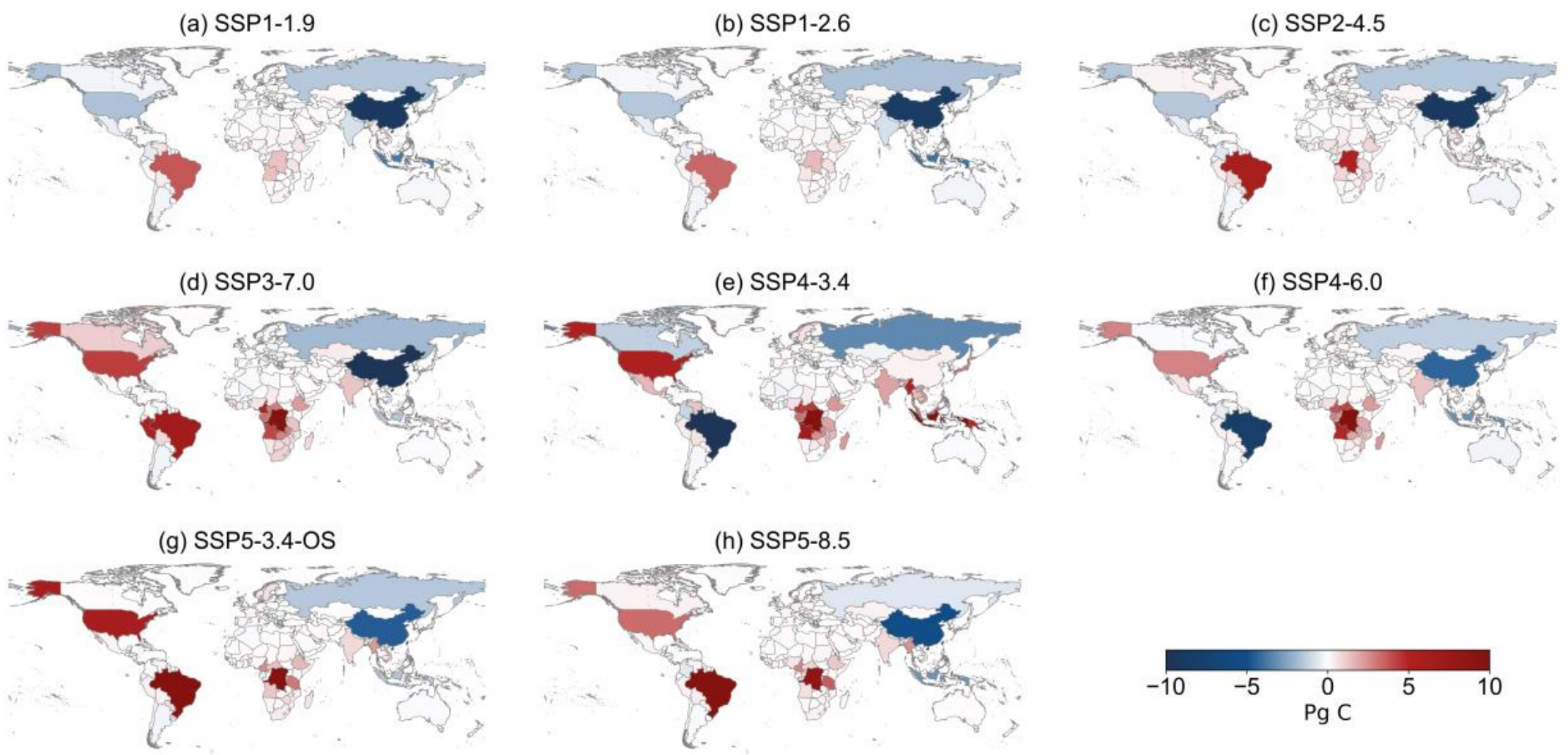

Fig. 3. Regional cumulative E_{LUC} from 2024 to 2100 under shifted SSP LULCC scenarios driven by the low-warming climate and atmospheric CO_2 forcing.

156: Reference methods somewhere

Response:

We have added a reference to the Methods section where the scenario design is described in detail.

164-165: This sentence feels out of context. It should either be removed or more thoroughly explained.

Response:

The sentence has been removed.

168: “showing the importance of early, stringent land-use regulation” Why does it

show that? These numbers are only ~25% different, despite taking 3x longer to reach the goal

Response:

Thank you for this insightful comment. Our intention to emphasize that the importance of early and stringent land-use regulation lies in avoiding irreversible emissions in the early decades and bringing forward the transition to net removals. The difference in cumulative removals between Gross2030 and Gross2050 (~25%) reflects emissions that occur early in the century and cannot be compensated later, even under equally stringent policies.

We have revised the text to clarify that policy stringency (gross-zero versus net-zero deforestation) is the dominant control on long-term cumulative land-use carbon outcomes, while earlier implementation enhances mitigation outcomes by reducing avoidable near-term emissions and advancing the onset of net removal.

The following is the associated revision in the Results section (see lines 170-178).

Both Gross2030 and Gross2050 scenarios change into strong removals, following trajectories similar to Commit but with delayed peaks (**Fig. 5**f-g). Their cumulative removals reach −30 Pg C and −23 Pg C (Supplementary Table 2). It reflects that delaying the cessation of gross deforestation allows additional emissions to occur in the first decades. By contrast, the Net2030 and Net2050 scenarios permit continued gross deforestation balanced by afforestation, while following the same BAU2030 LULCC trajectory as the Gross scenarios for all non-targeted activities (**Fig. 5**h-i). The resulting cumulative fluxes are limited to 4 Pg C and 9 Pg C, respectively. These results indicate that policy stringency is the primary determinant of long-term land-use carbon benefits, while earlier implementation further enhances cumulative removals by shortening the period of avoidable emissions. Net-zero deforestation targets cannot replicate the mitigation potential of gross-zero strategies, even when implemented over shorter timescales.

170-171: I like the point the authors are trying to make about offset-based policies, but I think the wording could use adjustment. Theoretically, offset policy could have afforestation increase past 2030. If I understand extended data figure 2e1 correctly, then afforestation is assumed to decline much more steeply after 2030 than current trajectories would suggest.

Response:

We agree that net-zero deforestation policies in the real world could involve a post-2030 acceleration of afforestation. The scenarios analyzed here are designed as counterfactual experiments rather than predictions, and both gross- and net-based are constructed under the same BAU2030 land-use trajectory. This unified constraint ensures internal consistency and comparability across scenarios. We have revised the Results text to clarify that our conclusions pertain to the relative effectiveness of gross- versus net-zero forest policies under comparable land-use constraints.

The following is the associated revision in the Results section (see lines 170-178).

Both Gross2030 and Gross2050 scenarios change into strong removals, following trajectories similar to Commit but with delayed peaks (**Fig. 5**f-g). Their cumulative removals reach −30 Pg C and −23 Pg C (Supplementary Table 2). It reflects that delaying the cessation of gross deforestation allows additional emissions to occur in the first decades. By contrast, the Net2030 and Net2050 scenarios permit continued gross deforestation balanced by afforestation, while following the same BAU2030 LULCC trajectory as the Gross scenarios for all non-targeted activities (**Fig. 5**h-i). The resulting cumulative fluxes are limited to 4 Pg C and 9 Pg C, respectively. These results indicate that policy stringency is the primary determinant of long-term land-use carbon benefits, while earlier implementation further enhances cumulative removals by shortening the period of avoidable emissions. Net-zero deforestation targets cannot replicate the mitigation potential of gross-zero strategies, even when implemented over shorter timescales.

Fig 4: I generally like this figure and find it helpful, but all of the numbers in the text reference cumulative C. If the authors think cumulative carbon is the most relevant metric that should be compared between scenarios, it might make sense to make a figure that removes the time dimension - for example a stacked bar chart with experiment on the x and cumulative PgC on the y.

Response:

We have revised the figures to explicitly include panels showing globally cumulative E_{LUC} and its components over 2024–2100. Fig. 2k now shows the globally cumulative E_{LUC} components under each shifted SSP scenario. Fig. 4j shows the corresponding cumulative component breakdown under each idealized scenario.

The following is the associated revision in the Results section (see page 5).

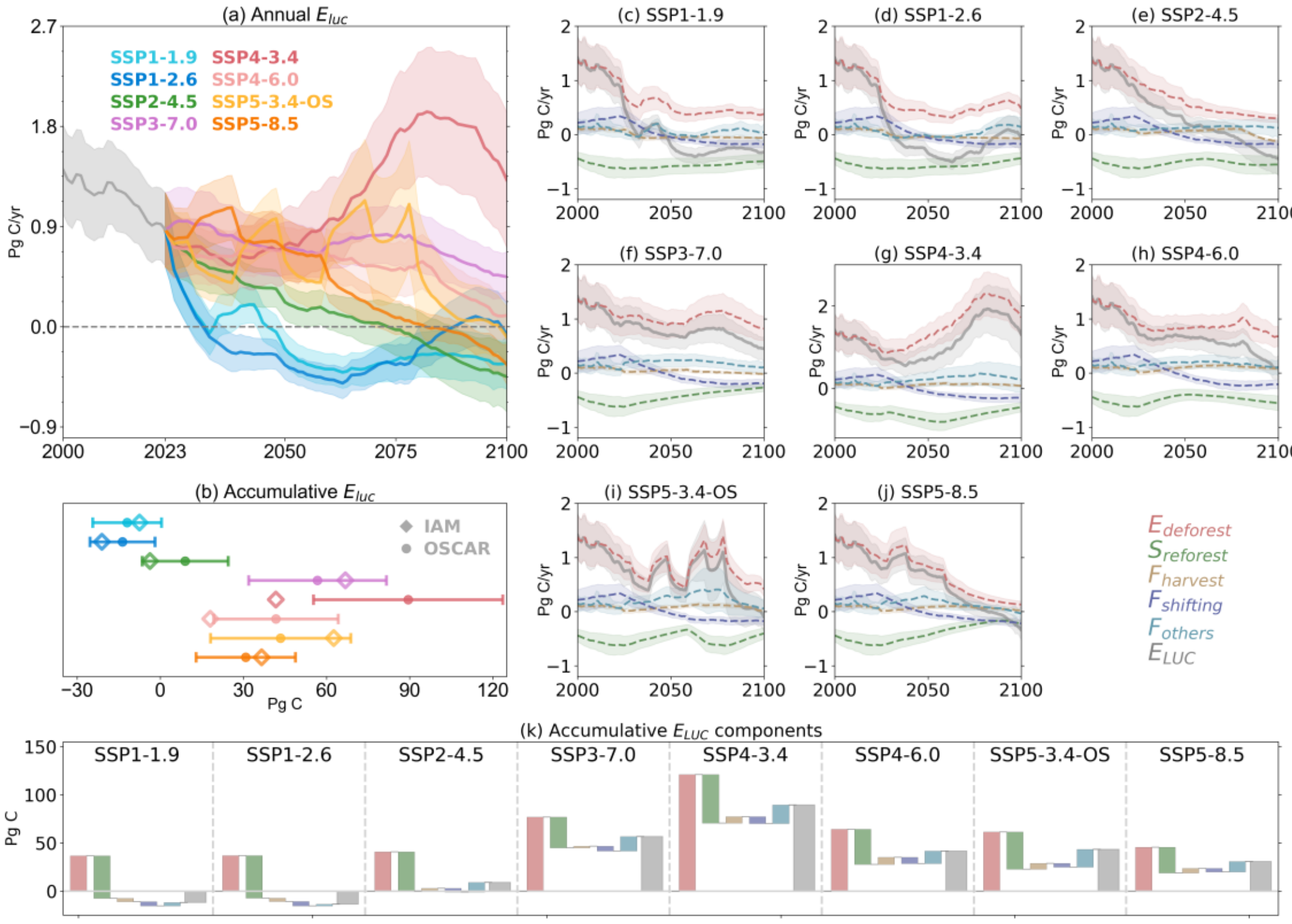

Fig. 4. Global E_{LUC} under shifted SSP scenarios driven by the low-warming climate and atmospheric CO_2 forcing. (a) Annually global E_{LUC} under shifted SSP scenarios in 2000-2100. (b) Accumulative global E_{LUC} under shifted SSP scenarios in 2024-2100. The error bars represent the accumulative global E_{LUC} under shifted SSP scenarios during the period 2024-2100 in this study. The diamond markers indicate the accumulative global E_{LUC} simulated by the IAMs over the native scenario period (2015–2091), used here for reference. (c-j) Global E_{LUC} components from 2024 to 2100 under different SSP scenarios. (k) Globally accumulative E_{LUC} components in 2024-2100 under each SSP scenario. Exact cumulative values for each ELUC component and scenario are reported in Supplementary Table 2.

The following is the associated revision in the Results section (see page 7).

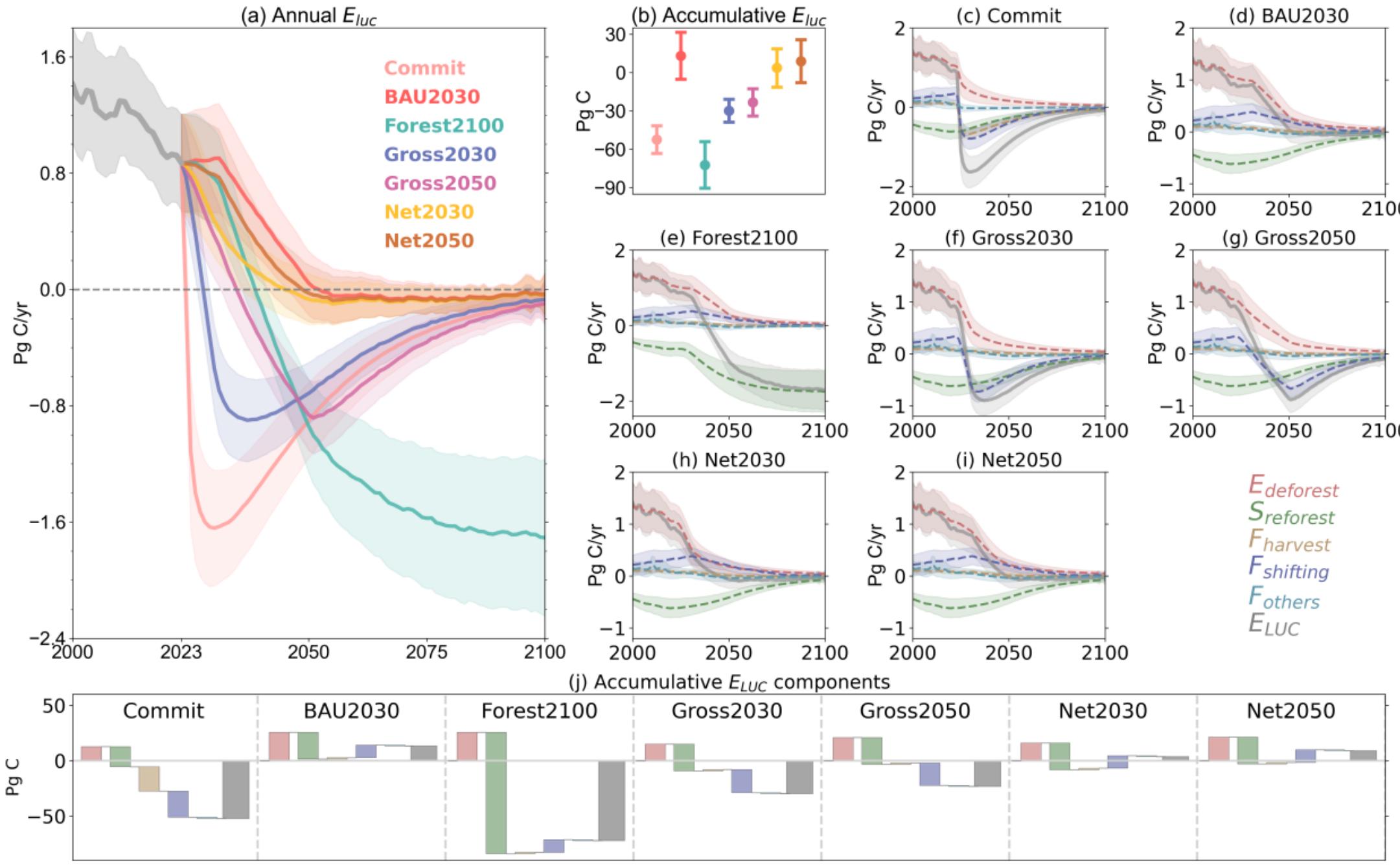

Fig. 5. Global E_{LUC} under idealized scenarios driven by the low-warming climate and atmospheric CO_2 forcing. (a) Annually global E_{LUC} under idealized scenarios in 2000-2100. (b) Accumulative global E_{LUC} under

idealized scenarios in Gray lines represent historical values from 2000 to 2023. (c-i) Global E_{LUC} components from 2024 to 2100 under idealized scenarios. (j) Globally accumulative E_{LUC} components in 2024-2100 under each idealized scenario. Exact cumulative values for each ELUC component and scenario are reported in Supplementary Table 2.

219-220: This reads like a result from this paper but I don't see this analysis and it cites a different paper

Response:

Our intention was to provide a synthesis and interpretation of the component-level results from Figs. 2 and 4 in the context of existing literature. In the revised manuscript, we clarify that the statement refers to the dominant contribution of deforestation and forest regrowth to total E_{LUC} as evidenced by the cumulative component magnitudes reported in this study.

The following is the associated revision in the Discussion section (see lines 214-217).

The cumulative magnitudes of deforestation emissions and forest regrowth removals substantially exceed those of other land-use components (Fig. 2,4), indicating that these two processes dominate the global land-use carbon balance, which is consistent with previous assessments (Pan et al., 2024).

254: “upper bound” relates to my major comment. There are multiple countries (CAN, IDN) in Extended Figure 3 that have larger forest area than the afforestation scenario. Also Moustakis is referenced as the methodology, if I understood the methods correctly, alternations have been made to those forest areas.

Response:

Upper bound does not refer to a country-level maximum forest extent. The Forest2100 scenario uses the global map of maximum afforestation potential from Moustakis et al. (2024) to calculate the potential maximum forest area in each region. The AR6-based upper bound in Moustakis et al. (2024) is derived from a percentile of pooled global and regional afforestation changes across IAM scenarios, rather than from a single internally consistent scenario, and therefore does not impose hard spatial limits at the regional scale. Some SSP pathways involve stronger agricultural intensification, dietary change, or land abandonment, which locally relax land-competition constraints and allow for higher afforestation in certain regions.

The inconsistency arises from methodological differences between the Forest2100 scenario and SSP scenarios, so regional afforestation rates under SSP-specific land-use pathways are possible to exceed those implied by the Moustakis et al. (2024).

The following is the associated revision in the Discussion section (see lines 247-251).

The optimistic afforestation potential from Moustakis et al. (2024) used here should be interpreted as

the globally ambitious afforestation effort inferred from the Sixth Assessment Report (AR6) Scenarios Database; realistic outcomes will depend on biophysical, socio-economic, and ecological constraints that may reduce available land and sequestration efficiency (Griscom et al., 2017; Strassburg et al., 2020).

277: F_LASC is not defined

Response:

We have added an explicit definition of F_{LASC} at its first mention in the Methods section and clarified its meaning in the text.

The following is the associated revision in the Methods section (see lines 287-293).

where $S_{land,PI}$ represents the natural land sink under preindustrial land cover and F_{LASC} refers to the loss of additional sink capacity (LASC) due to land use changes. OSCAR characterizes F_{LASC} as the difference between the carbon uptake capacity of natural vegetation under preindustrial land cover and that under actual evolving land cover (Gasser and Ciais, 2013). This formulation of LASC corresponds to what has recently been termed Replaced Sources and Sinks (RSS) in the GCB-related paper (Friedlingstein et al., 2026). By contrast, Dorgeist et al. (2024) define LASC as a residual (i.e., synergistic) term within a factorial experimental design, which aggregates the loss of sink capacity with an interaction component that is treated separately as part of E_{LUC} in our framework.

307: "provided to TRENDY DGVMs as a best-guess annual…" there is no context for this statement. The acronym isn't defined and the purpose of this is not stated

Response:

We have clarified the description to explicitly state that the atmospheric CO_2 concentration time series corresponds to the best-estimate global annual CO_2 concentrations provided by the GCB assessment available in year N, and that the forcing covers the period 1701 to year N−1, consistent with the TRENDY protocol.

The following is the associated revision in the Methods section (see lines 321-323).

In offline land-only mode, OSCAR requires inputs of atmospheric CO_2, regional climate, and regional LULCC. Atmospheric CO_2 concentration comes from the GCB2024, provided to TRENDY models as a best-guess annual and global time series covering from 1701 to 2023.

324: sigma is the standard deviation of what?

Response:

σ is the standard deviation of the constraint. The text has been revised accordingly.

The following is the associated revision in the Methods section (see lines 337-342).

This weight is calculated as the following equation assuming a Gaussian distribution of the posterior uncertainty:

$$w(x) = \frac{1}{\sigma\sqrt{2\pi}} exp\left(-\frac{(x-\mu)^2}{2\sigma^2}\right) \tag{8}$$

where μ is the average of the constraint, σ is the corresponding standard deviation, and x is the value of each simulation of the ensemble. The constraint is the estimate of $S_{land,actual}$ over the instrumental observation period (from 1960 onwards), provided by the GCB2023, which is currently 150±35 Pg C over 1960-2022 (Friedlingstein et al., 2023). The ensemble is evaluated over the same period to ensure consistency between the simulated and observationally constrained quantities.

326: Is the ensemble calculated over the same period? Is 30 the standard deviation?

Response:

Yes. The ensemble is calculated over the same period (1960–2022), and the value of ±35 Pg C corresponds to the one-standard-deviation of the GCB2023 estimate.

The following is the associated revision in the Methods section (see lines 340-341).

The constraint is the estimate of $S_{land,actual}$ over the instrumental observation period (from 1960 onwards), provided by the GCB2023, which is currently 150±35 Pg C over 1960-2022 (Friedlingstein et al., 2023).

335: Here N is 2024 and in the previous section N-1 was 2022.

Response:

We have revised the text to ensure consistent use of explicit years throughout. In the revised version, N corresponds uniformly to 2024, historical forcings extend to 2023, and no references remain where N−1 is ambiguously interpreted as 2022.

338: "which follows a summary of the setup used with OSCAR for the GCB of year N" I don't know what this means

Response:

We have revised the sentence to explicitly describe the historical initialization procedure and treatment of land-use legacy effects. The revised text now states how historical land-use transitions are prescribed, and how carbon pools are initialized.

The following is the associated revision in the Methods section (see lines 352-354).

Since E_{LUC} of a given year depends on the LULCC of previous years (the so-called legacy effect), we initialize OSCAR with prescribed historical land-use transitions and corresponding carbon pool states, consistent with the standard OSCAR configuration used for the GCB2024 (Gasser et al., 2020).

371: Broken links

Response:

We have removed the broken link.

Extended data Figure 3: The x axis is unclear. If the bands correspond to a year, why are they different widths? And why are the markers for the different lines in different locations on the x axis? Some of the colors are very difficult to tell apart. The use of markers helps, but the markers are not in the legend.

Response:

We thank the reviewer for this detailed comment. The Supplementary Fig. 3 was designed to minimize visual overlap among scenarios, as total forest area trajectories across scenarios are close, leading to substantial overplotting as simple line plots. To make it clear, we introduced small horizontal offsets and variable band widths. However, given the large number of scenarios, this approach still resulted in visual ambiguity. To address these issues, we have replaced Supplementary Fig. 3 with a data table that reports total forest area by region (row) and scenario (column).

The following is the associated revision in the Supplementary Information.

Supplementary Table 4. Forest area (Mha) in 2023 and 2100 under different scenarios. Regions include global, Brazil (BRA), Canada (CAN), China (CHN), Democratic Republic of the Congo (COD), European Union (EU), Indonesia (IDN), Russia (RUA), and the United States of America (USA).

Scenario (year)	Global	BRA	CAN	CHN	COD	EU	IDN	RUS	USA
Historical (2023)	3914.2	471.0	445.6	227.6	135.1	149.3	102.1	833.2	303.9
SSP1-1.9 (2100)	3990.6	473.2	448.3	266.9	131.4	153.7	107.5	834.1	318.4
SSP1-2.6 (2100)	3967.1	468.9	447.8	261.2	131.4	151.5	107.2	834.5	311.0
SSP2-4.5 (2100)	3968.8	488.7	439.9	262.9	127.6	152.0	90.7	840.9	331.9
SSP3-7.0 (2100)	3593.1	445.0	430.9	287.2	13.9	145.4	96.4	840.3	266.0
SSP4-3.4 (2100)	3449.8	564.0	450.1	214.8	16.6	153.9	25.7	846.3	261.5
SSP4-6.0 (2100)	3765.4	534.1	451.4	248.6	32.6	153.0	106.9	852.1	295.1
SSP5-3.4-OS (2100)	3779.0	453.2	443.6	255.1	95.6	147.2	99.9	829.5	274.0
SSP5-8.5 (2100)	3789.6	451.3	444.5	241.9	84.0	146.4	114.3	834.5	282.7
BAU2030 (2100)	3842.7	435.6	446.5	242.8	125.6	152.9	100.2	833.6	298.0
Commit (2100)	3910.5	469.4	445.6	228.9	134.5	149.4	101.8	833.1	303.5
Forest2100 (2100)	4849.1	585.2	450.2	287.0	154.8	173.1	106.6	878.1	390.8
Gross2030 (2100)	3941.9	465.3	446.8	247.1	132.8	153.8	101.5	833.9	302.8
Gross2050 (2100)	3885.7	451.0	446.6	244.4	126.6	153.5	101.0	833.7	299.3
Net2030 (2100)	3933.8	464.0	446.5	245.6	132.8	153.2	101.6	833.8	302.6
Net2050 (2100)	3883.2	448.3	446.5	244.2	128.7	153.0	100.7	833.7	300.1

Reference

Dorgeist, L., Schwingshackl, C., Bultan, S., and Pongratz, J.: A consistent budgeting of terrestrial carbon fluxes, Nature Communications, 15, 7426, http://doi.org/10.1038/s41467-024-51126-x, 2024.

Friedlingstein, P., Le Quéré, C., O'Sullivan, M., Hauck, J., Landschützer, P., Luijkx, I. T., Li, H., van der Woude, A., Schwingshackl, C., Pongratz, J., Regnier, P., Andrew, R. M., Bakker, D. C. E., Canadell, J. G., Ciais, P., Gasser, T., Jones, M. W., Lan, X., Morgan, E., Olsen, A., Peters, G. P., Peters, W., Sitch, S., and Tian, H.: Emerging climate impact on carbon sinks in a consolidated carbon budget, Nature, 649, 98-103, 10.1038/s41586-025-09802-5, 2026.

Friedlingstein, P., O'Sullivan, M., Jones, M. W., Andrew, R. M., Bakker, D. C. E., Hauck, J., Landschützer, P., Le Quéré, C., Luijkx, I. T., Peters, G. P., Peters, W., Pongratz, J., Schwingshackl, C., Sitch, S., Canadell, J. G., Ciais, P., Jackson, R. B., Alin, S. R., Anthoni, P., Barbero, L., Bates, N. R., Becker, M., Bellouin, N., Decharme, B., Bopp, L., Brasika, I. B. M., Cadule, P., Chamberlain, M. A., Chandra, N., Chau, T. T. T., Chevallier, F., Chini, L. P., Cronin, M., Dou, X., Enyo, K., Evans, W., Falk, S., Feely, R. A., Feng, L., Ford, D. J., Gasser, T., Ghattas, J., Gkritzalis, T., Grassi, G., Gregor, L., Gruber, N., Gürses, Ö., Harris, I., Hefner, M., Heinke, J., Houghton, R. A., Hurtt, G. C., Iida, Y., Ilyina, T., Jacobson, A. R., Jain, A., Jarníková, T., Jersild, A., Jiang, F., Jin, Z., Joos, F., Kato, E., Keeling, R. F., Kennedy, D., Klein Goldewijk, K., Knauer, J., Korsbakken, J. I., Körtzinger, A., Lan, X., Lefèvre, N., Li, H., Liu, J., Liu, Z., Ma, L., Marland, G., Mayot, N., McGuire, P. C., McKinley, G. A., Meyer, G., Morgan, E. J., Munro, D. R., Nakaoka, S. I., Niwa, Y., O'Brien, K. M., Olsen, A., Omar, A. M., Ono, T., Paulsen, M., Pierrot, D., Pocock, K., Poulter, B., Powis, C. M., Rehder, G., Resplandy, L., Robertson, E., Rödenbeck, C., Rosan, T. M., Schwinger, J., Séférian, R., Smallman, T. L., Smith, S. M., Sospedra-Alfonso, R., Sun, Q., Sutton, A. J., Sweeney, C., Takao, S., Tans, P. P., Tian, H., Tilbrook, B., Tsujino, H., Tubiello, F., van der Werf, G. R., van Ooijen, E., Wanninkhof, R., Watanabe, M., Wimart-Rousseau, C., Yang, D., Yang, X., Yuan, W., Yue, X., Zaehle, S., Zeng, J., and Zheng, B.: Global Carbon Budget 2023, Earth Syst. Sci. Data, 15, 5301-5369, http://doi.org/10.5194/essd-15-5301-2023, 2023.

Gasser, T. and Ciais, P.: A theoretical framework for the net land-to-atmosphere CO₂ flux and its implications in the definition of "emissions from land-use change", Earth Syst. Dynam., 4, 171-186, http://doi.org/10.5194/esd-4-171-2013, 2013.

Gasser, T., Ciais, P., and Lewis, S. L.: How the Glasgow Declaration on Forests can help keep alive the 1.5 °C target, Proceedings of the National Academy of Sciences, 119, e2200519119, https://doi.org/10.1073/pnas.2200519119, 2022.

Gasser, T., Crepin, L., Quilcaille, Y., Houghton, R. A., Ciais, P., and Obersteiner, M.: Historical CO2 emissions from land use and land cover change and their uncertainty, Biogeosciences, 17, 4075-4101, https://doi.org/10.5194/bg-17-4075-2020, 2020.

Griscom, B. W., Adams, J., Ellis, P. W., Houghton, R. A., Lomax, G., Miteva, D. A., Schlesinger, W. H., Shoch, D., Siikamäki, J. V., Smith, P., Woodbury, P., Zganjar, C., Blackman, A., Campari, J., Conant, R. T., Delgado, C., Elias, P., Gopalakrishna, T., Hamsik, M. R., Herrero, M., Kiesecker, J., Landis, E., Laestadius, L., Leavitt, S. M., Minnemeyer, S., Polasky, S., Potapov, P., Putz, F. E., Sanderman, J., Silvius, M., Wollenberg, E., and Fargione, J.: Natural climate solutions, Proceedings of the National Academy of Sciences, 114, 11645-11650, http://doi.org/10.1073/pnas.1710465114, 2017.

Hurtt, G. C., Chini, L., Sahajpal, R., Frolking, S., Bodirsky, B. L., Calvin, K., Doelman, J. C., Fisk, J., Fujimori, S., Klein Goldewijk, K., Hasegawa, T., Havlik, P., Heinimann, A., Humpenöder, F., Jungclaus, J., Kaplan, J. O., Kennedy, J., Krisztin, T., Lawrence, D., Lawrence, P., Ma, L., Mertz, O., Pongratz, J.,

Popp, A., Poulter, B., Riahi, K., Shevliakova, E., Stehfest, E., Thornton, P., Tubiello, F. N., van Vuuren, D. P., and Zhang, X.: Harmonization of global land use change and management for the period 850–2100 (LUH2) for CMIP6, Geosci. Model Dev., 13, 5425-5464, https://doi.org/10.5194/gmd-13-5425-2020, 2020.
Moustakis, Y., Nützel, T., Wey, H.-W., Bao, W., and Pongratz, J.: Temperature overshoot responses to ambitious forestation in an Earth System Model, Nature Communications, 15, 8235, https://doi.org/10.1038/s41467-024-52508-x, 2024.
Pan, Y., Birdsey, R. A., Phillips, O. L., Houghton, R. A., Fang, J., Kauppi, P. E., Keith, H., Kurz, W. A., Ito, A., Lewis, S. L., Nabuurs, G.-J., Shvidenko, A., Hashimoto, S., Lerink, B., Schepaschenko, D., Castanho, A., and Murdiyarso, D.: The enduring world forest carbon sink, Nature, 631, 563-569, https://doi.org/10.1038/s41586-024-07602-x, 2024.
Strassburg, B. B. N., Iribarrem, A., Beyer, H. L., Cordeiro, C. L., Crouzeilles, R., Jakovac, C. C., Braga Junqueira, A., Lacerda, E., Latawiec, A. E., Balmford, A., Brooks, T. M., Butchart, S. H. M., Chazdon, R. L., Erb, K.-H., Brancalion, P., Buchanan, G., Cooper, D., Díaz, S., Donald, P. F., Kapos, V., Leclère, D., Miles, L., Obersteiner, M., Plutzar, C., de M. Scaramuzza, C. A., Scarano, F. R., and Visconti, P.: Global priority areas for ecosystem restoration, Nature, 586, 724-729, https://doi.org/10.1038/s41586-020-2784-9, 2020.

Reviewer #3 (Remarks to the Author):

Summary

The authors estimate land use change emissions with a land model under several future scenarios to assess the potential impacts of different land change strategies on emissions. Two CMIP6 scenarios put these estimates in the context of other global assessments and several idealized scenarios provide insights to the potential effects of particular approaches. They show that halting deforestation is the most effective approach for reducing emissions, with additional afforestation providing further benefits in the form of carbon sequestration.

They also provide these emission data across all their scenarios at the country level for others to use.

Overall response

This is an interesting and useful paper both in terms of its results and its demonstration of how a land model can track these land fluxes in a detailed manner. I have only one major reservation with the method, and some suggestions for improving clarity.

Response:

We thank the reviewer for this positive overall assessment of the manuscript. We have carefully addressed the major methodological concern and have revised the manuscript to improve clarity according in response to the reviewer's suggestions.

1) The application of SSP LULCC forcings appears to create a major discontinuity at the beginning of 2024 (due to 'shifting' the scenarios). This could be because it isn't explained adequately, but conceptually it does not seem to align withe the extrapolation that was used to extend the historical simulation through 2023.

Response:

We would like to clarify that the application of shifted SSP LULCC forcings does not introduce a major numerical discontinuity at the start of 2024.

We apply the shift to LUH2 transition data (i.e., annual land-use changes) rather than annual land-use states. Historical land-use states are simulated continuously up to 2023, and the shifted SSP transitions are then applied from 2024 onward. As a result, the land-use state at the end of 2023 is preserved, and no artificial jump in land cover occurs at the transition to the SSP-based forcings. We have revised the Methods section to clarify this distinction and to explicitly state the use of transition data, thereby addressing the concern about continuity at the beginning of 2024.

It should also be noted that the discontinuity between historical period and scenarios happens even when the SSPs are not shifted, as illustrated in Fig. R1.

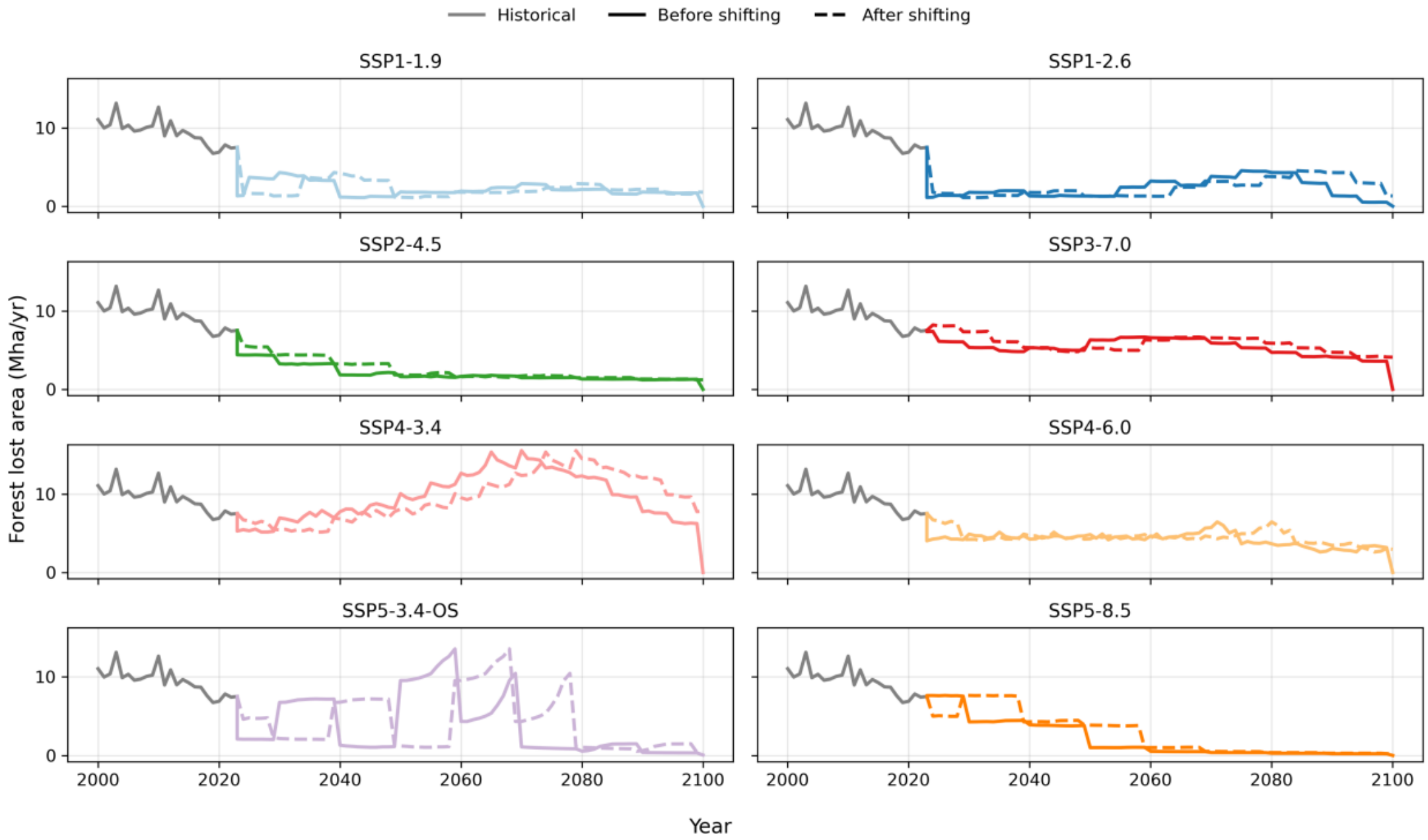

Fig. R1. Illustrative comparison of forest loss trajectories before and after temporal shifting

The extension after 2023 and the shifted SSP approach serve different purposes. The extrapolation of recent trends (2013-2023) is used solely to generate our idealized scenarios. In contrast, the shifted SSP scenarios are not an extrapolation of recent trends, but a means of introducing IAM-derived future land-use dynamics after the historical period..

The following is the associated revision in the Methods section (see lines 376-392).

The LULCC data under eight SSP scenarios are taken from the LUH2 dataset for CMIP6 (Hurtt et al., 2020), representing internally consistent socio-economic pathways generated by IAMs rather than predictions tied to specific calendar years.

The LUH2 transition data under SSP scenarios are provided from 2015 onwards, whereas our simulations must start in 2024, immediately after the end of the observation-constrained historical period used in the GCB2024. To ensure continuity at the transition from historical land-use conditions, while preserving the internally consistent dynamics produced by IAMs, we apply a time shift to the SSP LULCC transition data. These methods leverage the SSP-derived annual LULCC rates starting from the 2023 observational baseline.

The first 77 years of each original SSP scenario (2015–2091) are reassigned 2024–2100. Historical LCC is simulated up to 2023, and the shifted SSP LCC data are then appended from 2024 onward. For LU components (wood harvest and shifting cultivation), an offset is applied using the mean of the last 11 years (2013–2023) as a reference, consistent with the treatment of CO_2 and climate forcings.

We recognize that this means the new LULCC data is inconsistent with other aspects of these scenarios (e.g., non-CO_2 emissions, population). Still, it is difficult to maintain consistency across all aspects while changing the starting year of a scenario. This approach preserves the rates of change, temporal evolution, and spatial patterns of land-use change embedded in each SSP narrative under IAM-based projections. Cumulative LULCC emissions from IAMs are taken over their native scenario period

(2015–2091) and used as a benchmark to contextualize OSCAR-based estimates under the corresponding shifted SSP land-use pathways, without serving as model inputs or constraints.

The following is the associated revision in the Discussion section (see lines 257-264).

SSP LULCC trajectories are temporally shifted to ensure continuity with the observation-constrained historical period, which preserves their internal dynamics but breaks full consistency with other SSP dimensions (e.g., population or non-CO_2 emissions). These scenarios should therefore be interpreted as stylized post-historical land-use pathways rather than calendar-year-specific policy projections. A key constraint in designing idealised scenarios is that indefinitely extrapolating recent land-use trends or holding present-day land-use change rates constant over multiple decades leads to non-physical outcomes in many regions, including negative land areas, land-use transitions exceeding national land availability, or highly unrealistic land-use compositions. The shifted SSP scenarios therefore combine near-term continuation of recent trends with long-term constraints to ensure physical plausibility.

2) Some things need more explanation in the results/discussion if the methods section will be at the end of the paper. Otherwise the paper is difficult to comprehend if the reader has not read the methods section first. For example, referring to the extended data table and figures when introducing the corresponding info would be helpful.

Response:

We thank the reviewer for this helpful comment. We have added brief explanatory sentences at the first occurrence of the shifted SSP scenarios and the idealized scenarios in the Results section, referring to the corresponding Supplementary figures and tables. We have also added a concise overview of the modeling framework and scenario structure in the Discussion.

The following is the associated revision in the Results section (see lines 104-108).

The shifted SSP scenarios under low warming represent internally consistent LULCC trajectories derived from Integrated Assessment Models (IAMs), in which the original SSP land-use time series (starting in 2015) are temporally realigned with the observation-constrained historical period ending in 2023. These scenarios provide a benchmark for comparing OSCAR-based land-use carbon fluxes with IAM-based expectations under contrasting socio-economic pathways. Resulting forest-area trajectories are summarized in Supplementary Table 4.

The following is the associated revision in the Methods section (see lines 155-161).

In addition to the shifted SSP pathways, we designed seven idealized scenarios to explore alternative LULCC pathways (Supplementary Fig. 2). The Commit scenario ceases all LULCC to isolate legacy carbon dynamics, while BAU2030 extends current trends to 2030 and phases out land-cover change by 2050. Forest2100 intensifies afforestation on pasture to reach 935 Mha of forest by 2100. The Gross2030 and Gross2050 scenarios halt gross deforestation by 2030 or 2050, and Net2030 and Net2050 balance deforestation with afforestation by those years, all relative to BAU2030. These scenarios isolate the effects of policy stringency and implementation timing on land-use carbon fluxes by imposing simplified, physically consistent constraints on forest loss and gain.

The following is the associated revision in the Discussion section (see lines 209-210).

This study quantifies future LULCC carbon fluxes with a suite of shifted SSP and idealized scenarios.

The results discussed below primarily focus on E_{LUC} under a fixed low-warming climate pathway (see Methods and Supplementary Tables 2–4).

3) There are some grammatical errors scattered throughout the paper that should be addressed.

Response:

We have carefully proofread the entire manuscript and corrected grammatical errors and minor language issues throughout the text to improve clarity and readability.

Specific comments and suggestions

Abstract

Lines 16-17

It is a little confusing to refer to the same carbon flux process as 'removals' and 'gains, and also have one number negative and the other positive. Please make these two consistent.

Response:

We have revised the abstract to use a single, consistent terminology for land carbon uptake and to report all values with a consistent sign convention. We clarify that negative values correspond to carbon removals ($E_{LUC} < 0$), whereas positive values indicate continued emissions from the surface to the atmosphere ($E_{LUC} > 0$).

The following is the associated revision in the Abstract section (see lines 16-17).

Early cessation of gross deforestation yields large and persistent removals (−30 Pg C by 2100 if achieved by 2030), whereas forest area net-balance strategies still result in emissions of 4–9 Pg C.

The following is the associated revision in the Methods section (see lines 296-297)

$$E_{LUC} = E_{LCC} + E_{LU} \quad (3)$$

where negative values of E_{LUC} correspond to carbon removals from the atmosphere ($E_{LUC} < 0$), whereas positive values indicate carbon emissions associated with LULCC ($E_{LUC} > 0$).

Lines 119-147

The listing and parenthetical numbers become cumbersome here. Please put these numbers in a table. They will be easier to digest and compare.

Response:

Following this advice, we have moved the detailed cumulative contributions of

LULCC into new tables (Supplementary Table 2-3). The Results section has been revised to focus on the dominant processes, contrasts among scenarios, and underlying mechanisms, while referring readers to the table for quantitative details.

The following is the associated revision in the Supplementary Information.

Supplementary Table 2. Cumulative contributions of E_{LUC} components across LULCC scenarios under low warming in 2024-2100 (Unit: Pg C).

Scenario	$E_{deforest}$	$S_{reforest}$	$E_{harvest} + S_{harvest}$	$E_{shifting} + S_{shifting}$	F_{others}	E_{LUC}
SSP1-1.9	36.7	-44.1	-3.4	-4.4	3.4	-12.0
SSP1-2.6	36.9	-44.2	-3.5	-4.6	1.7	-13.6
SSP2-4.5	40.9	-40.8	2.8	-3.9	10.0	9.0
SSP3-7.0	77.0	-32.1	1.9	-5.0	15.0	56.7
SSP4-3.4	121.1	-50.4	6.6	-7.2	19.4	89.4
SSP4-6.0	64.3	-36.6	7.4	-6.4	13.0	41.7
SSP5-3.4-OS	61.5	-38.6	6.1	-4.0	18.5	43.4
SSP5-8.5	45.7	-26.9	4.8	-3.7	11.0	30.8
Commit	12.7	-18.2	-22.0	-23.6	-1.2	-52.3
BAU2030	25.5	-24.1	1.3	11.4	-0.8	13.2
Forest2100	25.5	-109.6	1.3	11.4	-0.7	-72.2
Gross2030	14.9	-24.1	1.2	-20.9	-0.8	-29.7
Gross2050	20.8	-24.1	1.2	-20.4	-0.8	-23.2
Net2030	15.9	-24.1	1.3	11.4	-0.8	3.7
Net2050	21.2	-24.1	1.3	11.4	-0.8	9.0

Supplementary Table 3. Cumulative regional contributions of E_{LUC} across LULCC scenarios under low warming in 2024-2100 (Unit: Pg C). Regions include China (CHN), Indonesia (IDN), Brazil (BRA), Democratic Republic of the Congo (COD), United States (USA), Russia (RUS), India (IND), Angola (AGO), Peru (PER), Tanzania (TZA), and Colombia (COL).

Scenario	CHN	IDN	BRA	COD	USA	RUS	IND	AGO	PER	TZA	COL
SSP1-1.9	-8.9	-3.8	3.8	1.4	-1.7	-1.5	-0.8	1.4	0.2	0.6	-0.5
SSP1-2.6	-8.6	-4.0	3.4	1.5	-1.6	-1.7	-0.8	0.5	0.2	0.7	-0.4
SSP2-4.5	-9.0	0.5	6.0	5.2	-1.5	-1.5	-0.2	0.9	0.4	0.8	-0.6
SSP3-7.0	-12.7	-1.5	6.8	19.6	4.3	-1.9	1.3	4.2	5.5	1.3	0.0
SSP4-3.4	0.1	12.7	-18.4	22.8	5.4	-3.3	2.1	6.5	0.4	2.1	-1.1
SSP4-6.0	-4.3	-3.1	-8.2	19.5	2.8	-1.3	1.3	4.9	0.3	1.9	-0.5
SSP5-3.4-OS	-4.7	-1.6	11.6	9.7	6.1	-1.5	0.8	1.2	0.3	3.1	0.0
SSP5-8.5	-5.3	-3.0	10.8	8.6	3.3	-0.7	0.8	0.7	0.3	3.6	-0.6
Commit	-7.2	-6.1	-4.8	-2.1	-2.7	-2.6	-3.2	0.0	0.1	0.0	-1.2
BAU2030	-6.3	2.6	8.1	3.1	0.5	-0.7	-0.4	0.9	0.8	0.6	-0.4
Forest2100	-11.1	1.8	-5.9	0.3	-7.2	-3.6	-1.1	-2.1	0.8	0.4	-1.4
Gross2030	-7.1	-4.4	-2.0	-1.3	-0.1	-0.7	-1.0	0.3	0.2	0.3	-1.6
Gross2050	-6.7	-4.2	-0.2	-0.7	0.3	-0.7	-0.9	0.5	0.4	0.4	-1.4
Net2030	-6.8	2.5	4.6	2.5	-0.1	-0.7	-0.4	0.5	0.5	0.5	-0.6
Net2050	-6.5	2.6	6.6	2.9	0.2	-0.7	-0.4	0.7	0.7	0.6	-0.5

The following is the associated revision in the Results section (see lines 130-150).

Across all scenarios, deforestation remains the dominant carbon source, while forest regrowth constitutes the primary removal (Fig. 2c–j; Supplementary Table 2). Contributions from wood harvest, shifting cultivation, and other land-use transitions are secondary, varying with regional management practices and policy stringency. Under SSP1 pathways, emissions from deforestation are largely compensated by substantial carbon uptake from forest regrowth, with additional smaller sinks arising from reduced wood harvest and shifting cultivation. As a result, LULCC emissions are close to balance or slightly negative over 2024-2100. Under SSP2-4.5, the narrowing balance between forest loss and regrowth combined with positive contributions from harvest and other transitions, leads to a small carbon source. Under SSP3-7.0, cumulative deforestation emissions increase sharply, while forest regrowth weakens, reinforcing a strong source. Similar but more extreme dynamics emerge under SSP4, reflecting divergent land-use governance across regions. In SSP4-3.4, extensive deforestation is only partly offset by regrowth, resulting in the largest cumulative source among all scenarios, whereas SSP4-6.0 yields smaller but still substantial emissions. Fossil-fuel-driven SSP5 pathways exhibit comparable balances between forest loss and regrowth, but sustain land-use emissions due to delayed recovery and continued harvest activities.

At the regional scale, sustainable SSP1 scenarios show China and Indonesia emerging as dominant land-use carbon sinks, with the additional smaller sinks in the United States, Russia, and India (Fig. 3a, b; Supplementary Table 3). Tropical regions such as Brazil, the Democratic Republic of the Congo (COD), and Angola remain persistent sources, indicating continued deforestation pressure even under sustainable development narratives. Under SSP2 and SSP3 (Fig. 3c, d), weakening land-use regulation leads to increasingly uneven spatial patterns, with Brazil and the COD becoming dominant global sources, while China maintains substantial sink capacity and Southeast Asia provides only partial compensation. Divergent outcomes are most pronounced under SSP4 (Fig. 3f, g), where the contrasting regional policy regimes produce simultaneous large sinks and sources. In SSP5 pathways (Fig. 3h), Brazil and the COD remain leading emitters, while China and Indonesia retain weakened but still positive sink roles.

Introduction

Lines 56-58

Please provide definitions of Eluc and Sland. The reader has not read the methods yet, but understanding these is central to understanding the paper. What processed are included/excluded from each?

Response:

We have added definitions in the Introduction, explicitly stating which processes are included and excluded in each term, and clarifying their consistency with the GCB bookkeeping framework.

The following is the associated revision in the Introduction section (see lines 59-64).

We focus on E_{LUC}, defined consistently with the bookkeeping framework of the GCB (Friedlingstein et al., 2025). Here, E_{LUC} represents carbon fluxes from LULCC. We nevertheless also report estimates of the land sink under pre-industrial land cover ($S_{land,PI}$) and the land sink under actual land cover ($S_{land,actual}$) to facilitate comparison with national inventories. By providing a systematic projection of possible futures, our analysis reveals how different national land-use pathways could contribute to the

global mitigation challenge. These insights offer a foundation for designing more equitable and forward-looking land-based strategies within a limited remaining global carbon budget.

Also, you present Sland only in the 'Overview' section, yet you call the Eluc fluxes 'net' several times in the following sections, which can be confusing. Be clear on where you discuss Sland and where you discuss only Eluc.

Response:

To address this, we have revised the manuscript by removing the term "net" when discussing E_{LUC} in the subsequent sections.

You should refer the ED table here.

Response:

We have revised this paragraph to explicitly refer to Supplementary Table 1 (Carbon flux definitions), which provides concise definitions of E_{LUC}, $S_{land,\ actual}$ and $S_{land,\ PI}$, and clarifies the processes included in each term.

Also, how do you segregate shifting cultivation to/from forest from deforestation/afforestation?

Response:

We identify shifting cultivation directly from the LUH2 transition matrices at the grid-cell level. In LUH2, reciprocal land-cover transitions occurring within the same grid cell and year are explicitly associated with shifting cultivation, whereas other land-use changes are unidirectional (Hurtt et al., 2020).

We isolate shifting cultivation by identifying reciprocal transitions between forest and non-forest land-cover types within each grid cell and year (e.g., forest to cropland paired with cropland to forest of equal area). These reciprocal transitions are summed and classified as shifting cultivation, while the remaining non-reciprocal transitions are attributed to deforestation or afforestation, as described in Gasser et al. (2020).

Overview of flux behavior

Shifted SSP scenarios

Please explain here what 'shifted' means. The reader has not yet read the methods, so they don't know what these are, and yet they are repeatedly referred to.

Response:

We have added a short clarification in the opening paragraph of the Shifted SSP scenarios results subsection.

The following is the associated revision in the Results section (see lines 104-108).

The shifted SSP scenarios under low warming represent internally consistent LULCC trajectories derived from Integrated Assessment Models (IAMs), in which the original SSP land-use time series (starting in 2015) are temporally realigned with the observation-constrained historical period ending in 2023. These scenarios provide a benchmark for comparing OSCAR-based land-use carbon fluxes with IAM-based expectations under contrasting socio-economic pathways. Resulting forest-area trajectories are summarized in Supplementary Table 4.

Are these different than the scenarios presented in the 'Overview' section?

Response:

No. The scenarios discussed in the Shifted SSP scenarios section are the same shifted SSP LULCC pathways that are introduced in the Overview section. In the Overview, these shifted SSP scenarios are combined with both low- and high-warming climate forcings to separate the effects of land-use decisions from those of climate change on the major land carbon fluxes. This comparison demonstrates that E_{LUC} is primarily controlled by LULCC trajectories and is only weakly sensitive to climate forcing.

Based on this result, all subsequent analyses, including the Shifted SSP scenarios section, focus explicitly on E_{LUC} and adopt the low-warming climate and CO_2 pathway as a fixed forcing. This choice allows a straightforward comparison of LULCC-driven emissions across socio-economic narratives and IAM-based land-use trajectories without confounding climate effects.

The following is the associated revision in the Results section (see lines 99-101).

Given its direct link to land-use policy and its limited sensitivity to climate feedbacks, we focus in the following sections on E_{LUC}. All subsequent analyses of shifted SSP and idealized LULCC scenarios are conducted under the low-warming climate and atmospheric CO_2 pathway.

Idealized scenarios

How is wood harvest adjusted in these scenarios? It seems to be different for different scenarios, and although you state that LULCC ceases, logging continues (even though it is Land Use).

Response:

We have clarified the treatment of wood harvest across all idealized scenarios in the Methods and explicitly distinguished between land cover change (LCC) and land use

(LU, including shifting cultivation and wood harvest). Wood harvest is set to zero only in the Commit scenario, where all LULCC activities cease after 2023. In all the other scenarios (BAU2030, Forest2100, Gross2030/2050, Net2030/2050), wood harvest follows the BAU2030 trajectory and is not directly affected by the forest-area targets, consistent with our intention to isolate the effects of alternative deforestation and afforestation pathways. We have revised the Methods section to make this treatment explicit and to avoid confusion regarding the use of "LULCC cessation."

The following is the associated revision in the Methods section (see lines 400-433).

Committed Emissions (Commit). The Commit scenario assumes an immediate cessation of all LULCC activities, resulting in all LCC and LU input data dropping to zero as of year 2023 inclusive. While unrealistic, this scenario is designed as a diagnostic experiment to isolate the legacy carbon dynamics arising from historical LULCC perturbations, under the two assumed climate scenarios (low- and high-warming).

Business As Usual (BAU2030). The Business As Usual (BAU2030) scenario represents a constrained continuation of current land-use dynamics in the absence of additional policy intervention, serving as the baseline for all idealized scenarios. All LULCC in each region from 2023 to 2030 are linearly extrapolated from the trends of the past 11 years (currently, 2013-2023). After that, LCC is assumed to linearly reach zero by 2050 and LU is held constant at 2030 levels. This transition is introduced to prevent non-physical outcomes over long time horizons, such as land-use changes exceeding available land area or unrealistic dominance of a single land-use type by 2100. The choice of 2030 as the transition year reflects its central role in the COP26 commitment to halt and reverse forest loss by 2030.

Intense Afforestation (Forest2100). The Forest2100 scenario assesses the potential carbon sinks that could result from ambitious afforestation policies. In this scenario, intensified afforestation on pasture land aims to reach a regionally constrained global maximum forest area (935Mha) in 2100, following Moustakis et al. (2024). All other LULCC activities follow the BAU2030 scenario. In each region, we calculated the forest area change that occurred between 2015 and 2023, as well as the projected forest area change associated with other land-use change activities under the BAU2030 scenario over the period 2024–2100. These quantities were subtracted from the regional potential forest area; the remaining area represents the forest area increase required in each region over 2023–2100. This additional forest area is then allocated over time by assuming a linear increase in annual afforestation rates from 2024 to 2030, followed by a constant annual afforestation rate thereafter.

Stopping Gross Deforestation (Gross2030, Gross2050). This set of scenarios is motivated by the Glasgow declaration on forests, which aimed to halt the reduction of global forest area by 2030 but did not specify a clear pathway for achieving this target, which allowed two very distinct possible interpretations (Gasser et al., 2022). One approach to ensuring forest area stability is to halt all forest loss-inducing activities, while another approach involves balancing the rate of forest loss with the rate of forest gain. In the Gross2030 scenario, all forest-related LCCs reach zero by 2030. Shifting cultivation is phased out together with deforestation, while wood harvest follows the BAU2030 trajectory, since shifting cultivation involves burning young forests. All other LULCC activities follow the BAU2030 scenario. Recognizing the challenge of achieving this target by 2030, we also consider that the goal may be delayed to be reached by 2050, thereby defining the Gross2050 scenario.

Stopping Net Deforestation (Net2030, Net2050). The Net2030 scenario is the other interpretation of the Glasgow declaration on forest (Gasser et al., 2022), whereby we assume that the deforestation rate will decrease to match the afforestation rate of the BAU2030 by 2030, leading to forest area stability

after that, while other LULCC activities, including shifting cultivation and wood harvest, follow the BAU2030 scenario. Afforestation in the Net scenarios follows the BAU2030 trajectory, which is extrapolated from recent trends. As previously, the Net2050 scenario adopts a similar trajectory, but the target year is set to 2050 instead.

Line 154

Where is the afforestation in the NET scenarios? Is it also on pasture?

Response:

In the Net2030 and Net2050 scenarios, afforestation follows the BAU2030 trajectory. As in BAU2030, afforestation is derived from extrapolating recent regional trends (2013–2023) and is not restricted to a specific land-use type. The Net scenarios impose constraints only on deforestation, such that deforestation rates decline to match the BAU2030 afforestation rates by the target year, leading to net forest-area stability thereafter. We have clarified that afforestation in the Net scenarios follows BAU2030 assumptions regarding both magnitude and land-source flexibility.

The following is the associated revision in the Methods section (see lines 428-433).

Stopping Net Deforestation (Net2030, Net2050). The Net2030 scenario is the other interpretation of the Glasgow declaration on forest (Gasser et al., 2022), whereby we assume that the deforestation rate will decrease to match the afforestation rate of the BAU2030 by 2030, leading to forest area stability after that, while other LULCC activities, including shifting cultivation and wood harvest, follow the BAU2030 scenario. Afforestation in the Net scenarios follows the BAU2030 trajectory, which is extrapolated from recent trends. As previously, the Net2050 scenario adopts a similar trajectory, but the target year is set to 2050 instead.

Line 175

You should explain what these legacy emissions are. What are these emissions coming from deforested land? I assume they are due continuing to soil C emissions, but then when do you transfer this process to the forest regrowth category? I mean, when does the deforested land become categorized as regrowing forest?

Response:

By "legacy deforestation emissions" we refer to delayed carbon emissions that continue after deforestation has ceased, arising from the gradual decomposition of carbon stocks that were disturbed by past land-use change. These emissions originate primarily from soils and dead organic matter pools affected by historical deforestation, rather than from ongoing land conversion.

In OSCAR, carbon fluxes are tracked at the pool level rather than being reassigned to land-use categories over time. Following deforestation, carbon released from disturbed soil and litter pools contributes to legacy emissions, while vegetation

regrowth on the affected land contributes to the forest regrowth sink. These two processes can occur concurrently on the same land area, but are tracked separately. We have clarified this mechanism in the Results section to avoid confusion. More technical details are provided in Gasser et al. (2020) and Gasser and Ciais (2013).

The following is the associated revision in the Results section (see lines 185-188).

In the Commit scenario (**Fig. 5**j), legacy deforestation emissions persist due to delayed carbon losses from soils and dead organic matter pools disturbed by historical deforestation, but they are more than offset by forest regrowth and substantial reductions in emissions from wood harvesting and shifting cultivation, which yield a strong net carbon removal by 2100 (Supplementary Table 2).

Lines 175-211

Please put these numbers in a table or probably two tables; one for the global values and one for the regional values. They are awkward because there are so many.

Response:

We have moved the detailed numerical values to two new tables. Supplementary Table 2 summarizes the cumulative global contributions of individual E_{LUC} components across all LULCC scenarios (2024–2100), while Supplementary Table 3 reports cumulative regional E_{LUC} values for key regions. The Results section has been revised accordingly to focus on qualitative contrasts and dominant patterns, with explicit references to these tables.

The following is the associated revision in the Supplementary Information.

Supplementary Table 2. Cumulative contributions of E_{LUC} components across LULCC scenarios under low warming in 2024-2100 (Unit: Pg C).

Scenario	$E_{deforest}$	$S_{reforest}$	$E_{harvest} + S_{harvest}$	$E_{shifting} + S_{shifting}$	F_{others}	E_{LUC}
SSP1-1.9	36.7	-44.1	-3.4	-4.4	3.4	-12.0
SSP1-2.6	36.9	-44.2	-3.5	-4.6	1.7	-13.6
SSP2-4.5	40.9	-40.8	2.8	-3.9	10.0	9.0
SSP3-7.0	77.0	-32.1	1.9	-5.0	15.0	56.7
SSP4-3.4	121.1	-50.4	6.6	-7.2	19.4	89.4
SSP4-6.0	64.3	-36.6	7.4	-6.4	13.0	41.7
SSP5-3.4-OS	61.5	-38.6	6.1	-4.0	18.5	43.4
SSP5-8.5	45.7	-26.9	4.8	-3.7	11.0	30.8
Commit	12.7	-18.2	-22.0	-23.6	-1.2	-52.3
BAU2030	25.5	-24.1	1.3	11.4	-0.8	13.2
Forest2100	25.5	-109.6	1.3	11.4	-0.7	-72.2
Gross2030	14.9	-24.1	1.2	-20.9	-0.8	-29.7
Gross2050	20.8	-24.1	1.2	-20.4	-0.8	-23.2
Net2030	15.9	-24.1	1.3	11.4	-0.8	3.7
Net2050	21.2	-24.1	1.3	11.4	-0.8	9.0

Supplementary Table 3. Cumulative regional contributions of E_{LUC} across LULCC scenarios under low warming in 2024-2100 (Unit: Pg C). Regions include China (CHN), Indonesia (IDN), Brazil (BRA), Democratic Republic of the Congo (COD), United States (USA), Russia (RUS), India (IND), Angola (AGO), Peru (PER), Tanzania (TZA), and Colombia (COL).

Scenario	CHN	IDN	BRA	COD	USA	RUS	IND	AGO	PER	TZA	COL
SSP1-1.9	-8.9	-3.8	3.8	1.4	-1.7	-1.5	-0.8	1.4	0.2	0.6	-0.5
SSP1-2.6	-8.6	-4.0	3.4	1.5	-1.6	-1.7	-0.8	0.5	0.2	0.7	-0.4
SSP2-4.5	-9.0	0.5	6.0	5.2	-1.5	-1.5	-0.2	0.9	0.4	0.8	-0.6
SSP3-7.0	-12.7	-1.5	6.8	19.6	4.3	-1.9	1.3	4.2	5.5	1.3	0.0
SSP4-3.4	0.1	12.7	-18.4	22.8	5.4	-3.3	2.1	6.5	0.4	2.1	-1.1
SSP4-6.0	-4.3	-3.1	-8.2	19.5	2.8	-1.3	1.3	4.9	0.3	1.9	-0.5
SSP5-3.4-OS	-4.7	-1.6	11.6	9.7	6.1	-1.5	0.8	1.2	0.3	3.1	0.0
SSP5-8.5	-5.3	-3.0	10.8	8.6	3.3	-0.7	0.8	0.7	0.3	3.6	-0.6
Commit	-7.2	-6.1	-4.8	-2.1	-2.7	-2.6	-3.2	0.0	0.1	0.0	-1.2
BAU2030	-6.3	2.6	8.1	3.1	0.5	-0.7	-0.4	0.9	0.8	0.6	-0.4
Forest2100	-11.1	1.8	-5.9	0.3	-7.2	-3.6	-1.1	-2.1	0.8	0.4	-1.4
Gross2030	-7.1	-4.4	-2.0	-1.3	-0.1	-0.7	-1.0	0.3	0.2	0.3	-1.6
Gross2050	-6.7	-4.2	-0.2	-0.7	0.3	-0.7	-0.9	0.5	0.4	0.4	-1.4
Net2030	-6.8	2.5	4.6	2.5	-0.1	-0.7	-0.4	0.5	0.5	0.5	-0.6
Net2050	-6.5	2.6	6.6	2.9	0.2	-0.7	-0.4	0.7	0.7	0.6	-0.5

The following is the associated revision in the Results section (see lines 170-178).

Both Gross2030 and Gross2050 scenarios change into strong removals, following trajectories similar to Commit but with delayed peaks (**Fig. 5**f-g). Their cumulative removals reach −30 Pg C and −23 Pg C (Supplementary Table 2). It reflects that delaying the cessation of gross deforestation allows additional emissions to occur in the first decades. By contrast, the Net2030 and Net2050 scenarios permit continued gross deforestation balanced by afforestation, while following the same BAU2030 LULCC trajectory as the Gross scenarios for all non-targeted activities (**Fig. 5**h-i). The resulting cumulative fluxes are limited to 4 Pg C and 9 Pg C, respectively. These results indicate that policy stringency is the primary determinant of long-term land-use carbon benefits, while earlier implementation further enhances cumulative removals by shortening the period of avoidable emissions. Net-zero deforestation targets cannot replicate the mitigation potential of gross-zero strategies, even when implemented over shorter timescales.

The following is the associated revision in the Results section (see lines 185-207).

In the Commit scenario (**Fig. 5**j), legacy deforestation emissions persist due to delayed carbon losses from soils and dead organic matter pools disturbed by historical deforestation, but they are more than offset by forest regrowth and substantial reductions in emissions from wood harvesting and shifting cultivation, which yield a strong net carbon removal by 2100 (Supplementary Table 2). Under BAU2030, continued land conversion until 2030 leads to a nearly neutral land-cover-change balance, while additional emissions from shifting cultivation and wood harvesting result in a modest cumulative carbon source. The persistence of agricultural expansion and logging prevents large-scale recovery even after LULCC has stabilized. In Forest2100, large-scale forest expansion dominates the global carbon balance, producing the strongest cumulative removals despite continued deforestation and small emissions from harvest and shifting cultivation. The Gross2030 scenario substantially reduces deforestation-related emissions and enhances net removals, while delaying gross-zero deforestation to 2050 weakens the mitigation effect. In contrast, the Net2030 and Net2050 scenarios deliver only limited net removals, as continued shifting cultivation and wood harvesting constrain the overall mitigation potential (Supplementary Table 2).

At the regional scale, the Commit scenario results in widespread carbon removals, with particularly strong cumulative removals in the major forested and regrowing regions (**Error! Reference source not found.**a; Supplementary Table 3). Under BAU2030, pronounced contrasts persist between tropical source regions dominated by deforestation and temperate or boreal sink regions, broadly reflecting historical land-use patterns (**Error! Reference source not found.**b). The Forest2100 scenario produces the strongest regional removals overall, although some tropical regions remain weak carbon sources despite extensive afforestation (**Error! Reference source not found.**c). Gross2030 leads to marked reversals across many tropical regions, transforming major historical carbon sources into sinks, while Gross2050 exhibits similar but weaker patterns (**Error! Reference source not found.**d, e). In the Net2030 and Net2050 scenarios, several temperate and boreal regions continue to act as sinks, but persistent tropical deforestation causes many low-latitude regions to remain net carbon sources, dominating the global spatial pattern of E_{LUC} (**Error! Reference source not found.**f, g; Supplementary Table 3).

Implications

Line 225

This type of comparison is problematic because different countries/regions have different areas. To better compare between countries/regions these values should be normalized by area.

Response:

We agree that direct comparisons of absolute cumulative fluxes across regions can be misleading. However, a per area-based comparison is not relevant either. We have removed the comparison of the absolute gap between the largest sink and source in the Discussion.

Methods

Line 271

I think Table S1 is actually extended data table 1.

Response:

Thank you for noting this. The reference has been removed from the revised manuscript.

Line 274

Also ED table 1?

Response:

Yes, this is also Supplementary Table 1. We have corrected the reference accordingly

in the revised manuscript.

Lines 279-280

So what is your definition of Flasc here, and how does it differ from Dorgeist?

Response:

We have clarified the definition of F_{LASC} in the Methods section. In OSCAR, F_{LASC} represents the loss of additional sink capacity (LASC) induced by land-use change, defined as the difference in the carbon uptake capacity of natural vegetation between preindustrial and actual, evolving land cover conditions (Gasser and Ciais, 2013). This definition differs from that used in Dorgeist et al. (2024), where LASC is defined as the residual (i.e. a synergistic term) in a factorial experimental framework. Their LASC combines the loss of sink capacity with an additional interaction term, which is accounted for within E_{LUC} in our framework. The revised text now explicitly states this distinction.

The following is the associated revision in the Methods section (see lines 287-293).

$$S_{land,actual} = S_{land,PI} + F_{LASC} \tag{2}$$

where $S_{land,PI}$ represents the natural land sink under preindustrial land cover and F_{LASC} refers to the loss of additional sink capacity (LASC) due to land use changes. OSCAR characterizes F_{LASC} as the difference between the carbon uptake capacity of natural vegetation under preindustrial land cover and that under actual evolving land cover (Gasser and Ciais, 2013). This formulation of LASC corresponds to what has recently been termed Replaced Sources and Sinks (RSS) in the GCB-related paper (Friedlingstein et al., 2026). By contrast, Dorgeist et al. (2024) define LASC as a residual (i.e., synergistic) term within a factorial experimental design, which aggregates the loss of sink capacity with an interaction component that is treated separately as part of E_{LUC} in our framework.

Lines 285-292

So which of these do you use in this paper (presumably the first one?), and is Elcc the same in both cases? And how is the other definition relevant to this paper?

Response:

We use a single definition of E_{LCC} throughout this paper, and its total magnitude is identical in all cases. The two equations presented in the Methods describe alternative and mutually exclusive decompositions of the same E_{LCC}, not different fluxes.

All analyses and results in this study rely exclusively on the forest-focused decomposition, which separates E_{LCC} into contributions from deforestation, afforestation/reforestation, and a residual term grouping other land-cover transitions.

The alternative decomposition that focused on anthropogenic biomes (cropland and

pasture) is not used in the results discussed in the paper. It is provided for completeness and to facilitate the reuse of the dataset by users interested in agricultural or anthropogenic land-use dynamics. We have clarified this explicitly in the revised Methods text.

The following is the associated revision in the Methods section (see lines 302-309).

We offer two possible (but incompatible) breakdowns of E_{LCC} that could be of interest to users. Both decompositions sum to the same total E_{LCC} and differ only in the attribution of subcomponents. One focused on forest, whereby we isolate the flux from deforestation ($E_{deforest}$) and the one from afforestation/reforestation ($S_{reforest}$), grouping all other LCC transitions as the remainder (F_{others}):

$$E_{LCC} = E_{deforest} + S_{reforest} + F_{others} \quad (5)$$

Another one focused on anthropogenic biomes, which is not used in the results presented here but is documented for completeness and for users interested in agricultural land-use dynamics. We isolate the fluxes induced by the establishment or abandonment of cropland or pasture, as well as by other LCC between natural land types or anthropogenic land types, and a different remainder:

$$E_{LCC} = E_{cropland} + E_{pasture} + S_{cropland} + S_{pasture} + F_{natLCC} + F_{antLCC} + F_{rest} \quad (6)$$

Line 293

Eluc/forest cannot be defined by fluxes shown in eq 3-5?

Response:

$E_{LUC}^{biome=forest}$ cannot be reconstructed from the aggregate land carbon fluxes defined in Eqs. (3–5). Equations (3–5) describe the total land–atmosphere carbon exchange and its main components at the global land-system level. In contrast, $E_{LUC}^{biome=forest}$ are biome-specific bookkeeping fluxes that are diagnosed internally in OSCAR by tracking land-cover transitions and carbon pools at the biome level. As such, these forest-related subcomponent fluxes represent an additional level of disaggregation and cannot be inferred from the aggregated fluxes alone.

Lines 294-295

Does S* mean that you separate this forest flux for both PI and actual?

Response:

We have corrected the definition in the revised manuscript to make clear that this flux represents the natural land sink under preindustrial land cover occurring in forest biomes only.

Lines 355-362

This shifting is unclear. In the previous section on historical initialization to extrapolate 2016-2020 trends to 2023. But here it sounds like you then shift 2015 ssp data to 2024, which does not align the data at all at the beginning of your simulation. This would actually revert your forcings back 8 years and create a different discontinuity. So you either exacerbate the discontinuity, or your explanation is insufficient.

It also depends on whether you are applying the annual state data, or the transition data as your lulcc forcing. There are simple methods either way to align your forcing across the boundary.

Response:

We use transition data from LUH2-GCB (i.e., annual land-use changes) rather than annual state data. This allows us to apply the "shifted SSP" approach while preserving the internally consistent rates of change from the original IAM SSP narratives.

The time shift assume that 2015 policies are applied in 2024, so that the original IAM-derived land-use dynamics are applied at the end of our historical period (2023), ensuring continuity from the observation-constrained historical land-use state while maintaining the temporal evolution and relative transitions of the original SSP scenarios. We therefore adopt this shifting approach as a pragmatic and reproducible harmonization strategy, consistent with the philosophy of LUH2 (Hurtt et al., 2020), in the absence of officially updated SSP land-use datasets extending beyond 2015. We have revised the Methods section to clarify both the use of transition data and the rationale for the shift.

The following is the associated revision in the Methods section (see lines 376-392).

The LULCC data under eight SSP scenarios are taken from the LUH2 dataset for CMIP6 (Hurtt et al., 2020), representing internally consistent socio-economic pathways generated by IAMs rather than predictions tied to specific calendar years.

The LUH2 transition data under SSP scenarios are provided from 2015 onwards, whereas our simulations must start in 2024, immediately after the end of the observation-constrained historical period used in the GCB2024. To ensure continuity at the transition from historical land-use conditions, while preserving the internally consistent dynamics produced by IAMs, we apply a time shift to the SSP LULCC transition data. These methods leverage the SSP-derived annual LULCC rates starting from the 2023 observational baseline.

The first 77 years of each original SSP scenario (2015–2091) are reassigned 2024–2100. Historical LCC is simulated up to 2023, and the shifted SSP LCC data are then appended from 2024 onward. For LU components (wood harvest and shifting cultivation), an offset is applied using the mean of the last 11 years (2013–2023) as a reference, consistent with the treatment of CO_2 and climate forcings.

We recognize that this means the new LULCC data is inconsistent with other aspects of these scenarios (e.g., non-CO_2 emissions, population). Still, it is difficult to maintain consistency across all aspects while changing the starting year of a scenario. This approach preserves the rates of change, temporal evolution, and spatial patterns of land-use change embedded in each SSP narrative under IAM-based projections. Cumulative LULCC emissions from IAMs are taken over their native scenario period

(2015–2091) and used as a benchmark to contextualize OSCAR-based estimates under the corresponding shifted SSP land-use pathways, without serving as model inputs or constraints.

Reference

Dorgeist, L., Schwingshackl, C., Bultan, S., and Pongratz, J.: A consistent budgeting of terrestrial carbon fluxes, Nature Communications, 15, 7426, http://doi.org/10.1038/s41467-024-51126-x, 2024.

Friedlingstein, P., Le Quéré, C., O'Sullivan, M., Hauck, J., Landschützer, P., Luijkx, I. T., Li, H., van der Woude, A., Schwingshackl, C., Pongratz, J., Regnier, P., Andrew, R. M., Bakker, D. C. E., Canadell, J. G., Ciais, P., Gasser, T., Jones, M. W., Lan, X., Morgan, E., Olsen, A., Peters, G. P., Peters, W., Sitch, S., and Tian, H.: Emerging climate impact on carbon sinks in a consolidated carbon budget, Nature, 649, 98-103, 10.1038/s41586-025-09802-5, 2026.

Friedlingstein, P., O'Sullivan, M., Jones, M. W., Andrew, R. M., Hauck, J., Landschützer, P., Le Quéré, C., Li, H., Luijkx, I. T., Olsen, A., Peters, G. P., Peters, W., Pongratz, J., Schwingshackl, C., Sitch, S., Canadell, J. G., Ciais, P., Jackson, R. B., Alin, S. R., Arneth, A., Arora, V., Bates, N. R., Becker, M., Bellouin, N., Berghoff, C. F., Bittig, H. C., Bopp, L., Cadule, P., Campbell, K., Chamberlain, M. A., Chandra, N., Chevallier, F., Chini, L. P., Colligan, T., Decayeux, J., Djeutchouang, L. M., Dou, X., Duran Rojas, C., Enyo, K., Evans, W., Fay, A. R., Feely, R. A., Ford, D. J., Foster, A., Gasser, T., Gehlen, M., Gkritzalis, T., Grassi, G., Gregor, L., Gruber, N., Gürses, Ö., Harris, I., Hefner, M., Heinke, J., Hurtt, G. C., Iida, Y., Ilyina, T., Jacobson, A. R., Jain, A. K., Jarníková, T., Jersild, A., Jiang, F., Jin, Z., Kato, E., Keeling, R. F., Klein Goldewijk, K., Knauer, J., Korsbakken, J. I., Lan, X., Lauvset, S. K., Lefèvre, N., Liu, Z., Liu, J., Ma, L., Maksyutov, S., Marland, G., Mayot, N., McGuire, P. C., Metzl, N., Monacci, N. M., Morgan, E. J., Nakaoka, S. I., Neill, C., Niwa, Y., Nützel, T., Olivier, L., Ono, T., Palmer, P. I., Pierrot, D., Qin, Z., Resplandy, L., Roobaert, A., Rosan, T. M., Rödenbeck, C., Schwinger, J., Smallman, T. L., Smith, S. M., Sospedra-Alfonso, R., Steinhoff, T., Sun, Q., Sutton, A. J., Séférian, R., Takao, S., Tatebe, H., Tian, H., Tilbrook, B., Torres, O., Tourigny, E., Tsujino, H., Tubiello, F., van der Werf, G., Wanninkhof, R., Wang, X., Yang, D., Yang, X., Yu, Z., Yuan, W., Yue, X., Zaehle, S., Zeng, N., and Zeng, J.: Global Carbon Budget 2024, Earth Syst. Sci. Data, 17, 965-1039, http://doi.org/10.5194/essd-17-965-2025, 2025.

Gasser, T. and Ciais, P.: A theoretical framework for the net land-to-atmosphere CO₂ flux and its implications in the definition of "emissions from land-use change", Earth Syst. Dynam., 4, 171-186, http://doi.org/10.5194/esd-4-171-2013, 2013.

Gasser, T., Ciais, P., and Lewis, S. L.: How the Glasgow Declaration on Forests can help keep alive the 1.5 °C target, Proceedings of the National Academy of Sciences, 119, e2200519119, https://doi.org/10.1073/pnas.2200519119, 2022.

Gasser, T., Crepin, L., Quilcaille, Y., Houghton, R. A., Ciais, P., and Obersteiner, M.: Historical CO2 emissions from land use and land cover change and their uncertainty, Biogeosciences, 17, 4075-4101, https://doi.org/10.5194/bg-17-4075-2020, 2020.

Hurtt, G. C., Chini, L., Sahajpal, R., Frolking, S., Bodirsky, B. L., Calvin, K., Doelman, J. C., Fisk, J., Fujimori, S., Klein Goldewijk, K., Hasegawa, T., Havlik, P., Heinimann, A., Humpenöder, F., Jungclaus, J., Kaplan, J. O., Kennedy, J., Krisztin, T., Lawrence, D., Lawrence, P., Ma, L., Mertz, O., Pongratz, J., Popp, A., Poulter, B., Riahi, K., Shevliakova, E., Stehfest, E., Thornton, P., Tubiello, F. N., van Vuuren,

D. P., and Zhang, X.: Harmonization of global land use change and management for the period 850–2100 (LUH2) for CMIP6, Geosci. Model Dev., 13, 5425-5464, https://doi.org/10.5194/gmd-13-5425-2020, 2020.

Moustakis, Y., Nützel, T., Wey, H.-W., Bao, W., and Pongratz, J.: Temperature overshoot responses to ambitious forestation in an Earth System Model, Nature Communications, 15, 8235, https://doi.org/10.1038/s41467-024-52508-x, 2024.

Reviewer #1

I appreciate the authors' efforts in resolving my issues. I recommend publication.

Response:

We sincerely thank the reviewer for the positive feedback and for recommending the publication of our manuscript. We greatly appreciate the reviewer’s time and support, which helped improve the quality of this work.

Reviewer #2 (Remarks to the Author):

I thank the authors for their explanations and their numerous edits to both text and figures. I think the manuscript is much clearer as a result. I have one broader question about the implications of the study and a couple small suggestions about the revised text, which are listed below.

Response:

We thank the reviewer for the positive feedback and helpful suggestions. We address the broader question and the specific comments below.

Broad question

I understand the rationale for focusing on E_LUC for this paper, but according to Fig 1b, temperature and CO_2 concentrations matter quite a lot for the total land emissions. So how should I as a reader interpret the total change to emissions? One uncharitable interpretation would be that there is a negative feedback via S_land,actual (that is unaccounted for when using an offline land use model) that could offset the policy changes discussed in the rest of the paper. Another slightly more optimistic interpretation might be that we can make a dent in land use through policy, but that non-LU emissions will actually drive a lot of the LU carbon change. I think it would be really helpful to add some text - likely in the discussion but potentially elsewhere - that describes how the authors are thinking about the total impact.

Response:

We thank the reviewer for this important comment.

In the revised manuscript, we clarify that only carbon fluxes arising from direct anthropogenic land-use activities are attributed to land-use change (E_{LUC}), while the natural land sink (S_{land}) is treated as an indirect response to environmental change, primarily driven by atmospheric CO_2 increase and climate change. This distinction reflects the well-known definitional differences between model-based estimates and inventory-based accounting, and motivates our focus on E_{LUC} as the appropriate metric to assess the mitigation potential of land-use policies.

We agree with the reviewer that the interpretation of total land carbon fluxes (F_{net}) is important from the reader's perspective. We have therefore expanded the Discussion section to clarify how E_{LUC} and S_{land} jointly contribute to total changes in land carbon fluxes.

<u>The following is the associated revision in the Introduction section (see lines 36-44).</u>

National greenhouse gas inventories consider carbon fluxes occurring on managed land are anthropogenic, thereby combining E_{LUC} and S_{land} components [12, 13]. In contrast, model-based

frameworks distinguish between direct anthropogenic fluxes (E_{LUC}) and the natural land sink (S_{land}) [9, 14, 15]. This definitional mismatch leads to substantial differences between inventory-based accounting and model-based estimates. The Global Carbon Budget (GCB) addresses this discrepancy by combining bookkeeping model estimates of E_{LUC} [16, 17, 18] with S_{land} simulated by Dynamic Global Vegetation Models (DGVMs) [19, 20, 21]. Within this framework, E_{LUC} is interpreted as the component of land carbon flux directly attributable to anthropogenic land-use activities, while S_{land} is treated as an indirect response driven by atmospheric CO_2 increase and climate change. This approach facilitates a dialogue between the scientific and policy communities [4, 13], but is possible in the future only with a model that consistently represents both terms of the equation [22].

The following is the associated revision in the Discussion section (see lines 250-258).

Total land carbon fluxes (F_{net}) reflect the combined effects of LULCC on E_{LUC} and of atmospheric CO_2 and climate change on the S_{land}. While the latter dominates the magnitude of total flux changes, it is largely externally driven and therefore does not reflect mitigation from land-use policy. E_{LUC} remains the appropriate metric to assess the impact of land-use interventions. This study assumes the impacts of LULCC on land carbon fluxes are independent of the biogeophysical effects of LULCC on climate. In reality, interactions between LULCC and climate influence the natural land sink, as LULCC modifies surface radiation, energy and water balances, and regional meteorology, thereby altering temperature, moisture availability, fire activity, and atmospheric CO_2 [1]. The comparison between low-warming and high-warming scenarios indicates that this does not affect the main conclusions of this study, as LULCC-driven carbon fluxes are dominated by LULCC rather than by climate.

Smaller comments

L384: This sentence is too vague given that not everything about the SSP is reassigned: “The first 77 years of each original SSP scenario (2015－2091) are reassigned 2024－2100.” I think it should specify what has been shifted and what has not.

Response:

We have clarified that the LULCC data are shifted.

The following is the associated revision in the Methods section (see lines 382-384).

The first 77 years of LULCC data under each original SSP scenario (2015–2091) are reassigned to 2024–2100. Historical LCC transition data is simulated up to 2023, and the shifted SSP LCC transition data are then appended from 2024 onward.

L388-389 (and also L258): The parenthetical states population and non-CO_2 emissions, which is true. But the study is not really impacted by either of those. I think it would make more sense for it to list the relevant variables like temperature or

CO_2 concentrations (which are driven largely by non-land-use emissions and presumably still follow the non-shifted SSP).

Response:

We have revised the text to refer explicitly to climate and CO_2 forcing.

The following is the associated revision in the Methods section (see lines 386-387).

We acknowledge that this shifting breaks consistency with other aspects of the SSP scenarios (e.g., climate, CO_2 concentrations), but it is difficult to retain consistency of all aspects while changing the starting year of a scenario.

Reference

1. Canadell JG, *et al.* From global to national GHG budgets: the REgional Carbon Cycle Assessment and Processes-3 (RECCAP3). *National Science Review* **12**, nwaf037 (2025).
2. Grassi G, *et al.* Harmonising the land-use flux estimates of global models and national inventories for 2000–2020. *Earth Syst Sci Data* **15**, 1093-1114 (2023).
3. Grassi G, *et al.* Reconciling global-model estimates and country reporting of anthropogenic forest CO2 sinks. *Nature Climate Change* **8**, 914-920 (2018).
4. Grassi G, *et al.* Critical adjustment of land mitigation pathways for assessing countries' climate progress. *Nature Climate Change* **11**, 425-434 (2021).
5. He Y, Piao S, Ciais P, Xu H, Gasser T. Future land carbon removals in China consistent with national inventory. *Nature Communications* **15**, 10426 (2024).
6. Friedlingstein P, *et al.* Global Carbon Budget 2023. *Earth Syst Sci Data* **15**, 5301-5369 (2023).
7. Houghton RA, Nassikas AA. Global and regional fluxes of carbon from land use and land cover change 1850–2015. *Global Biogeochemical Cycles* **31**, 456-472 (2017).
8. Hartung K, *et al.* Bookkeeping estimates of the net land-use change flux – a sensitivity study with the CMIP6 land-use dataset. *Earth Syst Dynam* **12**, 763-782 (2021).
9. Foley JA, Levis S, Prentice IC, Pollard D, Thompson SL. Coupling dynamic models of climate and vegetation. *Global Change Biology* **4**, 561-579 (1998).
10. Kirilenko A. Modeling Dynamic Vegetation Response to Rapid Climate Change Using Bioclimatic Classification. *Climatic Change* **38**, 15-49 (1998).
11. Sitch S, *et al.* Trends and Drivers of Terrestrial Sources and Sinks of Carbon Dioxide: An Overview of the TRENDY Project. *Global Biogeochemical Cycles* **38**, (2024).
12. Friedlingstein P, *et al.* Global Carbon Budget 2024. *Earth Syst Sci Data* **17**, 965-1039 (2025).
13. Gidden MJ, *et al.* Aligning climate scenarios to emissions inventories shifts global benchmarks. *Nature* **624**, 102-108 (2023).
14. Foley JA, *et al.* Global Consequences of Land Use. *Science* **309**, 570-574 (2005).

Reviewer #3 (Remarks to the Author):

Overall response

The authors have done a good job addressing the reviewers' comments. I recommend publication after addressing the minor issues below.

Response:

We thank the reviewer for the positive assessment and for the helpful suggestions provided below.

Specific comments/suggestions

Abstract
line 15: be explicit here: LULCC emissions

Response:

We have revised the text to explicitly refer to LULCC emissions.

The following is the associated revision in the Abstract section (see lines 14-15).

Deforestation and forest regrowth dominate variability in LULCC CO_2 emission, with policy timing and ambition exerting strong control.

Introduction

line 23: land use and land-cover change

Response:

We have corrected it to land-use and land-cover change.

Results

lines 75-76:

Thank you for making the flux notation more consistent. I suggest in this first reporting you explicitly state the meaning of the sign: negative is land uptake and positive is emissions.

Response:

We have added a sentence clarifying the sign convention.

The following is the associated revision in the Results section (see lines 73-74).

Here, negative values indicate carbon removals from the atmosphere, while positive values indicate

emissions.

line 90:

"further contribute to variations" is vague

you can state that these values demonstrate some interaction between land cover change and climate with respect to $S_{land, actual}$

Response:

We have revised the sentence to describe the interaction between land-cover change and climate in shaping $S_{land, actual}$.

The following is the associated revision in the Discussion section (see lines 90-92).

Meanwhile, the loss of additional sink capacity (F_{LASC}) differs between the SSP1-2.6 (16 Pg C under low warming and 23 Pg C under high warming) and SSP3-7.0 (18 Pg C and 35 Pg C) LULCC scenarios, demonstrating an interaction between LULCC and climate in shaping $S_{land,actual}$.

Discussion

lines 247-253:

you should also note here the interactions between lulcc and climate that influence $S_{land, actual}$.

Response:

We have added a sentence noting that interactions between LULCC and climate can influence $S_{land, actual}$.

The following is the associated revision in the Discussion section (see lines 254-256).

In reality, interactions between LULCC and climate influence the natural land sink, as LULCC modifies surface radiation, energy and water balances, and regional meteorology, thereby altering temperature, moisture availability, fire activity, and atmospheric CO_2 [1].

Methods

line 369:

I appreciate supplementary figure 2

Response:

We thank the reviewer for the positive comment.

lines 374-390:

I understand now how you are shifting the lulcc scenarios, but I am not following the reasoning when I read it. E.g., If the shift makes the lulcc inconsistent with some of the drivers of the IAMS, and also with the climate forcing, how is this preserving the "rates of change, temporal evolution, and spatial patterns of land-use change embedded in each SSP narrative under IAM-based projections"?

I think what you are trying to say is that when considering just lulcc, you want to preserve the scenario specifics as they start from a historical ending point.

The challenge is that the projections are not independent of the starting point or the RF-specific trajectory (which do tie the lulcc rates to specific years). This is just a fact of both how models work and the lulcc needs for meeting the RF targets.

Also, the shift appears to not matter much anyway, based on the figure you provided in the response.

So I think it is easier to justify aligning the lulcc rates by year, rather than shifting them, because you align your other inputs by year.

You can however, make a case for shifting, but it needs to be more clear.

Consider expanding on your justification to explain the fact that the scenarios generate projections from an historical starting point, and because you advanced your historical ending point (which you have justified) the overlapping lulcc transitions are not consistent with each other. Given the historical dependence of lulcc you want to maintain the change trajectory of the scenario, but apply it starting at your updated historical ending point.

Response:

We thank the reviewer for this insightful comment and agree that SSP land-use projections are not independent of their starting point or associated forcing trajectories.

Advancing the historical endpoint to 2023 inevitably creates an inconsistency with the initial years of the original SSP projections, and any transformation breaks some aspects of the original scenario coherence. Our objective is to retain the short-term dynamics and scenario-specific divergence in LULCC pathways while applying them from an updated historical baseline. As suggested by the reviewer, we have revised the Methods section to more clearly describe the rationale and implementation of the shifting approach.

The following is the associated revision in the Methods section (see lines 375-393).

The LULCC data for the eight SSP scenarios are taken from the LUH2 dataset for CMIP6 [27], which provides internally consistent socio-economic pathways derived from integrated assessment models (IAMs). The LUH2 data under SSP scenarios are provided from 2015 onwards, whereas our simulations must start in 2024, immediately after the end of the observation-constrained historical period used in the GCB2024. Advancing the historical endpoint to 2023 creates an inconsistency between the observed LULCC state and the initial years of the SSP projections, which are generated from an earlier historical baseline.

To address this mismatch, we apply a time shift to the LULCC data, such that the scenario-specific trajectories are initiated from the 2023 observational baseline. The first 77 years of LULCC data under each original SSP scenario (2015–2091) are reassigned to 2024–2100. Historical LCC transition data is simulated up to 2023, and the shifted SSP LCC transition data are then appended from 2024 onward. For LU components (wood harvest and shifting cultivation), an offset is applied using the mean of the last 11 years (2013–2023) as a reference, consistent with the treatment of CO_2 and climate forcings.

We acknowledge that this shifting breaks consistency with other aspects of the SSP scenarios (e.g., climate, CO_2 concentrations), but it is difficult to retain consistency of all aspects while changing the starting year of a scenario. The early phase of SSP scenarios typically exhibits the strongest divergence in LULCC trajectories, reflecting differences in socio-economic pathways, whereas later periods tend to show more gradual and convergent changes. Our objective is therefore to apply the short-term dynamics and narrative divergence embedded in SSP LULCC pathways from an updated historical endpoint, although relaxing their association with specific calendar years. This approach provides a pragmatic compromise that maintains scenario-specific land-use narratives while ensuring continuity with observation-constrained historical conditions.

Reference

1. Foley JA*, et al.* Global Consequences of Land Use. *Science* **309**, 570-574 (2005).
2. Hurtt GC*, et al.* Harmonization of global land use change and management for the period 850–2100 (LUH2) for CMIP6. *Geosci Model Dev* **13**, 5425-5464 (2020).
